# First total synthesis of ampullosine, a unique isoquinoline alkaloid isolated from *Sepedonium ampullosporum*, and of the related permethylampullosine†

**DOI:** 10.1039/c9ra06839b

**Published:** 2019-10-16

**Authors:** Didier F. Vargas, Enrique L. Larghi, Teodoro S. Kaufman

**Affiliations:** Instituto de Química Rosario (IQUIR, CONICET-UNR), Facultad de Ciencias Bioquímicas y Farmacéuticas, Universidad Nacional de Rosario Suipacha 531 S2002LRK Rosario Argentina Kaufman@iquir-conicet.gov.ar larghi@iquir-conicet.gov.ar

## Abstract

A straightforward and convenient approach toward the first total synthesis of ampullosine, a structurally unique 3-methylisoquinoline alkaloid isolated from *Sepedonium ampullosporum*, is reported. Access to the related *O*-methyl ampullosine methyl ester from a common intermediate is also disclosed. The synthetic sequence toward the natural product comprised a Kolbe-type carboxylation of 3,5-dihydroxybenzoic acid and further esterification of the diacid, followed by masking of one of the phenols through selective ester reduction and subsequent acetonide formation. Installation of the three-carbon atom required for the 3-methylpyridine ring was performed by triflation of the remaining free phenol and a Pd-catalyzed Suzuki–Miyaura reaction with potassium *E*-propenyltrifluoroborate. Deprotection of the acetonide, followed by partial oxidation of the benzylic alcohol to the salicylaldehyde, *O*-methylation of the free phenol and hydrazonation of the resulting *ortho*-anisaldehyde derivative gave a hydrazone-based 1-azatriene. This was further subjected to 6π-azaelectrocyclization to afford permethylampullosine (11 steps, 14% overall yield), whereas exhaustive demethylation with AlI_3_ generated *in situ* gave ampullosine (12 steps, 3.2% global yield).

## Abstract 2

The first total synthesis of ampullosine, a unique isoquinoline isolated from *Sepedonium ampullosporum*, is reported. The related permethylampullosine was also synthesized.

## Graphical abstract

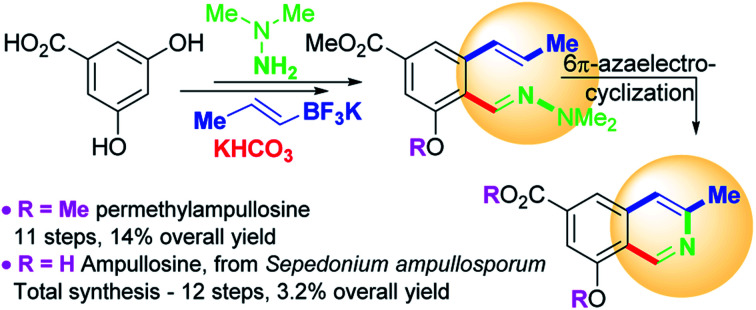

## Introduction

The genus *Sepedonium* (Ascomycetes) is saproparasitic and comprises asexual states of fungi that parasitize on other fungi; more specifically basidiocarps of the mushroom order Boletales.^1^ They operate by first settling on their fleshy living hosts, which they kill to employ the organic matter as nutrients to grow and develop. These fungicolous species have a mould-like aspect, with thick-walled gold-yellow colored aleurioconidia and colorless thin-walled phialoconidia.

The genus was established at the beginning of the XIX century. However, the seminal works of Petch and Pieschel^2^ described variations of *S. chrysospermum*, namely *S. ampullosporum*,^3*a*^*S. chalcipori*,^3*b*^ and *S. microspermum*,^3*c*^ which along with *S. laevigatum* ended up conforming autonomic species on the basis of sequence analysis of the internal transcribed spacer region of the nuclear ribosomal RNA genes.^1^

**Fig. 1:**
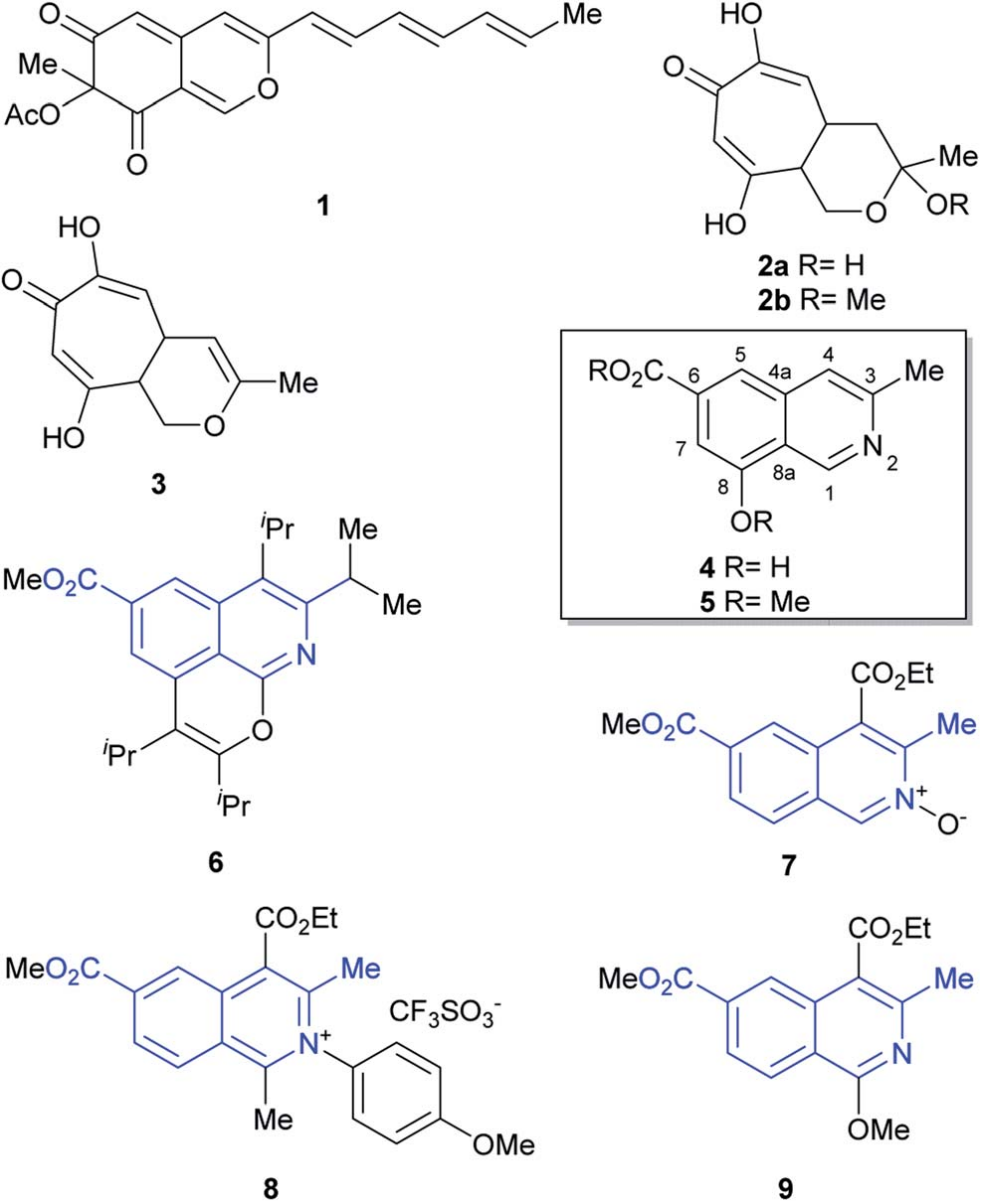
Chemical structures of some polyketides (1–3) and ampullosine (4), isolated from *Sepedonium* species, as well as the semisynthetic ampullosine derivative (5) and structurally related, synthetic 3-methyl 6-carboxymethyl isoquinolines (6–9).

Fungi of the genus *Sepedonium* have been studied as producers of antibiotics and pigments, as well as for their properties as antagonist agents of various plant pathogens. Chemical investigations of *Sepedonium* spp. revealed that this species is an interesting source of various natural products with potential biopharmaceutical applications (Fig. 1), including peptaibols, uncommon peptides with a broad spectrum of biological activities,^4^ the unusual cyclic peptide chrysosporide,^5^ the azaphilone chrisodin (1),^6^ as well as some mono- and bis-anthraquinones (skyrin, rugulosin, chrysophanol)^1,7^ and tropolone derivatives (2–3),^8^ among others.^5^

Infected organisms are rarely colonized by secondary fungal parasites, to compete with *Sepedonium* for host nutrients. This observation supports the speculation that *Sepedonium* species produce small defense molecules that prevent further infections.

Recently, the group of Arnold examined the culture extract of *S. ampullosporum* and isolated ampullosine (4).^9^ This alkaloid, responsible for the deep yellow color of its culture fluid, is the first and only isoquinoline isolated from the genus *Sepedonium*. Its structure was established by spectroscopic means.

In addition, methylation of the heterocycle afforded *O*-methyl ampullosine methyl ester (5), which contributed to the identification of the natural product. Employing LC/ESI, the authors were able to detect ampullosine in ten out of twelve strains of *Sepedonium*, belonging to eight different species. However, they observed that the two most phylogenetically distant species lacked the alkaloid.

The structure of ampullosine is unique. Despite some 3-methylisoquinolines have been synthesized, the combination of this motif with a 6-carboxylic acid moiety is rare; further, although they are seldom found in patents,^10^ a few and scattered examples started to appear in the literature just recently, favored by the flourishment of research into C–H activation reactions based on ruthenium (6)^11^ and rhodium (7–9)^12^ catalysis. In many cases, however, the current limitations of this novel chemistry still turn unfavorable its use to approach ampullosine.

We have been interested in the synthesis of structurally unique heterocyclic natural products^13^ and have used the 6π-electrocyclization of 1-azatrienes as an efficient and atom-economic strategy toward the synthesis of natural products or their analogs, containing the 3-methyl isoquinoline motif.^14^

**Scheme 1:**
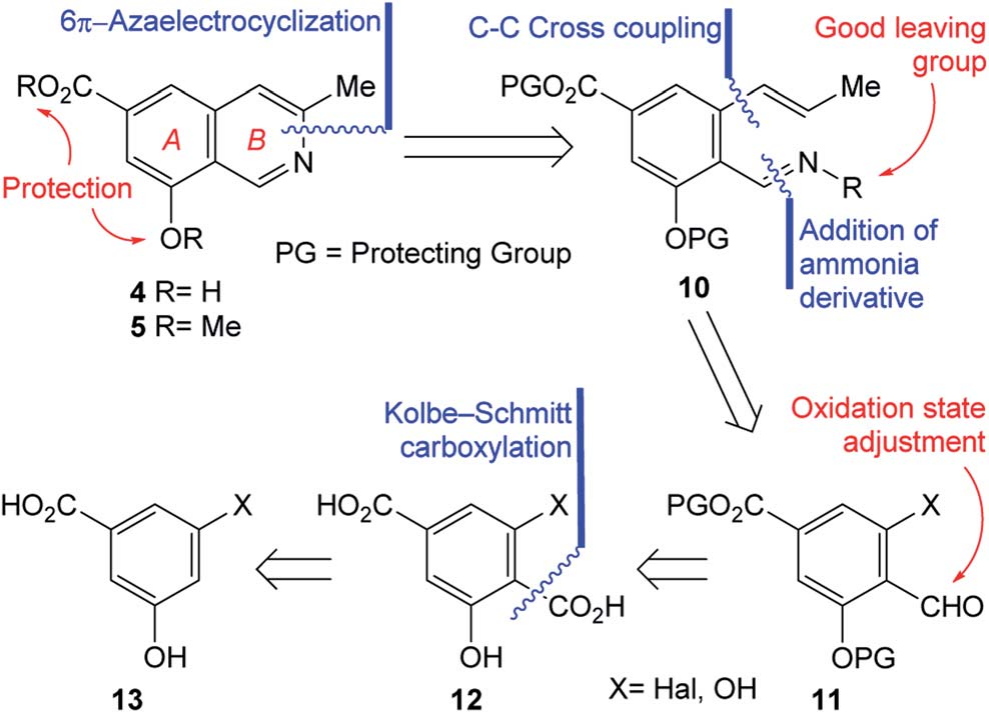
Retrosynthetic analyses of ampullosine (4) and its derivative 5.

In pursuit of these interests, herein we wish to report the first total synthesis of ampullosine (4) and the synthesis of *O*-methyl ampullosine methyl ester (5), from a common intermediate, using the easily available 3,5-dihydroxybenzoic acid and employing a 6π-azaelectrocyclization reaction as the key transformation, according to the retrosynthetic analysis depicted in Scheme 1.

## Results and discussion

The initial N–C3 disconnection of the analysis was performed on 4 and 5 and unveiled the polysubstituted benzenoid 10 as a fit key intermediate for the proposed 6π-azaelectrocyclization based ring closure toward both targets. The main features of 10 are a properly masked carboxyl group, a differentially protected phenolic moiety, according to the final targets and an *ortho*-disubstituted carbonyl condensed with an ammonia derivative which carries a suitable leaving group.

A couple of additional disconnections were carried out on 10 to uncover the salicylaldehyde derivative 11. The reasoning was that condensation with an ammonia derivative (oxime, hydrazone, *etc.*) could provide the required activated nitrogen atom for the pyridine ring of the target; in addition, it was conjectured that a suitable cross-coupling reaction with an allyl/propenyl derivative^15^ could perform the substitution of a properly located halogen or activated phenol, enabling the installation of the three carbon atoms chain, required to build the heterocyclic ring.

Further analysis of 11 determined that its substituent pattern could be advantageously simplified towards a suitable starting material, by considering an oxidation state adjustment of the aldehyde. This placed terephthalic acid derivative 12 in the path toward the natural product. In turn, the *ortho*-disubstituted carboxylic acid was disconnected, to reveal compound 13 as an apt starting material, considering the availability of protocols to access salicylic acid derivatives from phenols.^16^

Interestingly, despite different 3-halobenzoic acids have been reported in the literature,^17^ they exhibited some accessibility drawbacks; among them, lack of suitable precursors or efficient transformations from commercial materials. Therefore, it was concluded that among the possible candidates to fulfil the odd structural requirements imposed by the retrosynthetic analysis, only 3,5-dihydroxybenzoic (14) is inexpensive, easily available and displays the most ideal substitution pattern.

On the other hand, despite various methodologies are available for the *ortho* formylation of phenols, some protocols such as Duff, Skattebøl, Reimer–Tiemann, Vilsmeier–Haack and Gattermann–Koch require activated substrates, while others (*ortho* metalation) demand protection of the sensitive groups, such as the carboxyl moiety, and all of them may afford mixtures of isomeric products.^18^ Furthermore, some literature precedents suggested that formylation of 13 may result in the undesired formyl derivative, and in low yield.^19^

**Scheme 2:**
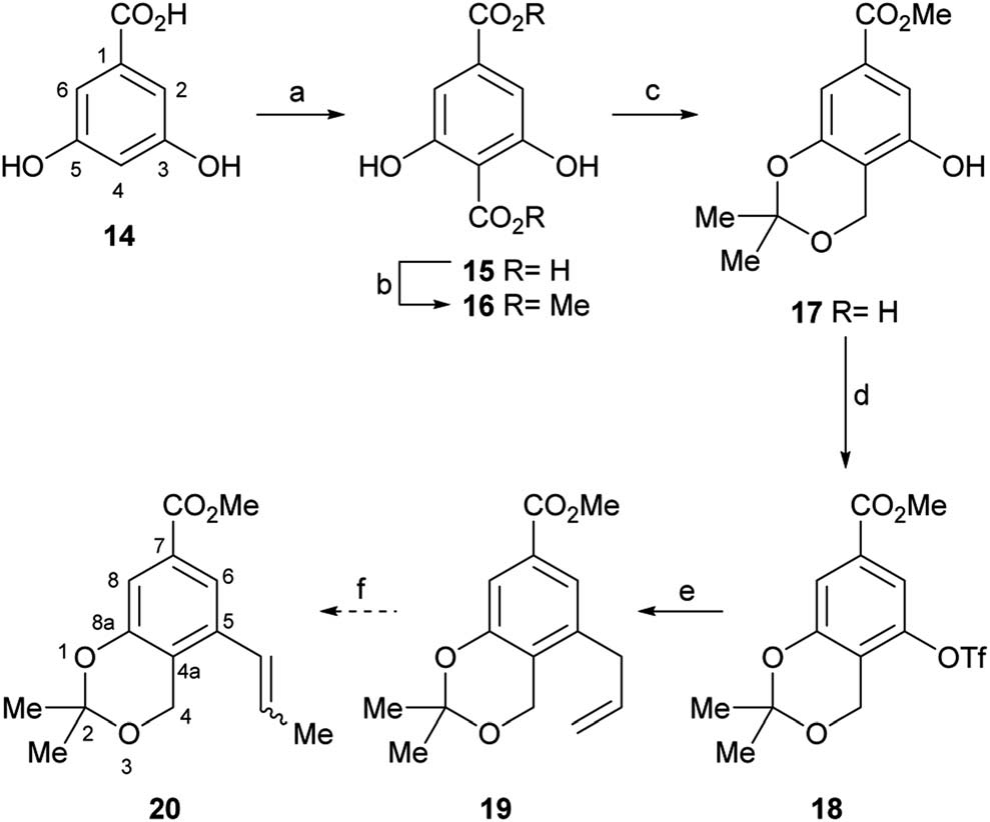
Reagents and conditions: (a) CO_2_ (sealed tube), KHCO_3_, 1,2-propanediol, 180 °C, 6 h; (b) Me_2_SO_4_, KHCO_3_, acetone, reflux, 10 h, (70% overall); (c) (1) NaBH_4_, THF/H_2_O (4 : 1), 0.1 M phosphate buffer pH 7.5, 0 °C→r.t., 1.5 h; (2) 2,2-DMP, TsOH, r.t., 5 h (75% overall); (d) PhNTf_2_, DMAP, NEt_3_, CH_2_Cl_2_, r.t. 16 h, (96%); (e) ^*n*^Bu_3_SnCH_2_CH=CH_2_, PdCl_2_(PPh_3_)_2_, PPh_3_, LiCl, diglyme, 125 °C, 15 h (19 and 20, 95 : 5, 70% global); (f) RuClH(CO)(PPh_3_)_3_ (cat.), PhMe, 80 °C.

Therefore, the synthesis commenced with 14 (Scheme 2), which was submitted to a Kolbe–Schmitt type carboxylation with KHCO_3_ at 180 °C, under a CO_2_ atmosphere (balloon).^20^ It was observed that the use of glycerol as reaction medium resulted in a deep red coloured gummy mass, which difficulted extraction and product purification, affording 15 in poorly reproducible yields, below 60%. Luckily, however, employing a minimum amount of propylene glycol (final concentration of 14 was ∼4 mol L^−1^) as solvent provided 15 as an easily recoverable pale yellow solid in better yield (80%).

Furthermore, similar performances were observed when the reaction was carried out in a closed vessel, purged with CO_2_, a requirement of the reaction mechanism.^21^ Without purification, the resulting solid mass containing the diacid product 15 was submitted to selective esterification with Me_2_SO_4_ providing the dimethyl ester 16 (70% yield for the two steps).

In turn, the diester was selectively reduced with NaBH_4_, under anchimeric assistance provided by the neighbouring phenol moieties to the *ortho*-disubstituted C4 carboxylate, and the resulting benzyl alcohol was converted to the acetonide 17 in 75% overall yield.

In order to install the projected three carbon atom chain for the heterocyclic ring, compound 17 was next activated toward a Stille cross-coupling reaction, by triflate ester formation with PhNTf_2_. The latter reagent was elected over the more reactive Tf_2_O after considering previous failures of the anhydride in similar transformations.^22*a*^

Luckily, the reaction of 17 with *N*-phenyl triflimide, employing Et_3_N as base and DMAP as nucleophilic substitution catalyst, furnished 96% of the triflate 18.^22*b*,*c*^ Interestingly chromatographic elution with hexane:CH_2_Cl_2_ mixtures proved critical to obtain the product free from *N*-phenyltriflamide, a by-product of the transformation.

In continuation of the sequence, the triflate was submitted to a Stille cross-coupling reaction with ^*n*^Bu_3_SnCH_2_CH=CH_2_ in diglyme, in the presence of LiCl and under PdCl_2_(PPh_3_)_2_ catalysis. This afforded the expected allyl-substituted compound 19 in mixture (95 : 5) with the propenyl derivative *E*-20 in combined 70% yield, as observed in its ^1^H NMR spectrum. However, despite our experience, it was found comparatively difficult to obtain clean samples of 19, devoid of undesirable contamination with tin residues, which somehow hindered a proper development of the projected isomerization step toward 20.

Therefore, an alternative was sought. Fortunately for our needs, due to their improved air-stability, reduced cost and improved nucleophilicity, the use of organotrifluoroborate salts has emerged^23^ as an excellent alternative to the boronates and boronic acids, expensive conventional substrates for the Suzuki–Miyaura reactions.^24^ Their advantages are more evident when it is also considered that vinylboronic acids often require excess reagent to achieve better yields^25^ and suffer from stability issues, tending to polymerize.^26^

Therefore, the modification of the Suzuki–Miyaura reaction pioneered by Molander was implemented, and the cross-coupling of 18 was performed with potassium propenyl trifluoroborate (21)^27^ and Cs_2_CO_3_ as base. The reaction required optimization (Table 1), since the initial results with Pd(OAc)_2_ as the metal source and DavePhos as ligand afforded only moderate yields of 20 (53–58%) when the reaction was run in a toluene-water system (4 : 1, v/v) at 90–100 °C during 16–24 h, respectively (entries 1 and 2).

**Table 1:** Optimization of the propenylation of the triflate 18^a^

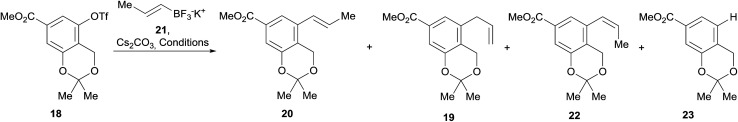										
Run no.	Catalyst	Ligand	Solvent	Temp. (°C)	Time (h)	Yield (%)				
						18	19	20	22	23
1	Pd(OAc)_2_	DavePhos	PhMe/H_2_O^b^	100	16	—	13	53	4	—
2	Pd(OAc)_2_	DavePhos	PhMe/H_2_O^b^	90	24	—	9	58	3	—
3	PdCl_2_(PPh_3_)_2_	DPPF	PhMe/H_2_O^b^	100	15	—	—	36	—	—
4	PdCl_2_(PPh_3_)_2_	DavePhos	Dioxane/H_2_O^c^	100	15	—	3	47	3	2
5	Pd(OAc)_2_	DavePhos	THF/H_2_O^d^	70	15	10	—	56	—	12
6	PdCl_2_(PPh_3_)_2_	DavePhos	PhMe/H_2_O^b^	100	15	—	4	85	4	—
7	PdCl_2_(PPh_3_)_2_	^*t*^BuPhos	PhMe/H_2_O^b^	100	15	4	3	68	4	—
8	PdCl_2_(PPh_3_)_2_	DavePhos	PhMe/H_2_O^b^^,^^e^	100	0.7	—	5	69	6	—
**9**	**PdCl**_**2**_ **(PPh**_**3**_ **)**_**2**_	**DavePhos**	**THF/H**_**2**_ **O** d ^**,**^ f	**80**	**24**	**—**	**—**	**95**	**—**	**—**

a The experiments were carried out at a final substrate concentration of 0.1 M in the mixture of solvents, employing 1.6 equiv. of the trifluoroborate salt, 3 equiv. Cs_2_CO_3_, 5 mol% of the Pd catalyst and 10 mol% of the ligand.

b A PhMe/H_2_O 4 : 1 (v/v) medium was employed.

c The reaction was carried in a dioxane/H_2_O 10 : 1 (v/v) medium.

d A THF/H_2_O 4 : 1 (v/v) medium was used.

e The reaction was carried out under microwave irradiation (250 W).

f LiCl (0.4 equiv.) was added.

Furthermore, under these conditions, the required product was observed in mixture with minor amounts of the *Z*-isomer 22 (3–4%) and with the allyl derivative 19 (9–13%). This was somewhat surprising, since alkenyltrifluoroborates are known to retain the double bond geometry with a high degree of fidelity.^24*b*^

Changing the palladium source to PdCl_2_(PPh_3_)_2_ did not met with much better success (entries 3 and 4) when DPPF was employed as ligand (36% yield) or in the presence of DavePhos in dioxane/H_2_O (10 : 1, v/v). Similar moderate results (53–56% yield) were obtained with the use of Pd(OAc)_2_ and DavePhos in THF/H_2_O (4 : 1, v/v) at 70 °C for 15 h (entry 5), where some unreacted starting material (10%) was concomitantly isolated, along with the proto-deoxygenated product 23 (12%), recognized through the diagnostic signals in its ^1^H NMR spectrum [*δ* 7.55 (dd, *J* = 1.5 and 7.8, H-2), 7.50 (d, *J* = 1.5, H-6) and 7.03 (d, *J* = 7.8, H-3)].

Employing a toluene/water solvent in association with PdCl_2_(PPh_3_)_2_ was found to afford significantly improved results, attaining 85% yield of 20 (entry 6). This performance could not be surpassed by the use of ^*t*^BuPhos as ligand (entry 7) nor by microwave irradiation (entry 8), which gave 20 in 68–69% yield. In all these cases, mixtures of 19, 20 and 22 were observed.

Examining the whole set of results, it was noticed that the deoxygenated product 23 was obtained only in ethereal solvents. It was conjectured that this outcome may be the result of the lack of reactivity or stability of some reaction intermediate. Thus, it was thought that addition of LiCl may improve the results.

It has been shown that LiCl is a required additive when the transformation is run in ethereal solvents, because it may act as a source of chloride ligand, stabilizing the Pd intermediates and making the cross-coupling stage more efficient.^28^ It has also been demonstrated that the lithium salt turns the Pd catalyst more active towards transmetallation and increases its proneness to undergo oxidative addition, resulting in a powerful accelerant of the reaction rate. Furthermore, LiCl enhances both the polarity of the solvent, and the leaving ability of anionic ligands.

**Scheme 3:**
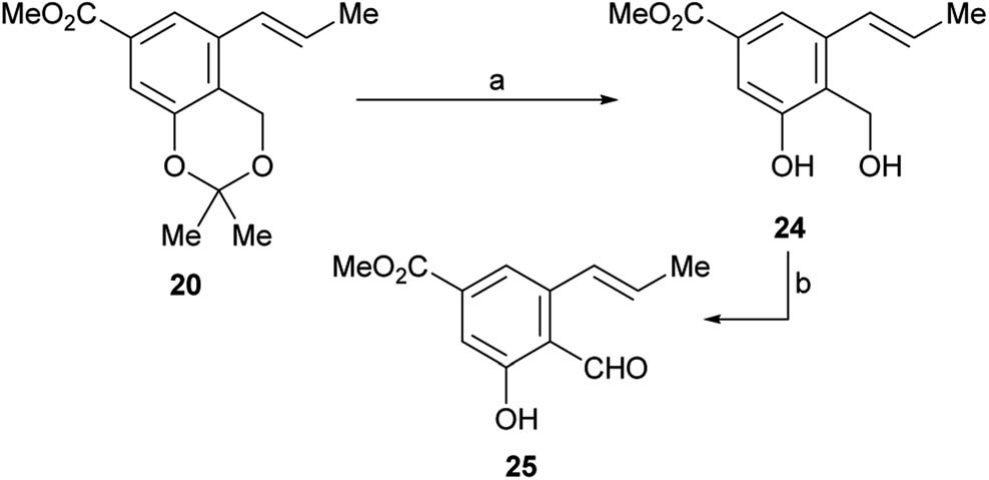
Reagents and conditions: (a) TsOH (cat.), THF/H_2_O, 60 °C, 48 h, (98%) or 1 M HCl, THF, 45 °C, 18 h, (90%); (b) see Table 2.

Therefore, LiCl was added to a THF/H_2_O mixture of the starting triflate, Cs_2_CO_3_, PdCl_2_(PPh_3_)_2_ and DavePhos. Rewardingly, the LiCl supplemented reaction cleanly afforded 95% yield of the expected derivative 20, free of isomers and under milder conditions (80 °C), as shown in entry 9. Next, the acetonide moiety of 20 was smoothly hydrolysed by exposure to *p*-toluenesulfonic acid in THF/H_2_O, affording the benzylic alcohol 24 in almost quantitative yield (Scheme 3).

However, oxidation of the latter to the corresponding aldehyde 25, which is a key step of the synthesis, proved to be tricky. The starting alcohol demonstrated to exhibit some instability, probably favoured by an easy benzylic protonation, followed by dehydration and further quinomethide formation; on the other hand, the tested oxidants were also able to oxidize the neighbouring phenol moiety to the related quinones.

As shown in Table 2, a series of trial and error experiments with PDC,^29^ IBX^30^ and activated MnO_2_ (ref. 31) were executed. After initial discouraging results (entries 1–3) where 25 was obtained (20–34% yield) along with unidentifiable products, presumable resulting from overoxidation,^32^ it was found that MnO_2_ provided a significantly better yield when the solvent was changed from CH_2_Cl_2_ to EtOAc (entry 4).

**Table 2:** Optimization of the oxidation of benzyl alcohol 24 to 25

Run no.	Oxidizing agent	Solvent	Temperature (°C)	Time (h)	Yield (%)
1	IBX	EtOAc	50	1.5	23
2	PDC	CH_2_Cl_2_	r.t.	2	34
3	MnO_2_	CH_2_Cl_2_	r.t.	16	20
4	MnO_2_	EtOAc	r.t.	16	50
5	MnO_2_	EtOAc	50	5	54
**6**	**MnO**_**2**_	**EtOAc**	**78**	**3**	**55**

Additionally, it was observed that warming to 50 °C improved the performance to 54% yield (entry 5), also shortening the reaction time from 16 h to 5 h, whereas the best results (55% yield) were achieved in boiling EtOAc for 3 h (entry 6), when it was observed complete consumption of the starting material.

**Scheme 4:**
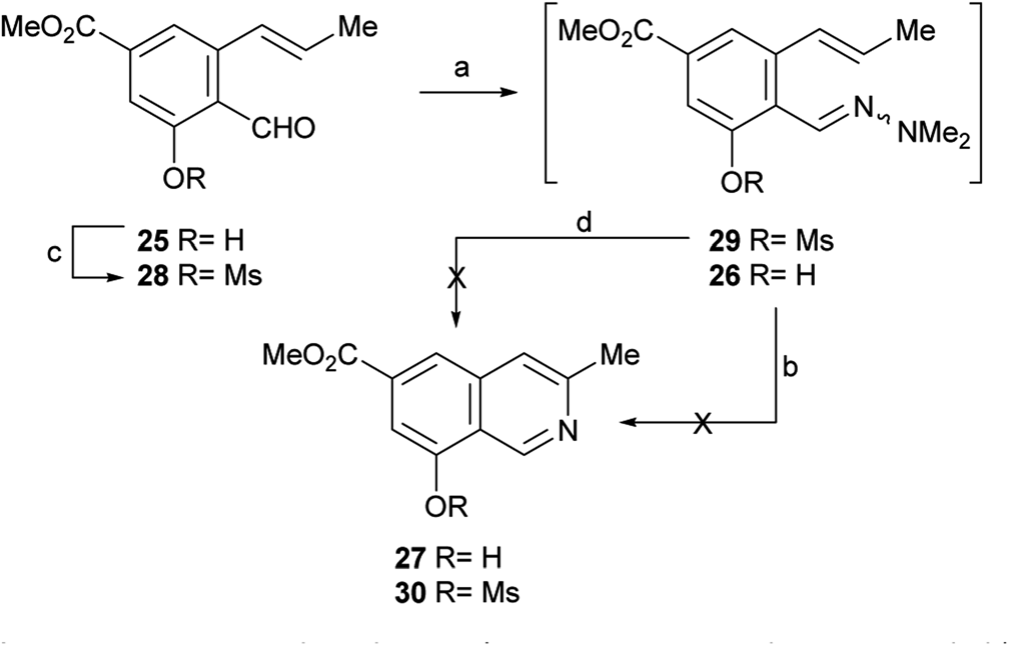
Reagents and conditions: (a) Me_2_N–NH_2_, AcOH, PhCF_3_, r.t., 1.5 h; (b) MW, 160 °C, 45 min (25, 50% recovered); (c) MsCl, Et_3_N, CH_2_Cl_2_, 0 °C→r.t., 24 h (55%); (d) MW, 160 °C, 45 min.

After having secured a satisfactory access to the key propenyl salicylaldehyde 25, the latter was submitted to our recently developed one-pot hydrazonation/6π-azaelectrocyclization protocol.^33^ Thus, 25 was exposed to 1,1-dimethylhydrazine in PhCF_3_ with the addition of AcOH as a promoter, to afford the hydrazone intermediate 26 (Scheme 4). Without purification, the hydrazone was heated at 160 °C for 45 min in the same medium, under microwave irradiation. Unexpectedly, however, the process resulted in recovery of 50% of the starting aldehyde 25, and no signs of the expected product 27 could be detected.

Under the hypothesis that the presence of a free phenolic hydroxyl group could have caused the observed outcome, 25 was protected with a readily removable group. Therefore, 25 was treated with MeSO_2_Cl and Et_3_N and DMAP in CH_2_Cl_2_, to afford 55% yield of the unstable mesylate ester 28. The latter exhibited diagnostic signals of the methanesulfonate group, as a singlet resonating at *δ*_H_ 3.32 ppm and a resonance at *δ*_C_ 38.6 ppm.

Unfortunately, however, application of the one-pot hydrazonation/6π-azaelectrocyclization protocol also met with failure; neither the expected mesylate 30 nor the isoquinoline 27 were found.^33^

**Scheme 5:**
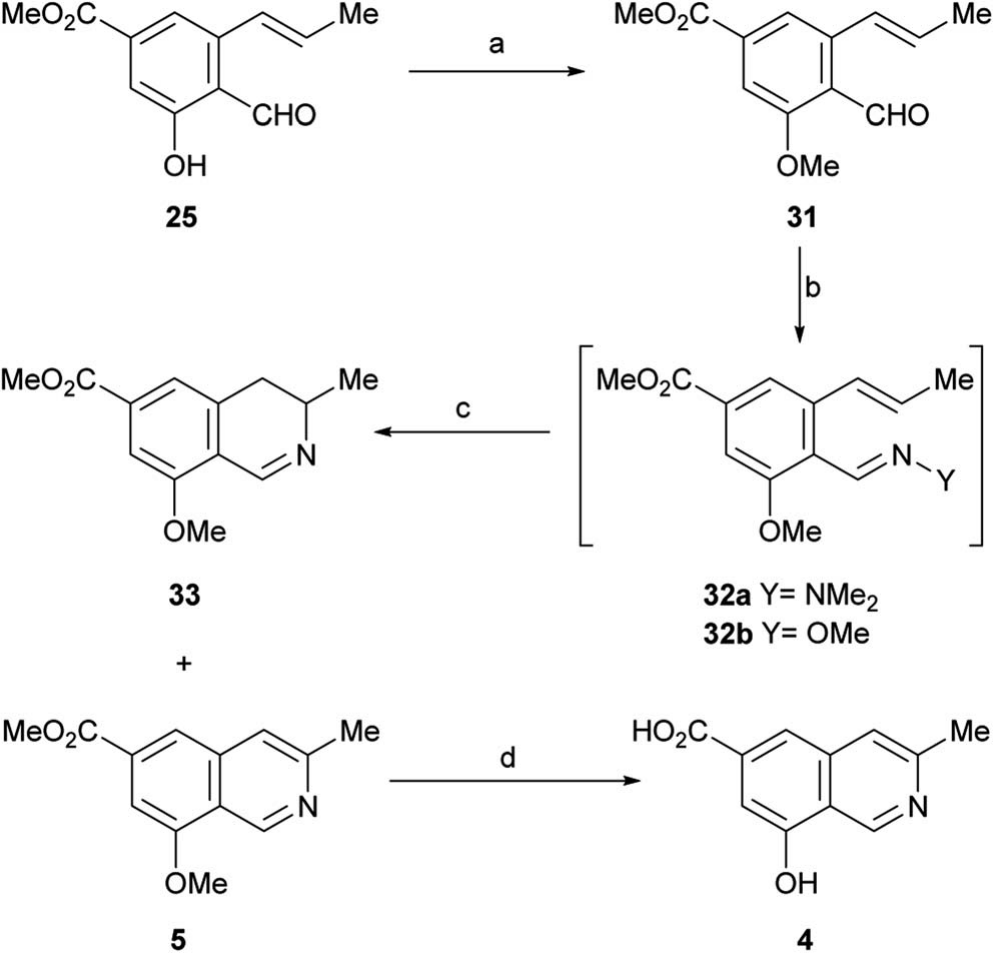
Reagents and conditions: (a) MeI, K_2_CO_3_, DMF, 0→t.a., 1.5 h (85%); (b) Me_2_N–NH_2_, AcOH, PhCF_3_, r.t., 1.5 h or MeO–NH_2_, EtOH, r.t., 1 h; (c) MW, 160 °C (*via*32a: 1 h, 5, 64%; 33, 26%; *via*32b; 45 min, 5, 50%; 33, 15%); (d) Al^0^, I_2_, DMSO, MeCN, 80 °C, 20 h (23%).

In view of these discouraging results, an alternate protecting group was sought. Considering our previous results with the benzyl ether and MOM as protecting groups,^33^ and taking into account our objective, to synthetically access permethylampullosine (5), the intermediate 25 was *O*-methylated with MeI in DMF at 0 °C, employing K_2_CO_3_ as base, to give 31 in 85% yield (Scheme 5). Then, the latter was submitted to our hydrazonation/6π-azaelectrocyclization/elimination protocol,^33^ affording 5 in 64% overall yield, through the intermediacy of 32a, which was not isolated because of its poor hydrolytic stability.

Interestingly, the 3,4-dihydroisoquinoline 33 was isolated concomitantly in 26% yield. The latter was recognized by its pattern of signals in the ^1^H NMR spectrum, related to H-3 (m), H-4 (dd) and Me-3 (d). A reaction mechanism to explain the generation of 3,4-dihydroisoquinolines as side products of 6π-azaelectrocyclization of similar heterocyclic systems has been proposed.^33^

It has been shown that, in aqueous solution, aliphatic hydrazones are 10^2^–10^3^ times more sensitive to hydrolysis than the analogous oximes.^34^ Therefore, conjecturing that the use of a more stable ammonia derivative for condensation with 31 may improve the performance of the 6π-azaelectrocyclization, 31 was converted into the methoxime intermediate 32b by reaction with methoxylamine in absolute EtOH. After replacing the solvent with PhCF_3_, compound 32b was heated under microwave irradiation at 180 °C for 45 min to undergo the 6π-azaelectrocyclization, furnishing 5 in 50% yield, accompanied by the related 3,4-dihydroisoquinoline 33 (15% yield).

With these results it was concluded that the hydrazone pathway should be preferred to the methoxime alternative. The ^1^H and ^13^C NMR spectroscopic data of the synthetic compound 5 fully matched those reported by the group of Arnold for their semisynthetic *O*-methyl ampullosine methyl ester.^9^

It was also found that treatment of 5 with aluminium and iodine (which generates *in situ* the powerful Lewis acid AlI_3_),^35^ effected the simultaneous removal of both methyl groups, affording ampullosine (4) in 23% yield.

## Experimental

### General information

All the reactions were carried out under dry argon atmospheres, using oven-dried glassware and freshly distilled anhydrous solvents. Anhydrous CH_2_Cl_2_ was obtained from an M. Braun solvent purification and dispenser system. Anhydrous EtOAc was obtained by a 3 h reflux of the PA grade product over P_2_O_5_ and further distillation of the liquid. Anhydrous DMF was obtained by heating the PA grade product over BaO for 4 h, followed by distillation under reduced pressure. Anhydrous Et_3_N was prepared by distillation of the commercial product from CaH_2_. The anhydrous solvents were stored in dry Young ampoules. All other solvents and reagents were used as received.

The reactions were monitored by TLC (silica gel 60 GF_254_) run in different hexanes:EtOAc mixtures. The chromatographic spots were revealed by exposure to UV light (254 and 365 nm) and spraying with ethanolic *p*-anisaldehyde/sulfuric acid reagent, followed by careful heating to improve selectivity; to detect the isoquinolines, the Dragendorff reagent (Munier and Macheboeuf modification)^36*a*^ was used. The flash column chromatographies were performed under conditions that entailed a modification of known procedures.^36*b*–*d*^ They were run under positive pressure with slurry-packed silica gel 60 H for thin layer chromatography (particle size < 55 μm), employing gradient of solvent polarity techniques with hexane : EtOAc, hexane : CH_2_Cl_2_ and EtOAc : EtOH, as required. The column size (height and diameter), amount of silica gel, solvent polarity variation (gradient) step, solvent volume per step and fraction volume were adjusted for each case (compound and reaction), taking into account mainly the amount of sample, its polarity and the presence of impurities, especially if their *R*_f_ value in the corresponding TLC analysis was close to that of the product. The chromatographic variables were adjusted aiming to elute the product approximately after the 15^th^ tube, when the solvent polarity was equivalent to that yielding a *R*_f_ ≈ 0.50 in the TLC plate. The chromatographies were continued after elution of the expected product, in order to ensure recovery of all the relevant compounds.

### Equipment

The melting points (uncorrected) were measured on an Ernst Leitz Wetzlar model 350 hot-stage microscope. The FT-IR spectra were recorded on a Shimadzu Prestige 21 spectrophotometer, as solid dispersions in KBr disks (solid samples). The NMR spectra were recorded in CDCl_3_ unless informed otherwise with a Bruker Avance 300 NMR spectrometer (300.13 MHz for ^1^H, 75.48 MHz for ^13^C and 282.38 MHz for ^19^F). In addition, some spectra were acquired with a Bruker Avance 400 MHz spectrometer (400.13 MHz for ^1^H, 100.61 MHz for ^13^C). The chemical shifts are reported in ppm on the *δ* scale, and TMS was used as the internal standard (*δ* = 0.0 ppm); the residual solvent peaks of CDCl_3_ (*δ*_H_ = 7.26 ppm, *δ*_C_ = 77.16 ppm), DMSO-*d*_*6*_ (*δ*_H_ = 2.50 ppm, *δ*_C_ = 39.52 ppm) and C*D*_3_OD (*δ*_H_ = 3.31 ppm, *δ*_C_ = 49.00 ppm) as well as the signal of CFCl_3_ for (*δ*_F_ = 0.00 ppm), were used as internal reference. The magnitudes of the coupling constant (*J*) values are given in hertz. To complete the elucidation, NOE and 2D NMR experiments (HSQC and HMBC) were employed. HRMS was obtained with a Bruker MicroTOF-Q II instrument from UMyMFOR (Buenos Aires, Argentina). The microwave-assisted reactions were carried out in a CEM Discover microwave reactor. The UV-Vis spectrum of ampullosine (4) was acquired in an Agilent model 8453 spectrophotometer equipped with a diode-array detector, in the range 205–700 nm and matched quartz cells (10 mm optical path). The sample was dissolved in MeOH, and the spectrum was taken against a blank of the solvent.

#### Dimethyl 2,6-dihydroxyterephthalate (16)

A mixture of 3,5-dihydroxybenzoic acid (14) (380 mg, 2.5 mmol) in 1,2-propanediol (700 μL) was heated at 100 °C in a pressure tube until the mixture was completely homogenized. After cooling, KHCO_3_ (1.25 g, 12.5 mmol) was added, and the resulting suspension was purged with CO_2_, sealed and heated at 180 °C for 6 h. After cooling, water (50 mL) and concentrated HCl (50 mL) were added to the resulting whitish residue, the mixture was extracted with Et_2_O (6 × 50 mL). The combined extracts were dried over MgSO_4_ and the solvent was evaporated *in vacuo* on a rotary evaporator, affording the product dicarboxylic acid product 15 (381 mg, 80%) as a pale yellow solid. Without further purification, this solid was dissolved in acetone (5 mL), KHCO_3_ (500 mg, 5 mmol) was added and the mixture was stirred for 15 min at room temperature under an argon atmosphere. Then, Me_2_SO_4_ (470 μL, 5 mmol) was added dropwise, and the suspension was heated at reflux for 10 h. After completion of the reaction, the volatiles were evaporated, saturated NaHCO_3_ (50 mL) was added to the residue and the products were extracted with EtOAc (3 × 45 mL). The combined organic phases were dried over MgSO_4_, filtered, and concentrated under reduced pressure. The residue was purified by column chromatography (hexane:EtOAc, 1 : 0 → 2 : 8 v/v) to afford the desired product 16 (394 mg, 70%, two steps), as a white solid, *R*_f_: 0.53 (hexane : EtOAc, 6 : 4 v/v), mp: 146–148 °C (lit. 20*a* and 20*d* 146–149 °C); ^1^H NMR *δ*: 9.67 (br s, *w*_1/2_ = 10.2, 2H, ArO*H*), 7.11 (s, 2H, H-3 and H-5), 4.11 (s, 3H, 1-CO_2_*Me*) and 3.90 (s, 3H, 4-CO_2_*Me*); ^13^C NMR *δ*: 169.6 (1-*C*O_2_Me), 165.8 (4-*C*O_2_Me), 160.8 (C-2 and C-6), 137.3 (C-4), 109.2 (C-3 and C-5), 102.9 (C-1), 53.4 (1-CO_2_*Me*) and 52.6 (4-CO_2_*Me*).

#### Methyl 5-hydroxy-2,2-dimethyl-4*H*-benzo[*d*][1,3]dioxine-7-carboxylate (17)^20*a*,20*d*^

A mixture of NaBH_4_ (195 mg, 5.16 mmol) in 0.05 M phosphate buffer, pH 7.5 (1.5 mL) was added dropwise to a stirred solution of 16 (390 mg, 1.72 mmol) in THF (9 mL) cooled to 0 °C in an ice-water bath. The resulting solution was stirred for 1.5 h, when the reaction was quenched with 10% citric acid solution (5 mL) at 0 °C. Then, brine (20 mL) was added and the organic products were extracted with EtOAc (3 × 20 mL). The combined organic layers were dried over MgSO_4_ and concentrated *in vacuo*. The crude residue was purified by column chromatography to give methyl 3,5-dihydroxy-4-(hydroxymethyl)benzoate (286 mg), as a whitish solid. The product was filtered through a short silica gel column and without further purification, this solid was dissolved in 2,2-dimethoxypropane (5 mL), *p*-TsOH·H_2_O (32 mg, 0.17 mmol) was added at 0 °C, the ice bath was removed, and the mixture was stirred for 5 h at room temperature. The reaction mixture was placed again in an ice bath and NaHCO_3_ (20 mg) was added. After stirring for 5 min, the resulting solution was purified by silica gel column chromatography (hexane : EtOAc, 1 : 0 → 1 : 1 v/v), furnishing 17 (307 mg, 75%, two steps), as a white solid, *R*_f_: 0.50 (hexane : EtOAc 7 : 3 v/v), mp: 141–142 °C; IR (KBr, *ν̂*): 3327, 2995, 2846, 1697, 1595, 1431, 1365, 1276, 1149, 1060, 950, 862, 763 and 642 cm^−1^; ^1^H NMR *δ*: 7.15 (d, *J* = 1.2, 1H, H-8), 7.11 (d, *J* = 1.2, 1H, H-6), 5.92 (s, 1H, ArO*H*), 4.86 (s, 2H, H-4), 3.89 (s, 3H, CO_2_*Me*) and 1.55 (s, 6H, *Me*-2); ^13^C NMR *δ*: 167.2 (*C*O_2_Me), 152.5 (C-8a), 152.1 (C-5), 130.0 (C-7), 112.7 (C-4a), 111.0 (C-6), 107.7 (C-8), 99.8 (C-2), 58.1 (C-4), 52.5 (CO_2_*Me*) and 24.7 (*Me*-2); HRMS (ESI-TOF): found *m*/*z*: 261.0729; C_12_H_14_NaO_5_ ([M + Na]^+^) requires *m*/*z*: 261.0733.

#### Methyl 2,2-dimethyl-5-{[(trifluoromethyl)sulfonyl]oxy}-4*H*-benzo[*d*][1,3]dioxine-7-carboxylate (18)

A stirred suspension of phenol 27 (303 mg, 1.27 mmol) in anhydrous CH_2_Cl_2_ (4 mL) was cooled to 0 °C under argon and successively treated with DMAP (152 mg, 0.12 mmol), Et_3_N (322 mg, 445 μL, 3.18 mmol) and PhNTf_2_ (635 mg, 1.77 mmol). The reaction mixture was left to acquire room temperature and further stirred overnight. Then, the reaction was diluted with CH_2_Cl_2_ (50 mL), the organic phase was washed with saturated NaHCO_3_ (2 × 50 mL), dried over MgSO_4_, filtered, and concentrated under reduced pressure. The residue was chromatographed (hexane : CH_2_Cl_2_, 1 : 0 → 3 : 2 v/v), furnishing 18 (451 mg, 96%), as a white solid, *R*_f_: 0.20 (hexane : CH_2_Cl_2_, 7 : 3 v/v), mp: 63–64 °C; IR (KBr, *ν̂*): 3086, 2997, 2962, 1724, 1629, 1583, 1423, 1328, 1253, 1138, 1029, 925, 829, 767 and 638 cm^−1^; ^1^H NMR *δ*: 7.55 (d, *J* = 1.4, 1H, H-6), 7.51 (d, *J* = 1.4, 1H, H-8), 4.93 (s, 2H, H-4), 3.92 (s, 3H, CO_2_*Me*) and 1.56 (s, 6H, *Me*-2); ^13^C NMR *δ*: 165.1 (*C*O_2_Me), 153.1 (C-8a), 145.1 (C-5), 131.3 (C-7), 118.7 (q, *J* = 320, *C*F_3_), 118.6 (C-6), 118.0 (C-4a), 113.7 (C-8), 100.8 (C-2), 57.4 (C-4), 52.8 (CO_2_*Me*) and 24.6 (*Me*-2); ^19^F NMR *δ*: −73.3 (s, C*F*_3_SO_3_Ar); HRMS (ESI-TOF): found *m*/*z*: 393.0226; C_13_H_13_F_3_NaO_7_S ([M + Na]^+^) requires *m*/*z*: 393.0226.

#### Methyl 5-allyl-2,2-dimethyl-4*H*-benzo[*d*][1,3]dioxine-7-carboxylate (19)

A mixture of triflate 28 (29 mg, 0.08 mmol), LiCl (3 mg, 0.07 mmol), Ph_3_P (10 mg, 50 mol%), and PdCl_2_(PPh_3_)_2_ (6 mg, 10 mol%), was placed in a tube, capped with a septum, purged with dry argon for 5 minutes and then diluted with anhydrous diglyme (0.5 mL). After stirring for 5 min, the resulting mixture was treated with Bu_3_SnCH_2_CH=CH_2_ (31 μL, 0.10 mmol), the reaction tube was sealed and the mixture was heated at 125 °C for 15 h. After cooling to room temperature, the reaction mixture was diluted with Et_2_O (15 mL), a saturated solution of KF (10 mL) was added and the mixture was stirred for 20 min in order to quench the organotin-derivatives. The aqueous phase was separated and the organic layer was washed with brine (3 × 8 mL). The organic solvent was filtered through a short pad of Florisil and Celite (1 : 1 w/w) and the filtrate was dried over MgSO_4_ prior to concentration under reduced pressure. Column chromatography (hexane : EtOAc, 1 : 0 → 8 : 2 v/v) of the residue gave the corresponding allyl substituted compound 19 as a 95 : 5 mixture with the related β-propenyl derivative *E*-20 (14.6 mg, 70%), as a colour5 0.47 (hexane : EtOAc, 9 : 1 v/v); ^1^H NMR *δ*: 7.43 (d, *J* = 1.4, 1H, H-6), 7.39 (d, *J* = 1.4, 1H, H-8), 5.90 (ddt, *J* = 17.0, 10.1 and 6.2, 1H, ArCH_2_C*H*=CH_2_), 5.10 (ddt, *J* = 10.1, 1.4 and 1.4, 1H, ArCH_2_CH=C*H*_2*cis*_), 5.00 (ddt, *J* = 17.0, 1.6 and 1.6, 1H, ArCH_2_CH=C*H*_2*trans*_), 4.83 (s, 2H, H-4), 3.88 (s, 3H, CO_2_*Me*), 3.25 (dt, *J* = 6.2 and 1.6, 2H, ArC*H*_2_CH=CH_2_) and 1.52 (s, 6H, *Me*-2); ^13^C NMR *δ*: 166.9 (*C*O_2_Me), 151.5 (C-8a), 136.2 (C-5), 135.1 (ArCH_2_*C*H=CH_2_), 130.0 (C-7), 123.2 (C-4a), 122.3 (C-6), 116.8 (ArCH_2_CH=*C*H_2_), 116.7 (C-8), 99.3 (C-2), 59.7 (C-4), 52.2 (CO_2_*Me*), 36.2 (Ar*C*H_2_CH=CH_2_) and 24.7 (*Me*-2).

#### Methyl (*E*)-2,2-dimethyl-5-(propen-1-yl)-4*H*-benzo[*d*][1,3]dioxine-7-carboxylate (20)

A mixture of the triflate 18 (100 mg, 0.27 mmol), potassium *trans*-1-propenyltrifluoroborate (68 mg, 0.45 mmol), Cs_2_CO_3_ (264 mg, 0.81 mmol), LiCl (5 mg, 0.12 mmol), DavePhos (10 mg, 10 mol%), and PdCl_2_(PPh_3_)_2_ (9 mg, 5 mol%), was placed in a pressure tube capped with septum. The solids were treated with THF (2 mL) and degassed water (0.5 mL) with stirring and then were purged with dry argon for 5 minutes. The tube was sealed and the reaction mixture was heated at 80 °C for 24 h in an oil bath. After cooling to room temperature, the reaction mixture was diluted with water (6 mL) and extracted with CH_2_Cl_2_ (3 × 10 mL). The combined organic phases were dried over MgSO_4_, filtered, and concentrated under reduced pressure. The residue was purified by column chromatography (hexane : EtOAc, 1 : 0 → 8 : 2 v/v), to afford the desired product 20 (67 mg, 95%), as a white solid, *R*_f_: 0.47 (hexane : EtOAc, 9 : 1 v/v), mp: 70–71 °C; IR (KBr, *ν̂*): 3030, 2999, 2850, 1722, 1577, 1431, 1338, 1224, 1141, 1012, 904, 819 and 767 cm^−1^; ^1^H NMR *δ*: 7.67 (d, *J* = 1.4, 1H, H-6), 7.36 (d, *J* = 1.4, 1H, H-8), 6.20–6.33 (m, 2H, ArC*H*=CHMe and ArCH=C*H*Me), 4.88 (s, 2H, H-4), 3.89 (s, 3H, CO_2_*Me*), 1.91 (d, *J* = 4.7, 3H, ArCH=CH*Me*) and 1.53 (s, 6H, *Me*-2); ^13^C NMR *δ*: 167.0 (*C*O_2_Me), 151.4 (C-8a), 135.2 (C-5), 130.2 (Ar*C*H=CHMe), 130.0 (C-7), 125.6 (ArCH=*C*HMe), 121.2 (C-4a), 118.8 (C-8), 116.6 (C-6), 99.2 (C-2), 60.2 (C-4), 52.2 (CO_2_*Me*), 24.7 (*Me*-2) and 18.9 (ArCH=CH*Me*); HRMS (ESI-TOF): found *m*/*z*: 285.1098; C_15_H_18_NaO_4_ ([M + Na]^+^) requires *m*/*z*: 285.1097.

#### Methyl (*E*)-3-hydroxy-4-(hydroxymethyl)-5-(propen-1-yl) benzoate (24)

##### Method A

A stirred solution of the acetonide 20 (187 mg, 0.71 mmol) and *p*-TsOH.H_2_O (26 mg, 0.14 mmol) in a THF : H_2_O mixture (1 : 1 v/v, 14 mL) was heated at 60 °C for 48 h. The reaction was cooled to 0 °C, quenched with saturated aqueous NaHCO_3_ (30 mL), and the resulting mixture was extracted with EtOAc (3 × 50 mL). The combined organic layers were dried over MgSO_4_, and concentrated *in vacuo*. The crude residue was purified by column chromatography (hexane : EtOAc, 1 : 0 → 4 : 6 v/v), to give 24 (154 mg, 98%), as a white solid, *R*_f_: 0.25 (hexane : EtOAc, 7 : 3 v/v), mp: 95–97 °C; IR (KBr, *ν̂*): 3502, 3385, 3169, 2953, 2850, 1689, 1581, 1427, 1340, 1247, 1099, 1001, 964, 883 and 761 cm^−1^; ^1^H NMR *δ*: 8.12 (br s, 1H, *W*_1/2_ = 15.3 Hz, ArO*H*), 7.54 (d, *J* = 1.6, 1H, H-6), 7.35 (d, *J* = 1.6, 1H, H-2), 6.48 (dq, *J* = 15.5 and 1.7, 1H, ArC*H*=CHMe), 6.08 (dq, *J* = 15.5 and 6.6, 1H, ArCH=C*H*Me), 4.96 (s, 2H, ArC*H*_*2*_OH), 3.88 (s, 3H, CO_2_*Me*), 3.04 (br s, 1H, *W*_1/2_ = 21.8 Hz, ArCH_2_O*H*) and 1.87 (dd, *J* = 6.6 and 1.7, 3H, ArCH=CH*Me*); ^13^C NMR *δ*: 167.3 (*C*O_2_Me), 156.4 (C-3), 138.2 (C-5), 130.7 (ArCH=*C*HMe), 130.3 (C-4), 127.2 (Ar*C*H=CHMe), 126.5 (C-1), 120.0 (C-6), 115.8 (C-2), 60.1 (Ar*C*H_2_OH), 52.4 (CO_2_*Me*) and 18.8 (ArCH=CH*Me*); HRMS (ESI-TOF): found *m*/*z*: 245.0782; C_12_H_14_NaO_4_ ([M + Na]^+^) requires *m*/*z*: 245.0784.

##### Method B

A stirred solution of 20 (98 mg, 0.37 mmol) in THF (4.5 mL) was treated with 1 M HCl (3 mL) and the system was heated at 45 °C for 18 h. The reaction was cooled to 0 °C and quenched with saturated NaHCO_3_ (10 mL); the organic products were extracted with EtOAc (3 × 15 mL). The combined organic layers were dried over MgSO_4_, and concentrated *in vacuo*. The crude residue was purified by column chromatography to give 24 (74 mg, 90%). The spectroscopic data of the product were in full agreement with those of the product synthesized according to Method A.

#### Methyl (*E*)-4-formyl-3-hydroxy-5-(propen-1-yl)benzoate (25)

A mixture of the benzylic alcohol 24 (50 mg, 0.22 mmol) and freshly activated MnO_2_ (287 mg, 3.30 mmol) was slurred in anhydrous EtOAc (3 mL) and the system was heated to reflux for 3 h. After cooling, the reaction mixture was filtered through a Celite pad, and the filtrate was concentrated *in vacuo*. The resulting yellow residue was purified by column chromatography (hexane : EtOAc, 1 : 0 → 7 : 3 v/v), to afford 25 (27 mg, 55%), as a yellow solid, *R*_f_: 0.50 (hexane : EtOAc, 8 : 2 v/v), mp: 140–141 °C; IR (KBr, *ν̂*): 3043, 2990, 2848, 1716, 1647, 1558, 1417, 1348, 1247, 1174, 1097, 999, 887 and 766 cm^−1^; ^1^H NMR *δ*: 11.74 (s, 1H, ArO*H*), 10.36 (s, 1H, C*H*O), 7.53 (d, *J* = 1.4, 1H, H-6), 7.46 (d, *J* = 1.4, 1H, H-2), 6.88 (dq, *J* = 15.5 and 1.7, 1H, ArC*H*=CHMe), 6.21 (dq, *J* = 15.5 and 6.6, 1H, ArCH=C*H*Me), 3.93 (s, 3H, CO_2_*Me*) and 1.97 (dd, *J* = 6.6 and 1.7, 3H, ArCH=CH*Me*); ^13^C NMR *δ*: 195.8 (*C*HO), 165.9 (*C*O_2_Me), 162.5 (C-3), 143.8 (C-5), 137.3 (C-1), 135.0 (ArCH=*C*HMe), 125.2 (Ar*C*H=CHMe), 119.5 (C-4), 119.4 (C-6), 117.4 (C-2), 52.7 (CO_2_*Me*) and 19.1 (ArCH=CH*Me*); HRMS (ESI-TOF): found *m*/*z*: 243.0633; C_12_H_12_NaO_4_ ([M + Na]^+^) requires *m*/*z*: 243.0628.

#### Methyl (*E*)-4-formyl-3-((methylsulfonyl)oxy)-5-(prop-1-en-1-yl) benzoate (28)

A stirred solution of 25 (35 mg, 0.16 mmol) in CH_2_Cl_2_ (1 mL) was cooled in an ice-water bath and successively treated with treated with Et_3_N (34 μL, 0.24 mmol) and MeSO_2_Cl (15 μL, 0.19 mmol). The reaction mixture was stirred at room temperature for 16 h. Then, brine (5 mL) was added to the reaction mixture and extracted with EtOAc (3 × 20 mL). The combined organic phases were washed successively with 0.2 M HCl (10 mL), saturated NaHCO_3_ (10 mL) and then dried over anhydrous MgSO_4_. The solvent was removed under reduced pressure and the residue was chromatographed (hexane : EtOAc, 1 : 0 → 2 : 8 v/v) to afford 28 (27 mg, 55%; 77% br s m), as an unstable colorless oil *R*_f_: 0.56 (hexane : EtOAc, 1 : 1 v/v); ^1^H NMR *δ*: 10.43 (s, C*H*O), 8.13 (d, *J* = 1.4, 1H, H-2), 7.88 (d, *J* = 1.4, 1H, H-6), 7.06 (dd, *J* = 15.6 and 1.7, 1H, ArC*H*=CHMe), 6.30 (dq, *J* = 15.6 and 6.6, 1H, ArCH=C*H*Me), 3.96 (s, 3H, CO_2_*Me*), 3.32 (s, 3H, MeSO_3_^−^) and 1.93 (dd, *J* = 6.6 and 1.7, 3H, ArCH=CH*Me*); ^13^C NMR *δ*: 189.7 (*C*HO), 164.9 (*C*O_2_Me), 149.3 (C-3), 142.5 (C-5), 134.8 (C-1), 134.4 (ArCH=*C*HMe), 129.0 (Ar*C*H=CHMe), 127.6 (C-4), 126.6 (C-6), 122.1 (C-2), 52.8 (CO_2_*Me*), 38.6 (MeSO_3_^−^) and 18.9 (ArCH=CH*Me*).

#### Methyl (*E*)-4-formyl-3-methoxy-5-(propen-1-yl)benzoate (31)

A mixture of the methyl 3-hydroxybenzoate 25 (53 mg, 0.24 mmol) and K_2_CO_3_ (46 mg, 0.33 mmol) in anhydrous DMF (1 mL) was stirred for 15 min at 0 °C. The system was treated with iodomethane (20 μL, 0.31 mmol) and the resulting reaction mixture was stirred at room temperature for 1.5 h. Then, the mixture was poured into cold water (10 mL), and the products were extracted EtOAc (3 × 15 mL). The combined organic phases were washed with brine (25 mL), dried over MgSO_4_, filtered and concentrated *in vacuo*. The residue was purified by column chromatography (hexane : EtOAc, 1 : 0 → 7 : 3 v/v) to afford 31 (48 mg, 85%), as a yellowish-white solid *R*_f_: 0.52 (hexane : EtOAc, 8 : 2, v/v), mp: 72–73 °C; IR (KBr, *ν̂*): 3086, 2954, 2873, 2785, 1718, 1681, 1566, 1404, 1330, 1238, 1103, 1010, 964, 819 and 765 cm^−1^; ^1^H NMR *δ*: 10.61 (d, *J* = 0.5, C*H*O), 7.77 (ddd, *J* = 1.4, 0.7 and 0.7, 1H, H-6), 7.48 (d, *J* = 1.4, 1H, H-2), 7.20 (dq, *J* = 15.5 and 1.7, 1H, ArC*H*=CHMe), 6.30 (dq, *J* = 15.5 and 6.6, 1H, ArCH=C*H*Me), 3.95 (s, 3H, ArO*Me*), 3.95 (s, 3H, CO_2_*Me*) and 1.93 (dd, *J* = 6.6 and 1.7, 3H, ArCH=CH*Me*); ^13^C NMR *δ*: 192.3 (*C*HO), 166.3 (*C*O_2_Me), 162.3 (C-3), 141.2 (C-5), 134.8 (C-1), 131.7 (ArCH=*C*HMe), 128.5 (Ar*C*H=CHMe), 124.6 (C-4), 120.8 (C-6), 110.1 (C-2), 56.3 (ArO*Me*), 52.7 (CO_2_*Me*) and 18.9 (ArCH=CH*Me*); HRMS (ESI-TOF): found *m*/*z*: 235.0960; C_13_H_15_O_4_ ([M + H]^+^) requires *m*/*z*: 235.0965.

#### Methyl 8-methoxy-3-methylisoquinoline-6-carboxylate (permethyl ampullosine, 5)^9^ and methyl 8-methoxy-3-methyl-3,4-dihydroisoquinoline-6-carboxylate (33)

##### Method A

Methyl 4-formylbenzoate 31 (22 mg, 0.09 mmol), glacial AcOH (5 μL, 0.09 mmol) and 1,1-dimethylhydrazine (7 μL, 0.09 mmol) were added to a microwave tube and diluted with PhCF_3_ (1 mL). The mixture was purged with argon and stirred at room temperature until TLC analysis indicated complete consumption of the starting aldehyde (∼1.5 h). Then, the vessel was capped and irradiated in the microwave reactor (160 °C, *ca.* 250 W, 1 h). After cooling to room temperature, the solvent was recovered by careful distillation under atmospheric pressure, and the residue was purified by column chromatography (hexane/EtOAc, 1 : 0 → 0 : 1 v/v), to afford 5 (13.3 mg, 64%), as a yellowish solid, *R*_f_: 0.60 (hexane/EtOAc, 6 : 4 v/v), mp: 126–129 °C; IR (KBr, *ν̂*): 3068, 2947, 2848, 1716, 1633, 1570, 1433, 1354, 1238, 1122, 1035, 927, 825 and 762 cm^−1^; ^1^H NMR (300 MHz, CDCl_3_) *δ*: 9.56 (s, 1H, H-1), 8.03 (s, 1H, H-5), 7.50 (s, 1H, H-4), 7.37 (d, *J* = 1.2, 1H, H-7), 4.07 (s, 3H, ArO*Me*), 3.99 (s, 3H, CO_2_*Me*) and 2.71 (s, 3H, *Me*-3); ^13^C NMR (75 MHz, CDCl_3_) *δ*: 166.9 (*C*O_2_Me), 156.9 (C-8), 153.5 (C-3), 147.4 (C-1), 137.2 (C-4a), 132.2 (C-6), 121.2 (C-5), 120.6 (C-8a), 119.1 (C-4), 103.6 (C-7), 56.0 (ArO*Me*), 52.7 (CO_2_*Me*) and 24.3 (*Me*-3); ^1^H NMR (400 MHz, CD_3_OD) *δ*: 9.31 (s, 1H, H-1), 7.94 (s, 1H, H-5), 7.58 (s, 1H, H-4), 7.34 (s, 1H, H-7), 4.05 (s, 3H, ArO*Me*), 3.97 (s, 3H, CO_2_*Me*) and 2.63 (s, 3H, *Me*-3); ^13^C NMR (100 MHz, CD_3_OD) *δ*: 167.8 (*C*O_2_Me), 158.0 (C-8), 153.7 (C-3), 147.5 (C-1), 138.6 (C-4a), 134.0 (C-6), 121.8 (C-5), 121.5 (C-8a), 121.0 (C-4), 104.7 (C-7), 56.5 (ArO*Me*), 53.1 (CO_2_*Me*) and 23.5 (*Me*-3); HRMS (ESI-TOF): found *m*/*z*: 254.0798; C_13_H_13_NNaO_3_ ([M + Na]^+^) requires *m*/*z*: 254.0788. The ^1^H and ^13^C NMR spectral data of compound 5 were in agreement with the literature.^9^

Increasing solvent polarity afforded 33 (5.5 mg, 26%), as an amber solid, mp: 99–101 °C; IR (KBr, *ν̂*): 3005, 2951, 2837, 1724, 1618, 1570, 1446, 1319, 1222, 1112, 1001, 968, 864 and 767 cm^−1^; ^1^H NMR (300 MHz, CDCl_3_) *δ*: 8.72 (d, *J* = 2.6, 1H, H-1), 7.45 (s, 1H, H-5), 7.42 (s, 1H, H-7), 3.93 (s, 6H, ArO*Me* and CO_2_*Me*), 3.60–3.70 (m, 1H, H-3), 2.78 (dd, *J* = 16.2 and 5.6, 1H, H_*B*_-4), 2.49 (dd, *J* = 16.2 and 12.1, 1H, H_*B*_-4) and 1.39 (d, *J* = 6.9, 3H, *Me*-3); ^13^C NMR (75 MHz, CDCl_3_) *δ*: 166.6 (*C*O_2_Me), 156.9 (C-8), 154.1 (C-1), 138.3 (C-4a), 133.1 (C-6), 121.2 (C-7), 120.2 (C-8a), 110.6 (C-5), 55.9 (ArO*Me*), 52.5 (CO_2_*Me*), 52.0 (C-3), 32.5 (C-4) and 21.6 (*Me*-3); HRMS (ESI-TOF): found *m*/*z*: 256.0949; C_13_H_15_NNaO_3_ ([M + Na]^+^) requires *m*/*z*: 256.0944.

##### Method B

A mixture of *O*-methylhydroxylamine hydrochloride (16 mg, 0.19 mmol) and NaOAc (17 mg, 0.21 mmoL) in absolute EtOH (1.5 mL) was stirred at room temperature for 30 min under argon, when the resulting suspension was allowed to settle. An aliquot of the solution (1 mL) was transferred to a microwave tube containing the methyl 4-formylbenzoate 31 (23 mg, 0.1 mmol) and the resulting mixture was stirred at room temperature for 1 h under argon. Then, the solvent was removed under reduced pressure and the residue was dissolved in PhCF_3_ (1 mL). The tube was purged with Argon and the mixture was irradiated in the microwave reactor (180 °C, *ca.* 250 W, 45 min). After cooling to room temperature, the solvent was recovered by careful distillation under atmospheric pressure, and the residue was purified through a short column chromatography, to afford a separable mixture of 5 (11.5 mg, 50%) and 33 (3.5 mg, 15%). Their spectroscopic data were in full agreement with those of the corresponding products, synthesized employing Method A.

#### Ampullosine (4)

Iodine (91 mg, 0.36 mmol) was added in one portion to a stirred mixture of permethylampullosine 5 (20 mg, 0.08 mmol), small pieces of aluminium foil (12 mg, 0.44 mmol) and dry DMSO (20 μL, 0.28 mmol) in acetonitrile (1 mL). The mixture was further stirred at 80 °C for 20 h and monitored by TLC until the completion of conversion. After cooling to room temperature, the reaction was acidified to pH 3 with solution of citric acid (0.2 M, 5 mL) and was treated with Na_2_SO_3_ (15 mg) before extraction with EtOAc (5 × 10 mL). The organic phases were combined, and dried over MgSO_4_. After removal of organic solvents under reduced pressure, the residue was purified by column chromatography (EtOAc : EtOH, 1 : 0 → 2 : 8 v/v), furnishing 4 (3.7 mg, 23%), as a yellow solid, *R*_f_: 0.40 (EtOAc : EtOH, 1 : 1 v/v). UV-Vis (MeOH): 227 nm (log *ε* = 4.3) and 356 nm (log *ε* = 3.5). ^1^H NMR (300 MHz, DMSO-*d*_*6*_) *δ*: 10.88 (bs, 1H, OH), 9.37 (s, 1H, H-1), 7.85 (s, 1H, H-5), 7.64 (s, 1H, H-4), 7.45 (s, 1H, H-7), 3.35 (s, 1H, OH) and 2.59 (s, 3H, *Me*-3); ^13^C NMR (100 MHz, DMSO-*d*_*6*_) *δ* 168.3 (6-CO_2_H), 154.8 (C-8), 152.2 (C-3), 147.2 (C-1), 137.3 (2C, C-4a and C-6), 119.5 (C-8a), 119.1 (C-4), 118.3 (C-5), 109.2 (C-7) and 24.2 (3-Me). HRMS (ESI-TOF): found *m*/*z*: 204.0659; C_11_H_10_NO_3_ ([M + H]^+^) requires *m*/*z*: 204.0655; found *m*/*z*: 226.0471; C_11_H_9_NNaO_3_ ([M + Na]^+^) requires *m*/*z*: 226.0475. The UV-Vis, ^1^H and ^13^C NMR spectral data were in agreement with the literature.^9^

## Conclusion

We have developed a straightforward and convenient approach toward the first total synthesis of ampullosine, a structurally unique 6-carboxy-3-methylisoquinoline. The heterocycle is the first and only alkaloid isolated from the saproparasitic fungus *Sepedonium ampullosporum*. The synthesis of *O*-methyl ampullosine methyl ester, from a common intermediate, is also reported.

The synthesis of the natural product used 3,5-dihydroxybenzoic acid as starting material and its key steps included a Kolbe–Schmitt type carboxylation for introduction of C1 of the heterocyclic ring, a Molander cross-coupling with potassium propenyl trifluoroborate to install the remaining three carbon atoms, a carbonyl hydrazonation to add the heterocyclic nitrogen and a final 6π-azaelectrocyclization to build the isoquinoline system.

The total synthesis of ampullosine was achieved in 12 steps and 3.2% overall yield, whereas that of its permethyl derivative was accomplished in 14% overall yield, after 11 steps. Hydrolytic demethylation of *O*-methyl ampullosine methyl ester also afforded ampullosine. To the best of our knowledge, this is the first report of an electrocyclization reaction toward isoquinolines bearing a 6-carboxylic acid derivative.

## Conflicts of interest

There are no conflicts of interest to declare.

## Supplementary Material

RA-009-C9RA06839B-s001
