# Supplementary material for: First total synthesis of ampullosine, a unique isoquinoline alkaloid isolated from Sepedonium ampullosporum, and of the related permethylampullosine
Source: RSC Adv. 2019 Oct 16;9(57):33096–106. doi: 10.1039/c9ra06839b (PMC9073199; doi:10.1039/c9ra06839b)
Supplement: RA-009-C9RA06839B-s001 [file RA-009-C9RA06839B-s001.pdf]

Electronic Supplementary Material (ESI) for RSC Advances.
This journal is © The Royal Society of Chemistry 2019

Electronic Supplementary Information

First total synthesis of ampullosine, a unique isoquinoline alkaloid isolated from *Sepedonium ampullosporum*, and of the related permethylampullosine

Didier F. Vargas, Enrique L. Larghi* and Teodoro S. Kaufman*

Instituto de Química Rosario (IQUIR, CONICET-UNR), and Facultad de Ciencias Bioquímicas y Farmacéuticas, Universidad Nacional de Rosario, Suipacha 531, 2000 Rosario, Argentina

TABLE OF CONTENTS

Description of Content	Page
**Scheme S1.** The first total synthesis of ampullosine (**4**) and the synthesis of permethylampullosine (**5**)	S3
**Figure S1.** 300 MHz ^{1}H (top) and 75 MHz ^{13}C (bottom) NMR spectra of compound **17** in $CDCl_3$	S4
**Figure S2.** HSQC (top) and HMBC (bottom) spectra of compound **17** in $CDCl_3$	S5
**Figure S3.** High resolution mass spectrum of compound **17**	S6
**Figure S4.** 300 MHz ^{1}H (top) and 75 MHz ^{13}C (bottom) NMR spectra of compound **18** in $CDCl_3$	S7
**Figure S5.** HSQC (top) and HMBC (bottom) spectra of compound **18** in $CDCl_3$	S8
**Figure S6.** 282 MHz ^{19}F NMR spectrum of compound **18** in $CDCl_3$ (top) and HRMS of compound **18** (bottom)	S9
**Figure S7.** 300 MHz ^{1}H (top) and 75 MHz ^{13}C (bottom) NMR spectra of compound **19** in $CDCl_3$	S10
**Figure S8.** HSQC (top) and HMBC (bottom) spectra of compound **19** in $CDCl_3$	S11
**Figure S9.** High resolution mass spectrum of compound **19**	S12
**Figure S10.** 300 MHz ^{1}H (top) and 75 MHz ^{13}C (bottom) NMR spectra of compound **20** in $CDCl_3$	S13
**Figure S11.** HSQC (top) and HMBC (bottom) spectra of compound **20** in $CDCl_3$	S14
**Figure S12.** High resolution mass spectrum of compound **20**	S15
**Figure S13.** 300 MHz ^{1}H (top) and 75 MHz ^{13}C (bottom) NMR spectra of compound **24** in $CDCl_3$	S16
**Figure S14.** HSQC (top) and HMBC (bottom) spectra of compound **24** in $CDCl_3$	S17
**Figure S15.** High resolution mass spectrum of compound **21**	S18
**Figure S16.** 300 MHz ^{1}H (top) and 75 MHz ^{13}C (bottom) NMR spectra of compound **25** in $CDCl_3$	S19
**Figure S17.** HSQC (top) and HMBC (bottom) spectra of compound **25** in $CDCl_3$	S20
**Figure S18.** High resolution mass spectrum of compound **25**	S21
**Figure S19.** 300 MHz ^{1}H (top) and 75 MHz ^{13}C (bottom) NMR spectra of compound **31** in $CDCl_3$	S22
**Figure S20.** HSQC (top) and HMBC (bottom) spectra of compound **31** in $CDCl_3$	S23
**Figure S21.** High resolution mass spectrum of compound **31**	S24
**Figure S22.** 300 MHz ^{1}H (top) and 75 MHz ^{13}C (bottom) NMR spectra of compound **5** in $CDCl_3$	S25
**Figure S23.** HSQC (top) and HMBC (bottom) spectra of compound **5** in $CDCl_3$	S26
**Figure S24.** 400 MHz ^{1}H (top) and 100 MHz ^{13}C (bottom) NMR spectra of compound **5** in MeOD-d_4	S27
**Figure S25.** HSQC (top) and HMBC (bottom) spectra of compound **5** in MeOD-d_4	S28
**Figure S26.** High resolution mass spectrum of compound **5**	S29
**Figure S27.** 300 MHz ^{1}H (top) and 75 MHz ^{13}C (bottom) NMR spectra of compound **33** in $CDCl_3$	S30
**Figure S28.** HSQC (top) and HMBC (bottom) spectra of compound **33** in $CDCl_3$	S31
**Figure S29.** High resolution mass spectrum of compound **33**	S32
**Figure S30.** 300 MHz ^{1}H (top) and 75 MHz ^{13}C (bottom) NMR spectra of ampullosine (**4**) in DMSO-d_6	S33
**Figure S31.** HSQC (top) and HMBC (bottom) spectra of ampullosine (**4**) in DMSO-d_6	S34
**Figure S32.** High resolution mass spectrum of ampullosine (**4**)	S35
**Figure S33.** UV-Vis spectrum of ampullosine (**4**) in MeOH	S36
**Table S1.** Spectroscopic comparison of ^{1}H NMR data of permethylampullosine (**5**)	S37
**Table S2.** Spectroscopic comparison of ^{13}C NMR data of permethylampullosine (**5**)	S37
**Table S3.** Spectroscopic comparison of NMR data of ampullosine (**4**)	S38
**References**	S38

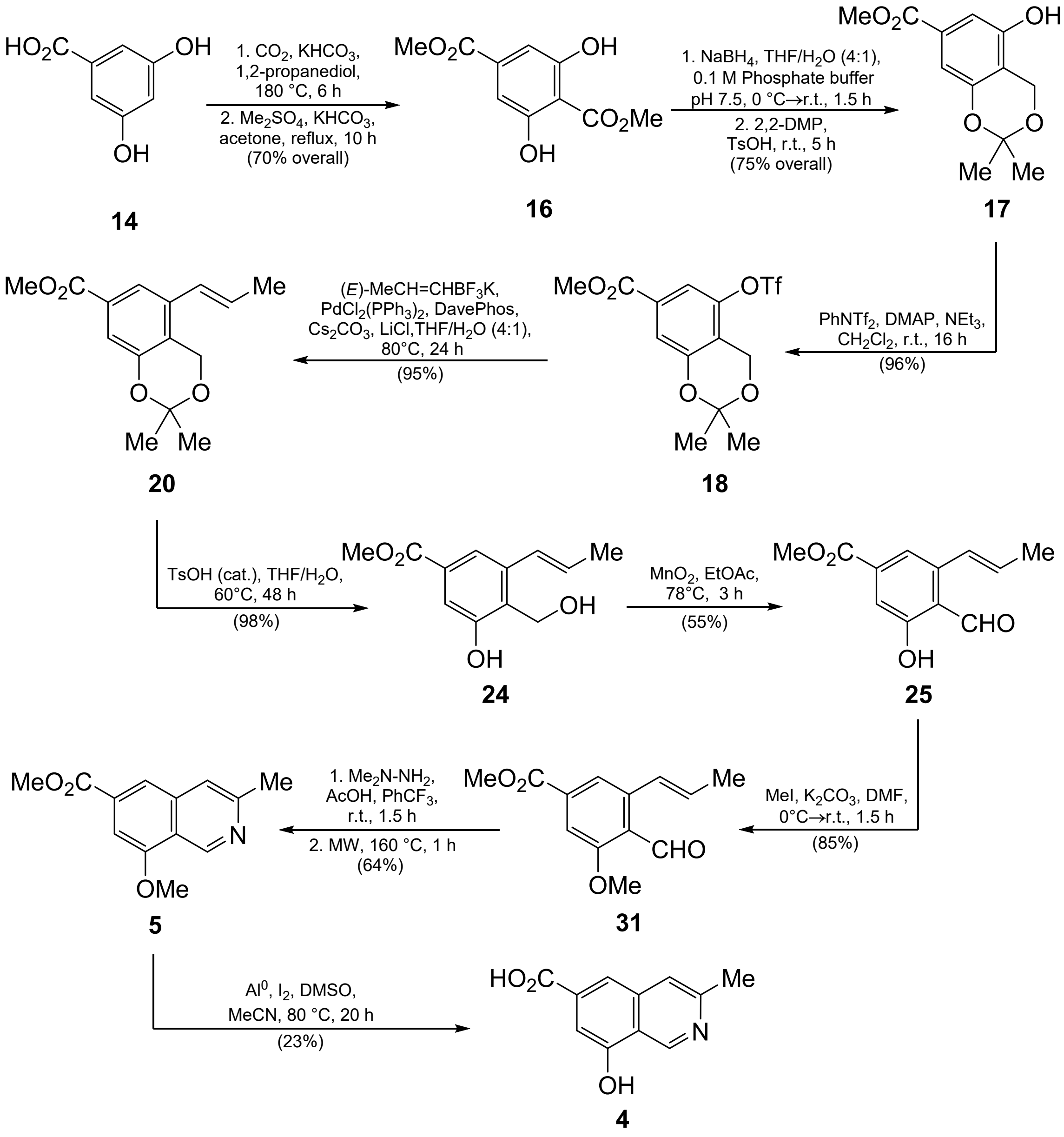

Scheme S1. The first total synthesis of ampullosine (**4**) and the synthesis of permethylampullosine (**5**).

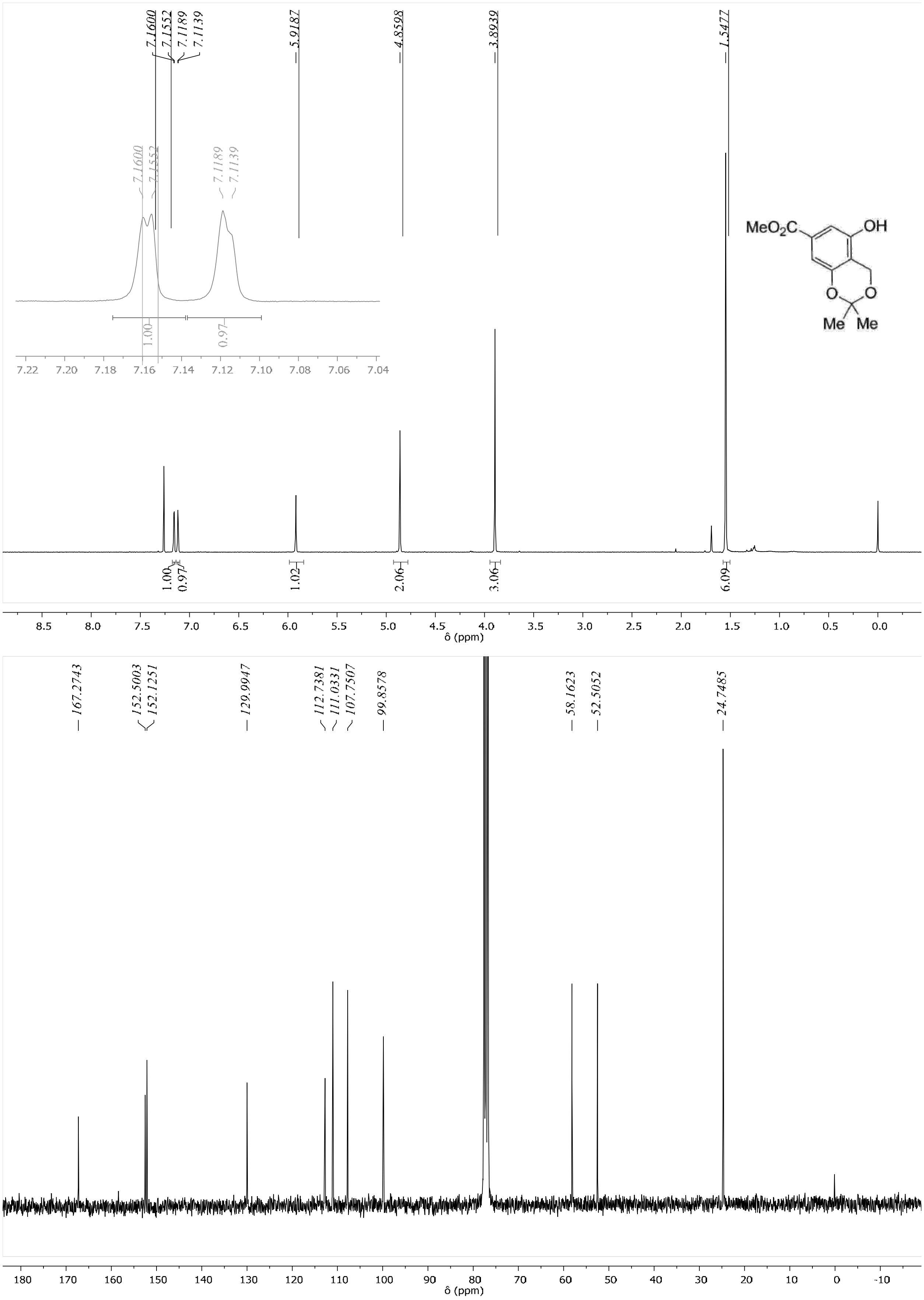

Figure S1. 300 MHz ^{1}H (top) and 75 MHz ^{13}C (bottom) NMR spectra of compound **17** in $CDCl_3$.

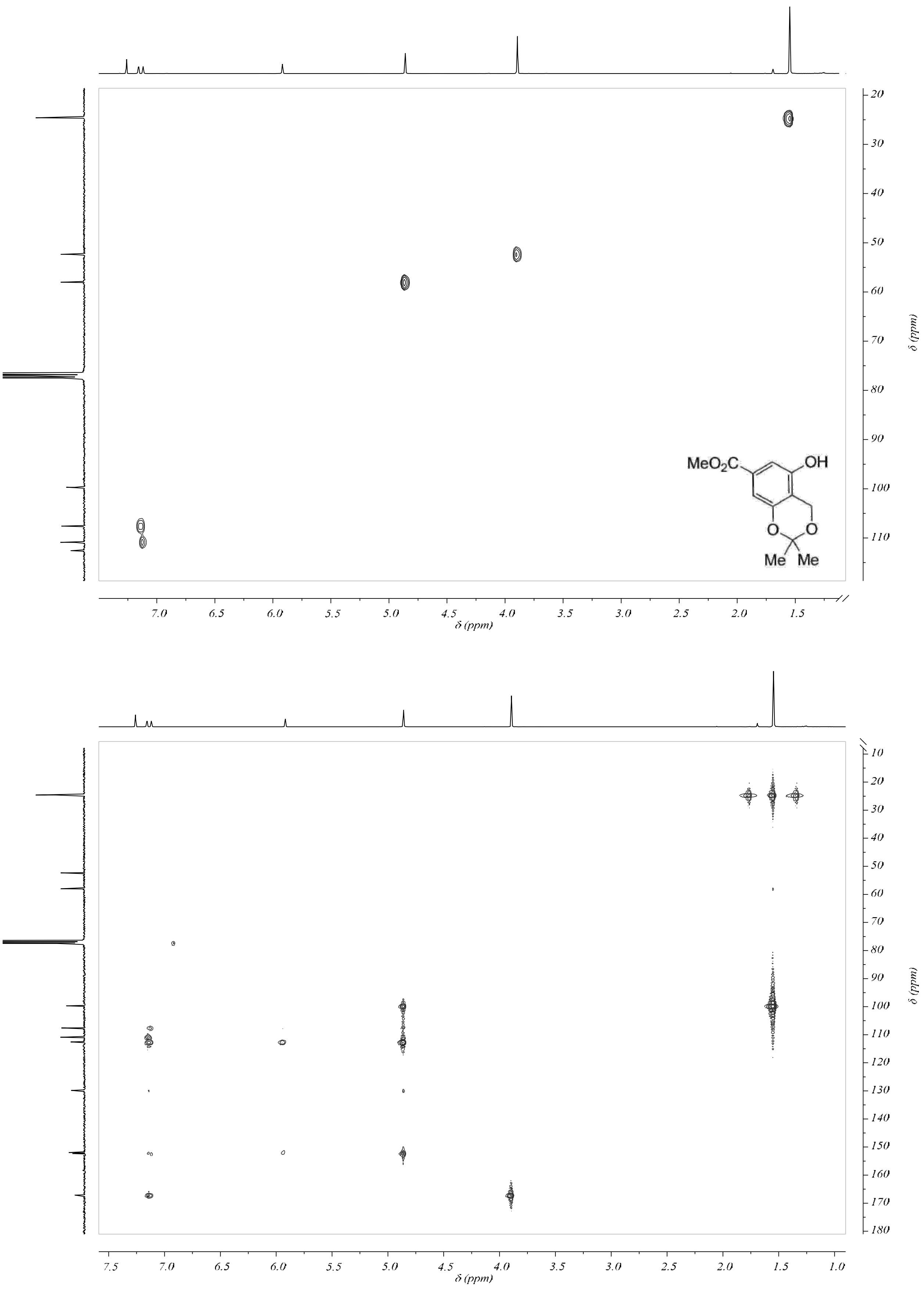

Figure S2. HSQC (top) and HMBC (bottom) spectra of compound **17** in $CDCl_3$.

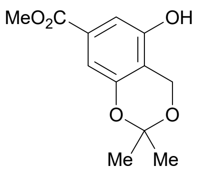

Acquisition Parameter

Source Type	ESI	Ion Polarity	Positive	Set Nebulizer	3.0 Bar
Focus	Not active	Set Capillary	4000 V	Set Dry Heater	200 °C
Scan Begin	100 m/z	Set End Plate Offset	-500 V	Set Dry Gas	6.0 l/min
Scan End	1300 m/z	Set Collision Cell RF	250.0 Vpp	Set Divert Valve	Source

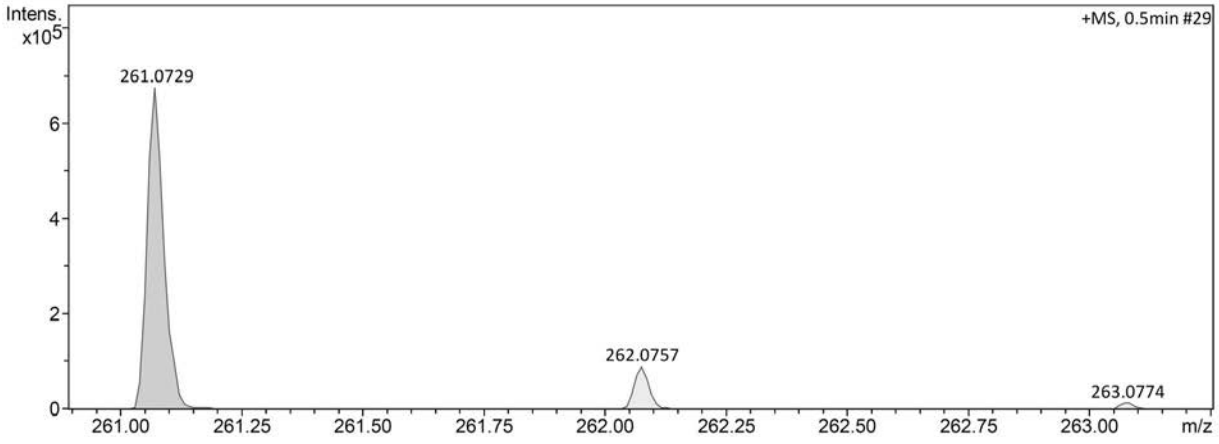

Meas. m/z	#	Ion Formula	m/z	err [ppm]	mSigma	# mSigma	Score	rdb	e¯ Conf	N-Rule
261.0729	1	C12H14NaO5	261.0733	1.7	1.1	1	100.00	5.5	even	ok

Figure S3. High-resolution mass spectrum of compound **17**.

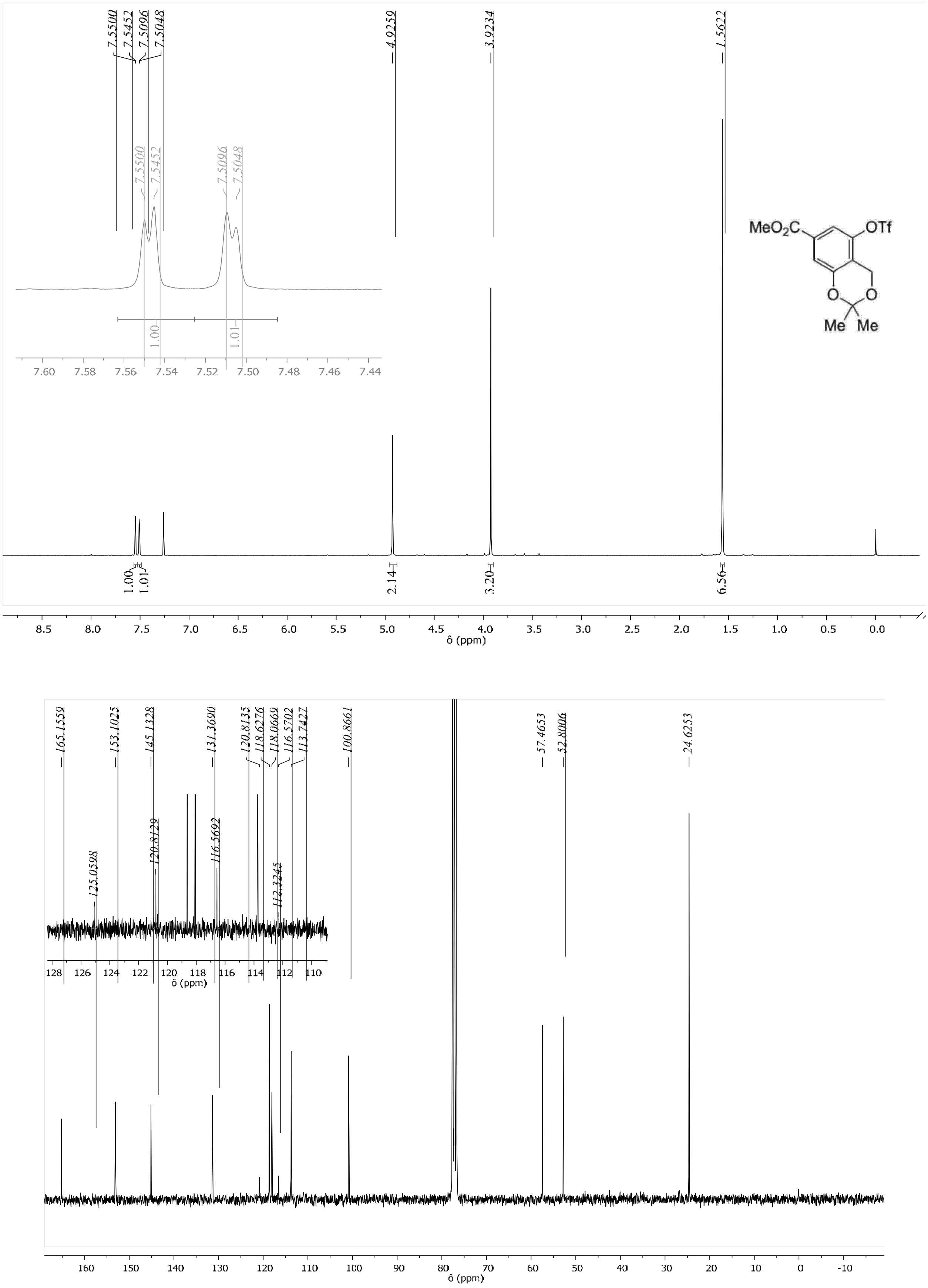

Figure S4. 300 MHz ^{1}H (top) and 75 MHz ^{13}C (bottom) NMR spectra of compound **18** in $CDCl_3$.

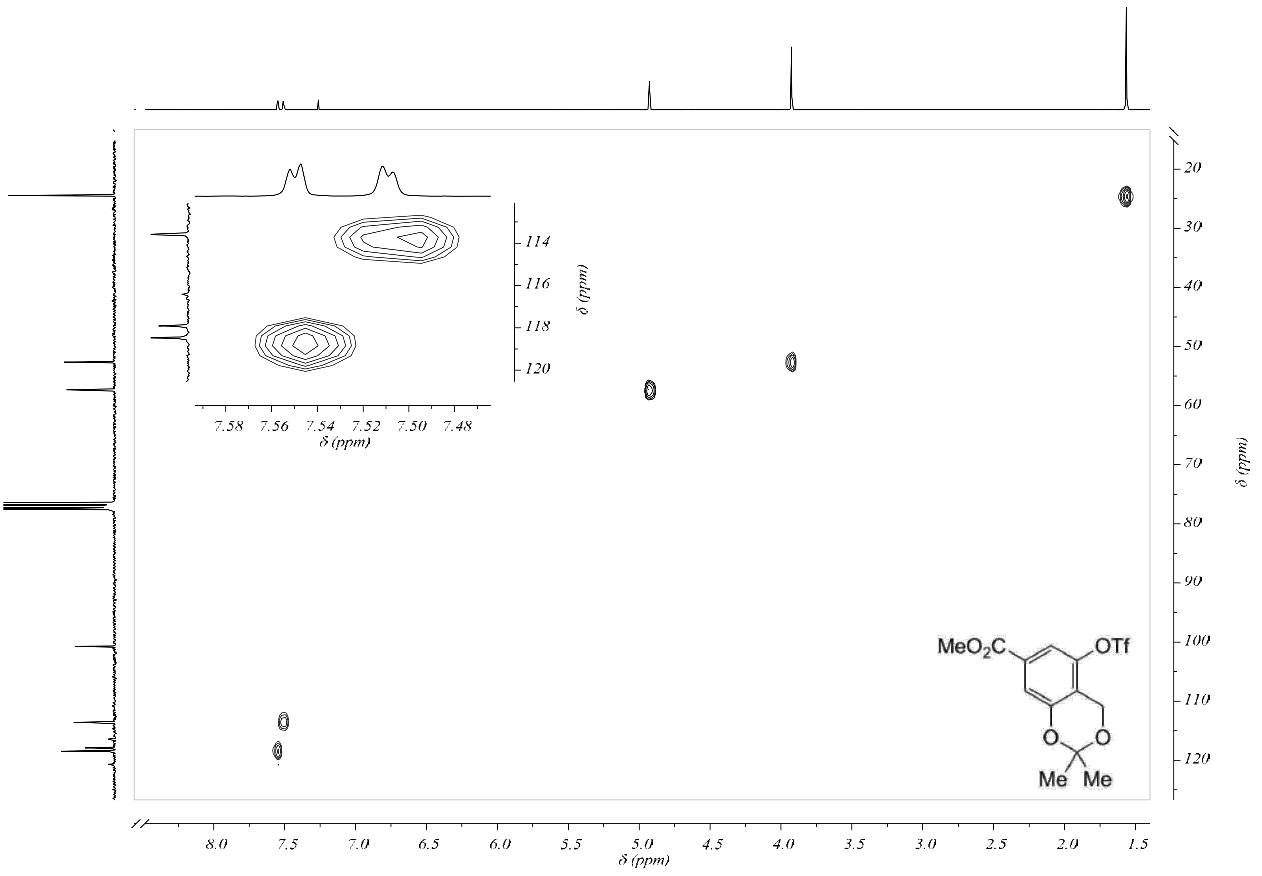

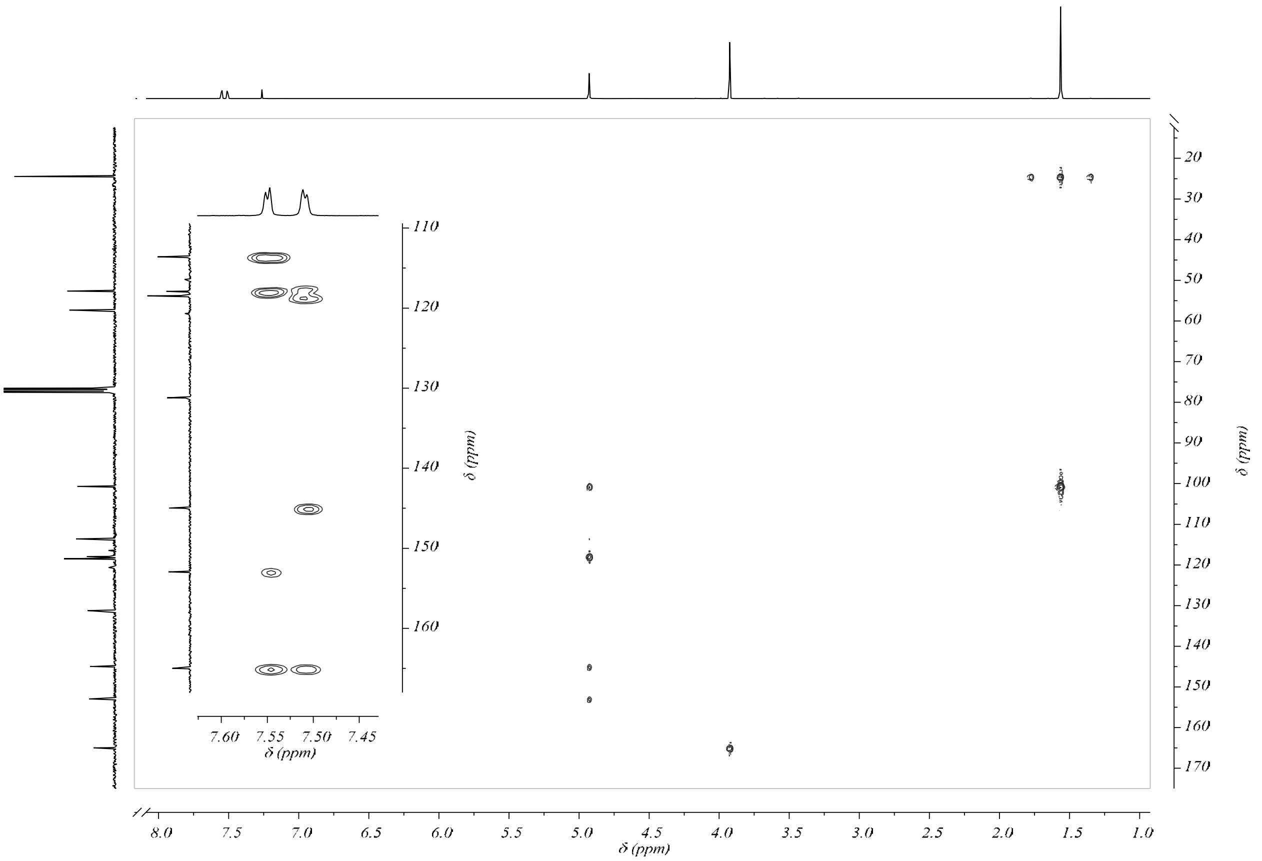

Figure S5. HSQC (top) and HMBC (bottom) spectra of compound **18** in $CDCl_3$.

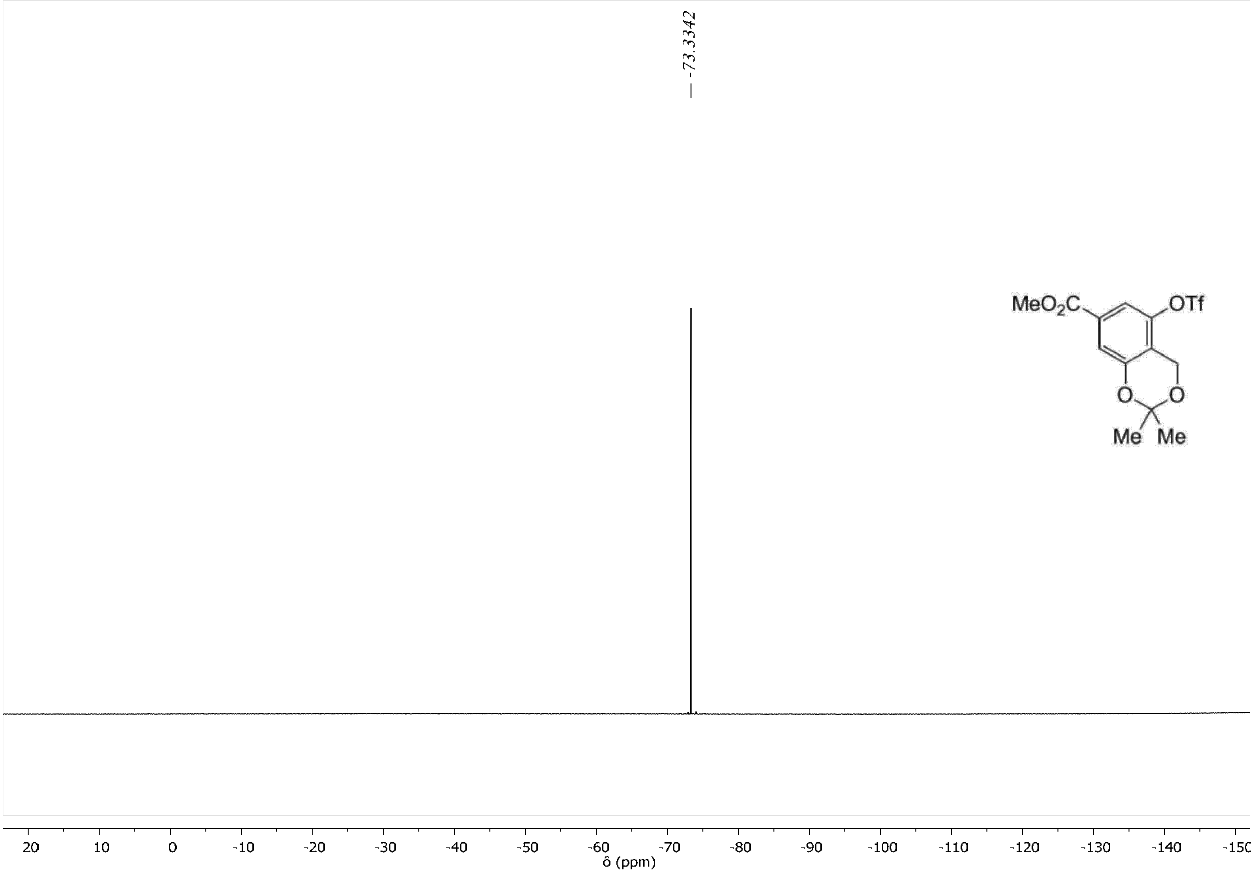

Acquisition Parameter

Source Type	ESI	Ion Polarity	Positive	Set Nebulizer	3.0 Bar
Focus	Not active	Set Capillary	4000 V	Set Dry Heater	200 °C
Scan Begin	100 m/z	Set End Plate Offset	-500 V	Set Dry Gas	6.0 l/min
Scan End	1300 m/z	Set Collision Cell RF	250.0 Vpp	Set Divert Valve	Source

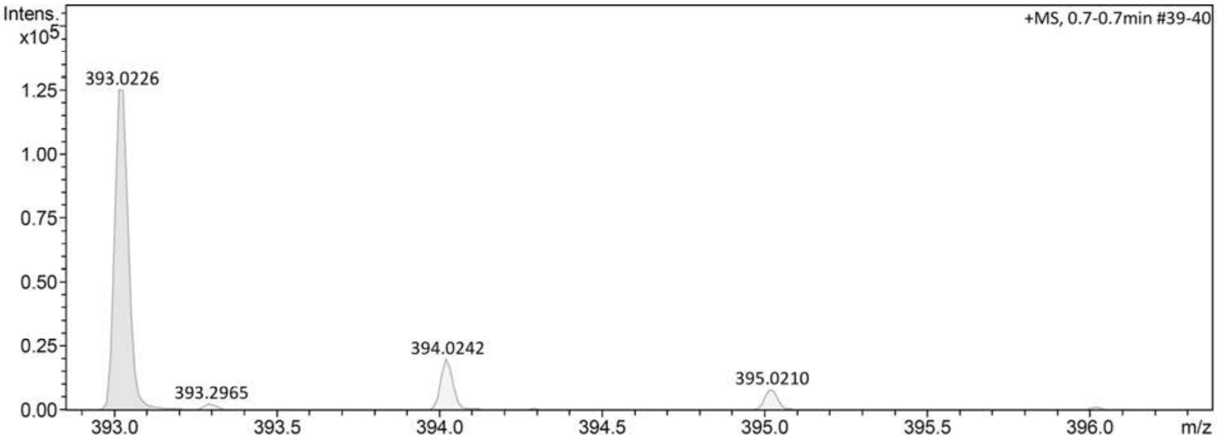

Meas. m/z	#	Ion Formula	m/z	err [ppm]	mSigma	# mSigma	Score	rdb	e⁻ Conf	N-Rule
393.0226	1	C13H13F3NaO7S	393.0226	0.1	3.9	3	100.00	5.5	even	ok

Figure S6. 282 MHz ^{19}F NMR spectrum of compound **18** in $CDCl_3$ (top) and high-resolution mass spectrum of compound **18** (bottom).

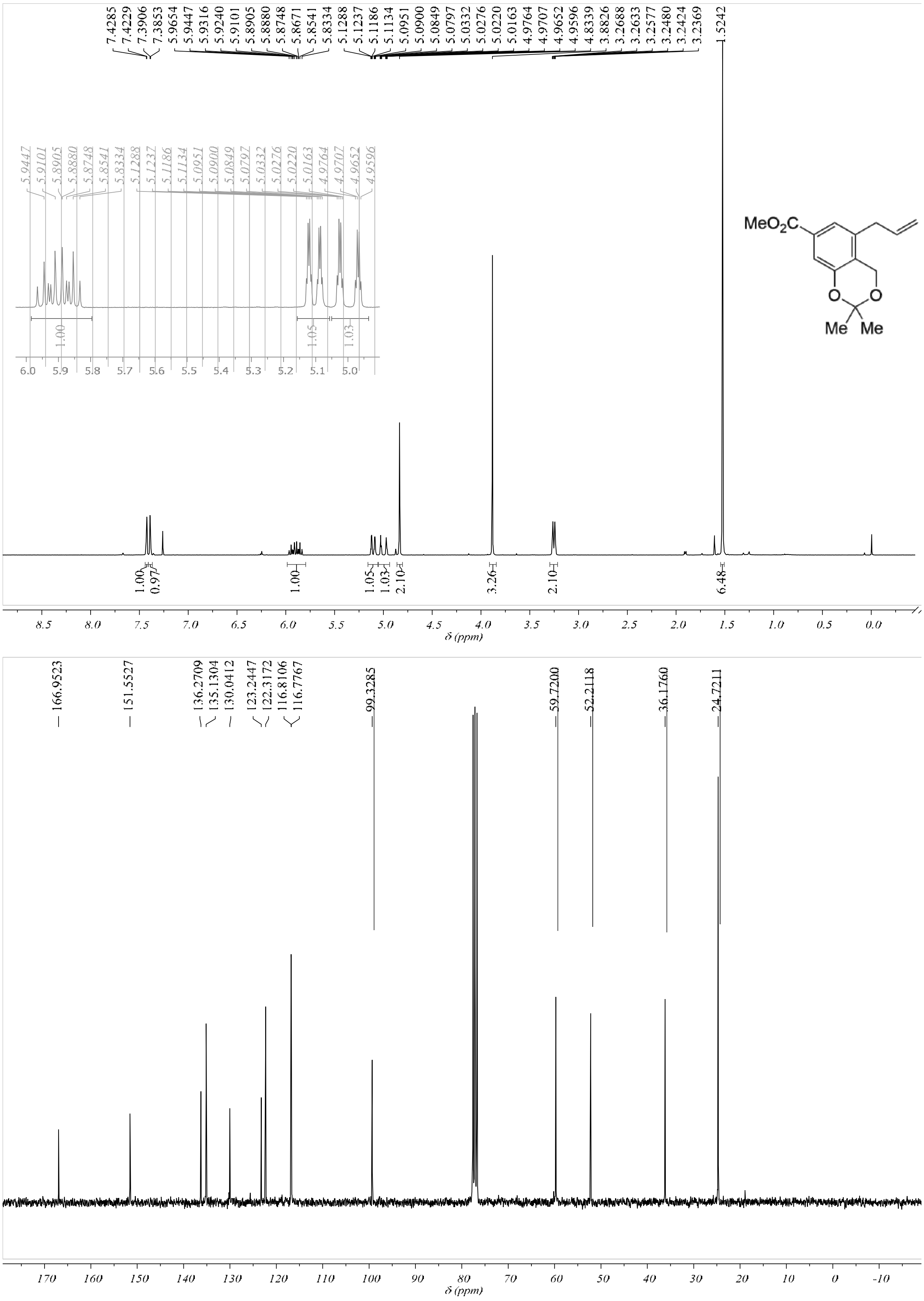

Figure S7. 300 MHz ^{1}H (top) and 75 MHz ^{13}C (bottom) NMR spectra of compound **19** in $CDCl_3$.

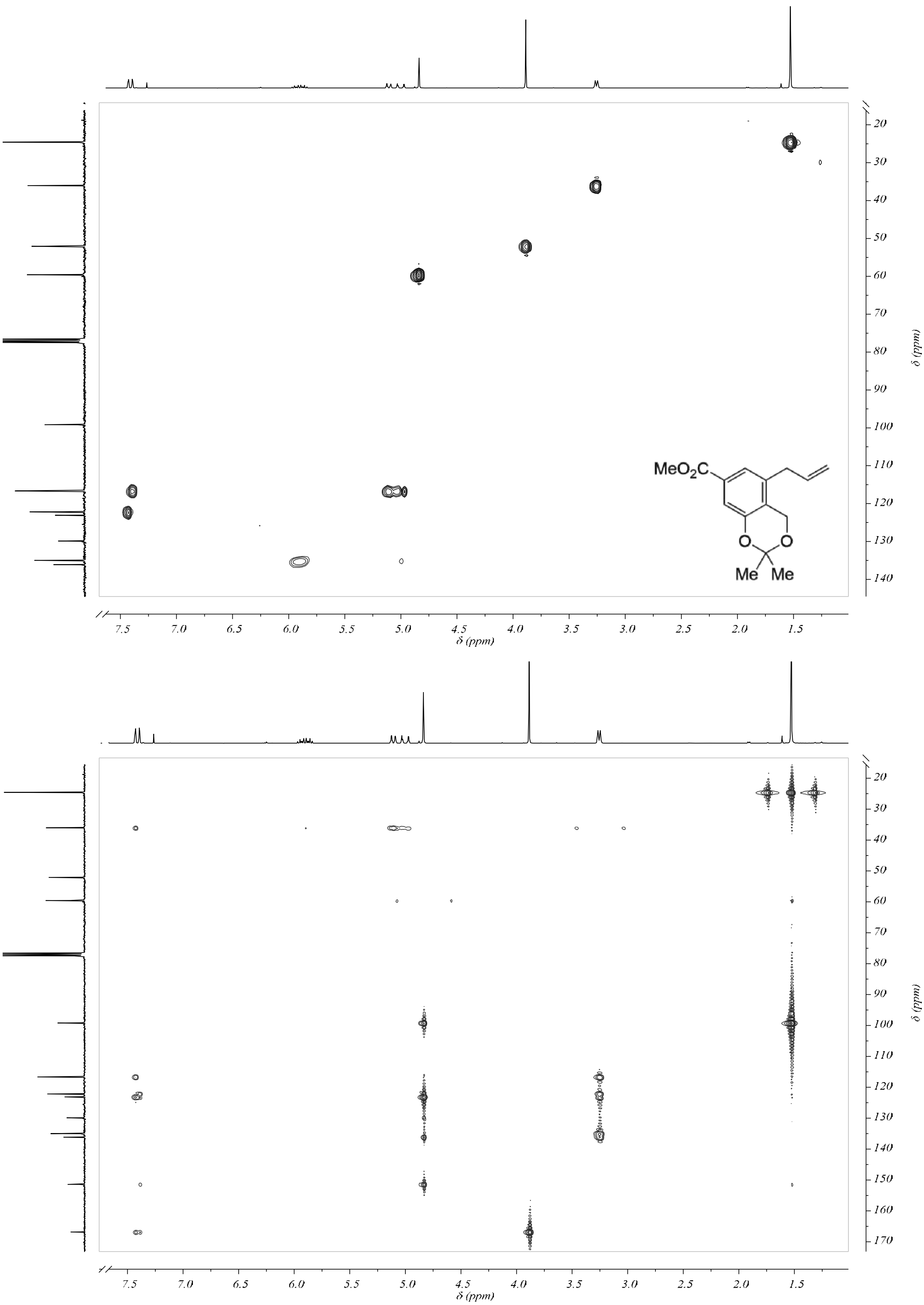

Figure S8. HSQC (top) and HMBC (bottom) spectra of compound **19** in $CDCl_3$.

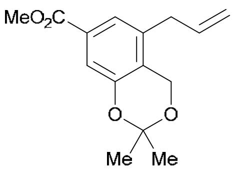

Acquisition Parameter

Source Type	ESI	Ion Polarity	Positive	Set Nebulizer	3.0 Bar
Focus	Not active	Set Capillary	4000 V	Set Dry Heater	200 °C
Scan Begin	100 m/z	Set End Plate Offset	-500 V	Set Dry Gas	6.0 l/min
Scan End	1300 m/z	Set Collision Cell RF	250.0 Vpp	Set Divert Valve	Source

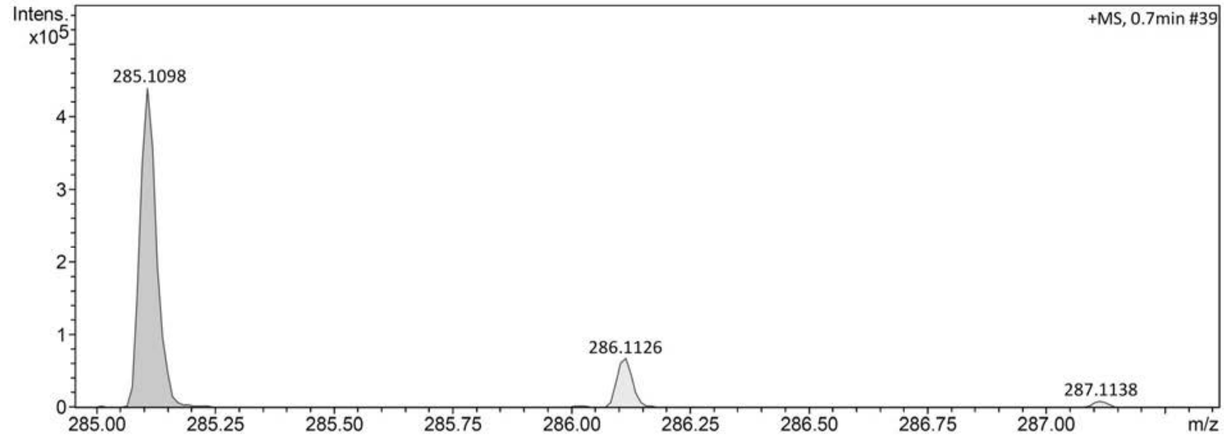

Meas. m/z	#	Ion Formula	m/z	err [ppm]	mSigma	# mSigma	Score	rdb	e⁻ Conf	N-Rule
285.1098	1	C15H18NaO4	285.1097	-0.4	7.4	1	100.00	6.5	even	ok

Figure S9. High-resolution mass spectrum of compound **19**.

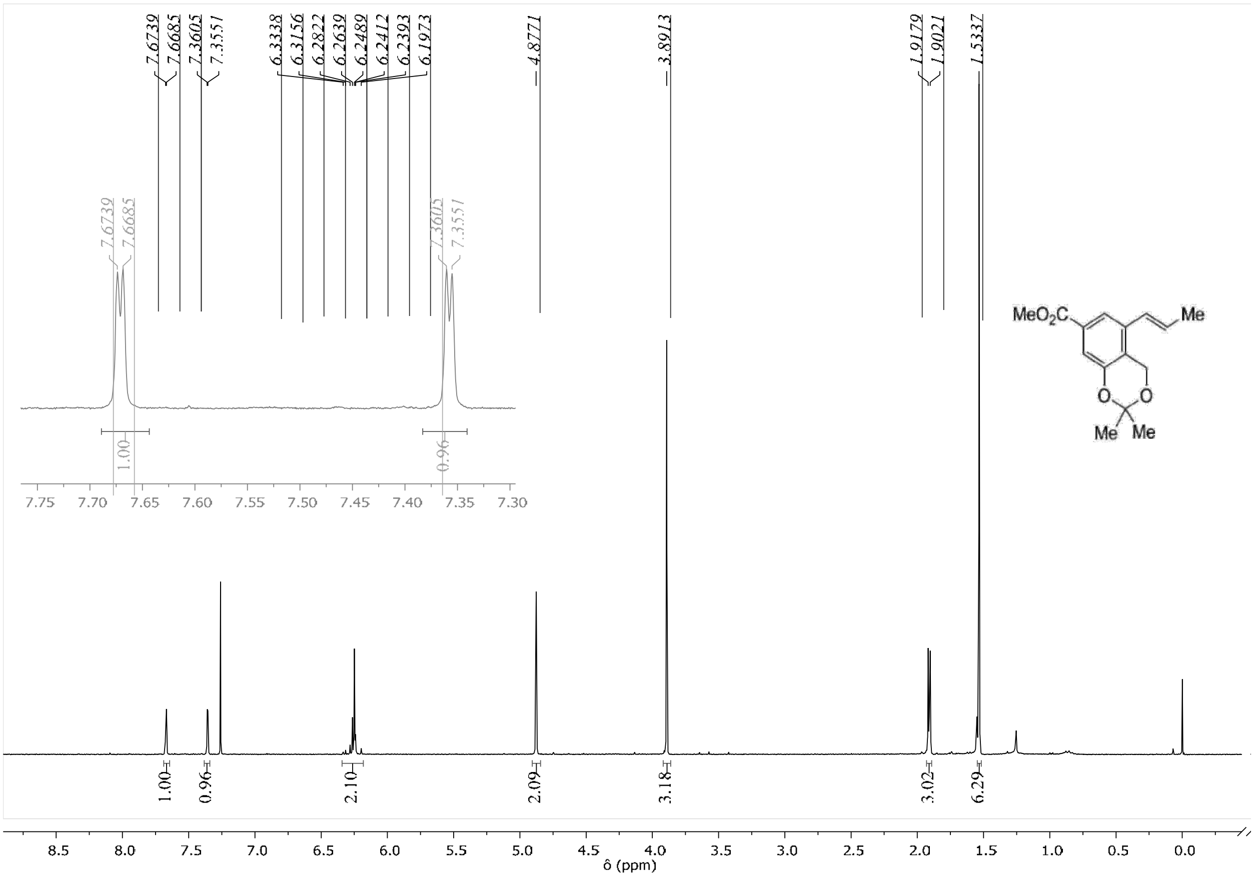

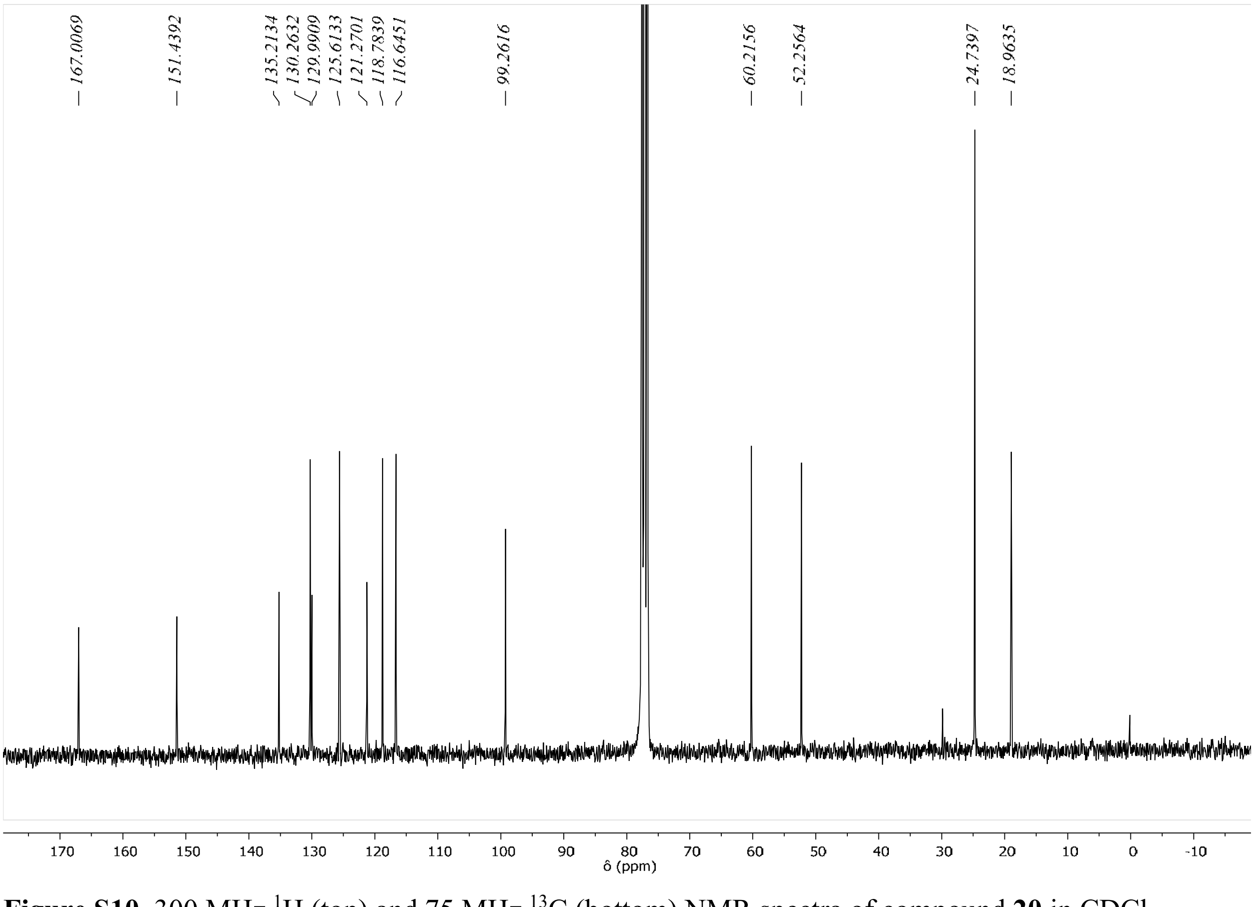

Figure S10. 300 MHz ^{1}H (top) and 75 MHz ^{13}C (bottom) NMR spectra of compound **20** in $CDCl_3$.

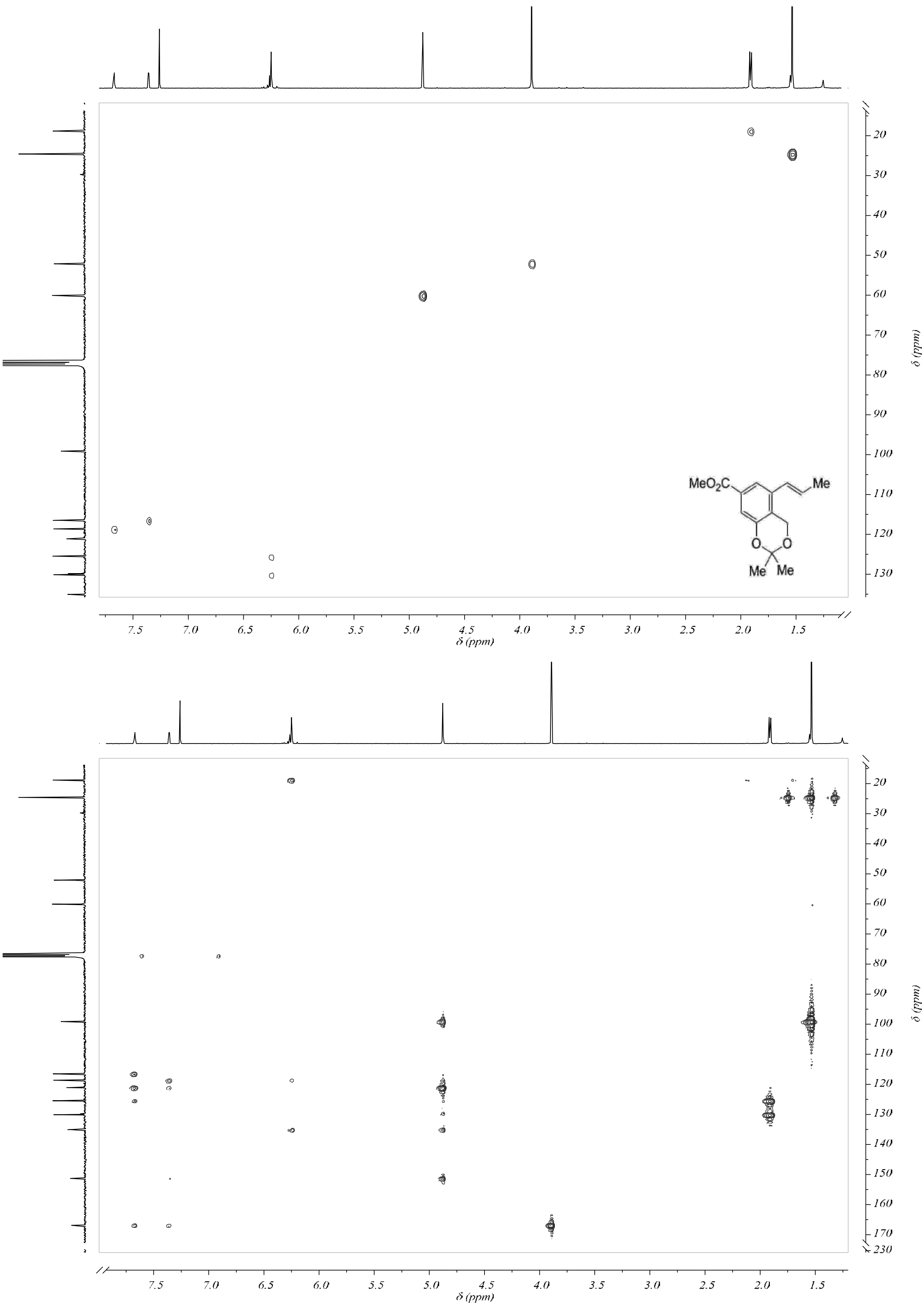

Figure S11. HSQC (top) and HMBC (bottom) spectra of compound **20** in $CDCl_3$.

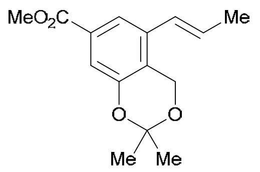

Acquisition Parameter

Source Type	ESI	Ion Polarity	Positive	Set Nebulizer	3.0 Bar
Focus	Not active	Set Capillary	4000 V	Set Dry Heater	200 °C
Scan Begin	100 m/z	Set End Plate Offset	-500 V	Set Dry Gas	6.0 l/min
Scan End	1300 m/z	Set Collision Cell RF	250.0 Vpp	Set Divert Valve	Source

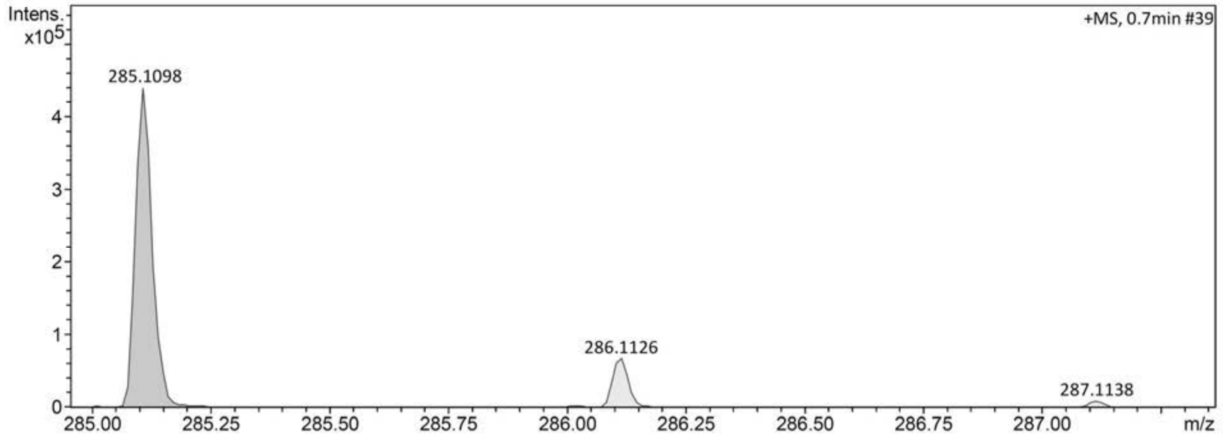

Meas. m/z	#	Ion Formula	m/z	err [ppm]	mSigma	# mSigma	Score	rdb	e⁻ Conf	N-Rule
285.1098	1	C15H18NaO4	285.1097	-0.4	7.4	1	100.00	6.5	even	ok

Figure S12. High-resolution mass spectrum of compound **20**.

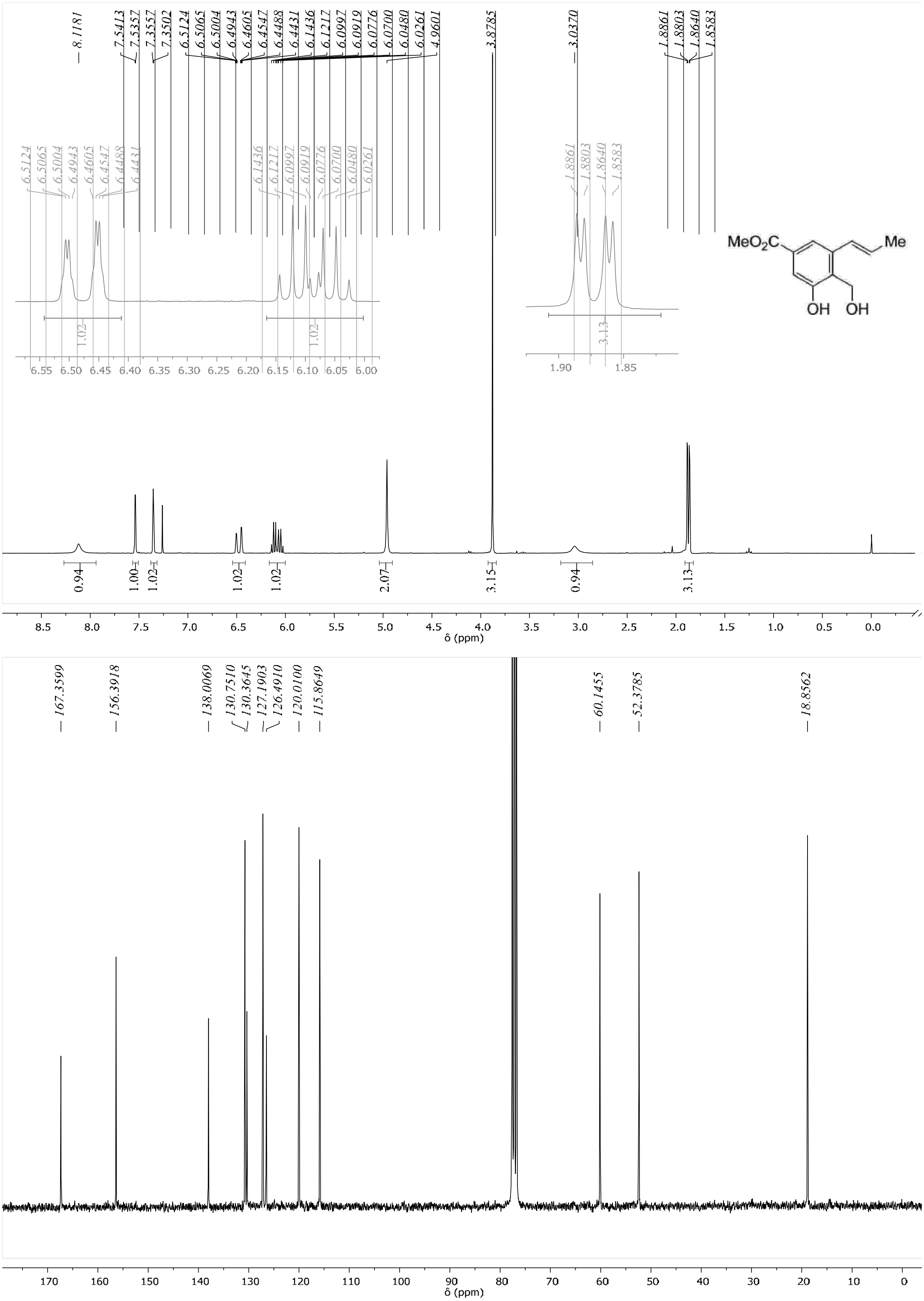

Figure S13. 300 MHz ^{1}H (top) and 75 MHz ^{13}C (bottom) NMR spectra of compound **24** in $CDCl_3$.

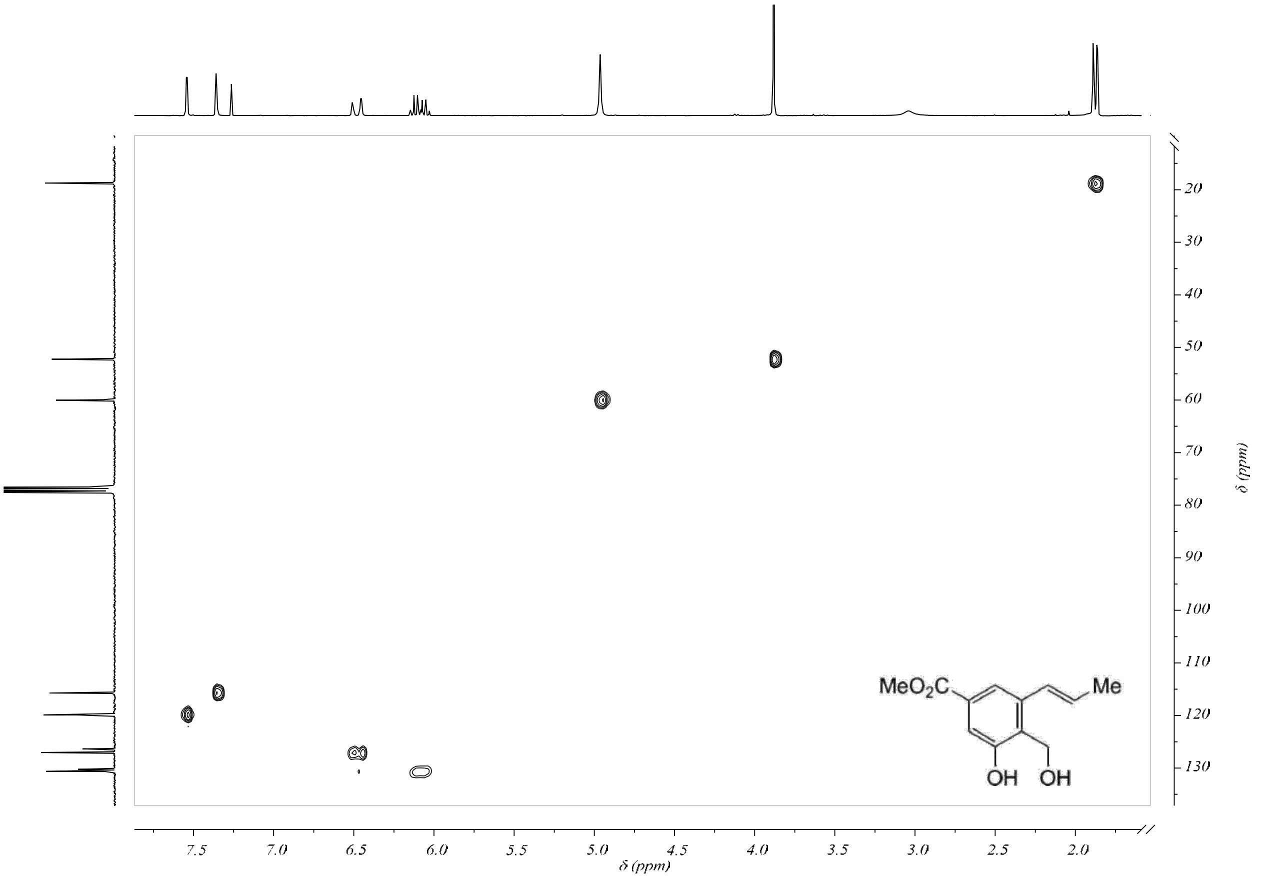

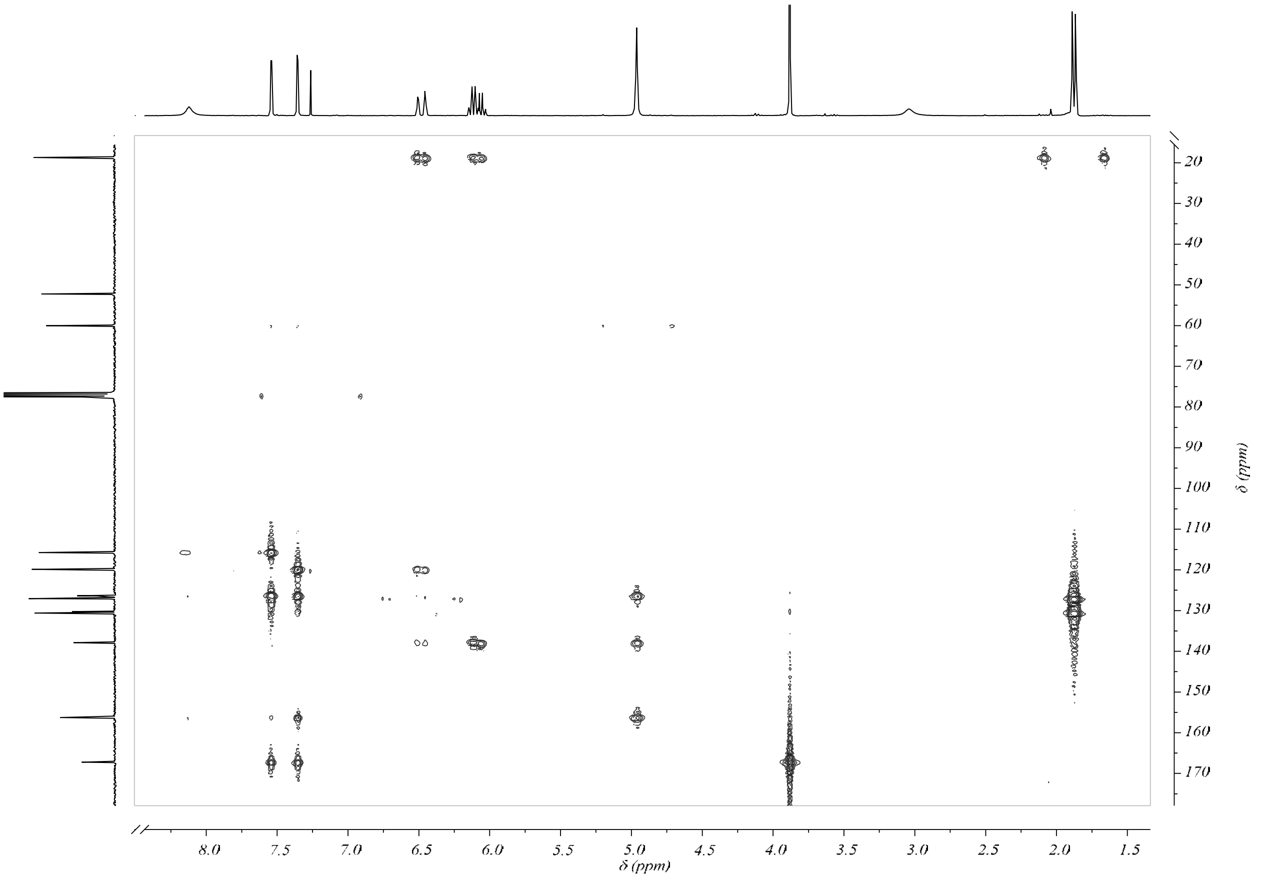

Figure S14. HSQC (top) and HMBC (bottom) spectra of compound **24** in $CDCl_3$.

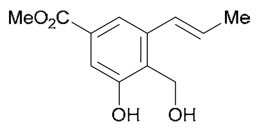

Acquisition Parameter

Source Type	ESI	Ion Polarity	Positive	Set Nebulizer	3.0 Bar
Focus	Not active	Set Capillary	4000 V	Set Dry Heater	200 °C
Scan Begin	100 m/z	Set End Plate Offset	-500 V	Set Dry Gas	6.0 l/min
Scan End	1300 m/z	Set Collision Cell RF	250.0 Vpp	Set Divert Valve	Source

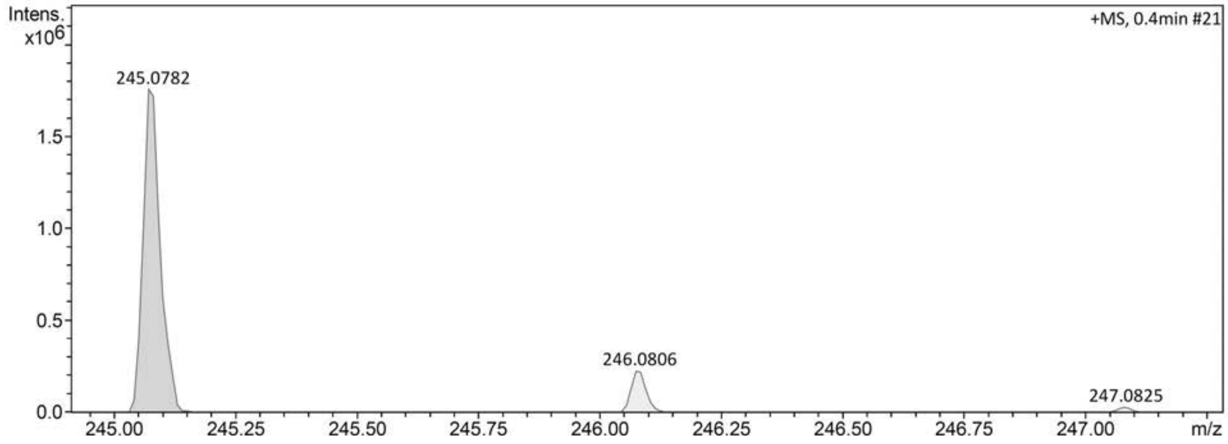

Meas. m/z	#	Ion Formula	m/z	err [ppm]	mSigma	# mSigma	Score	rdb	e⁻ Conf	N-Rule
245.0782	1	C12H14NaO4	245.0784	1.1	1.9	1	100.00	5.5	even	ok

Figure S15. High-resolution mass spectrum of compound **24**.

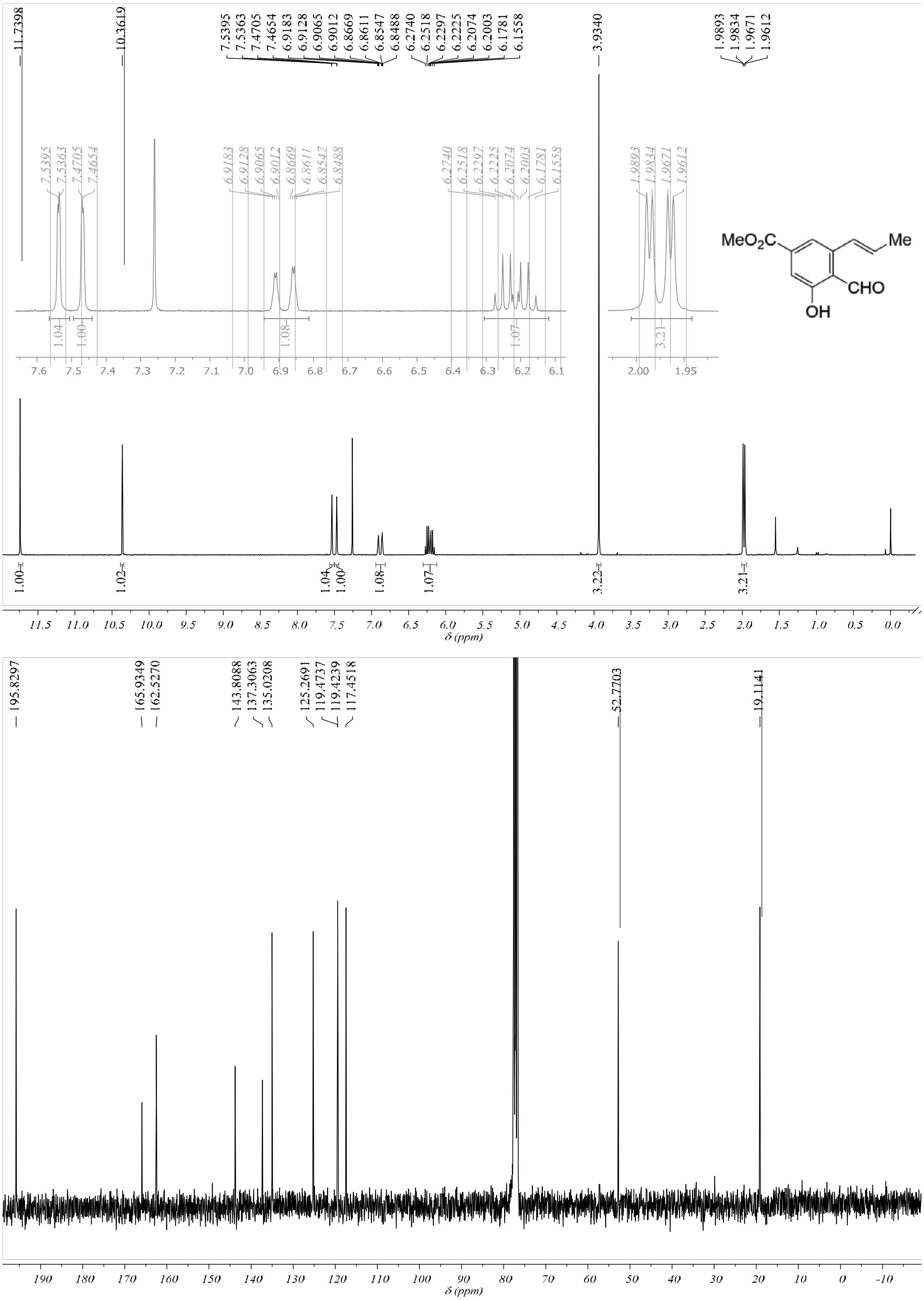

Figure S16. 300 MHz ^{1}H (top) and 75 MHz ^{13}C (bottom) NMR spectra of compound **25** in $CDCl_3$.

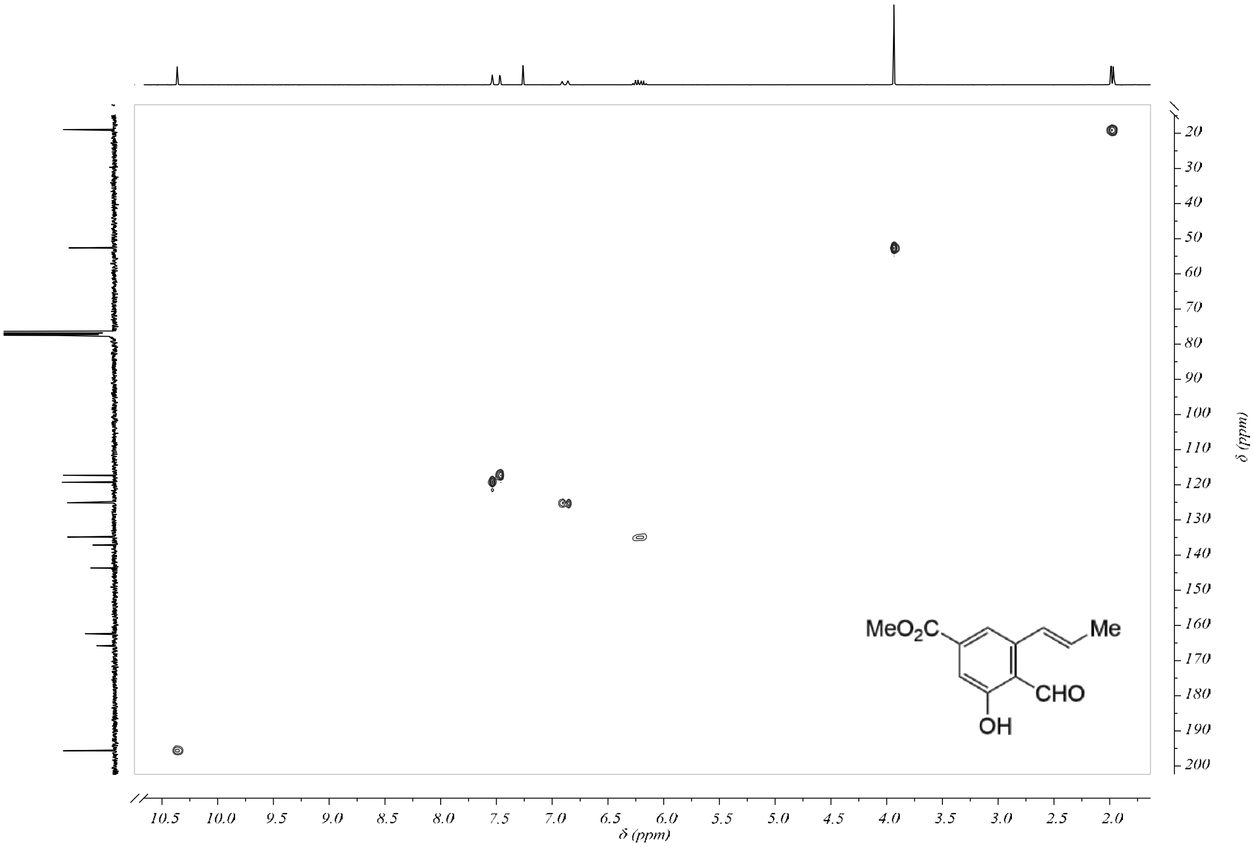

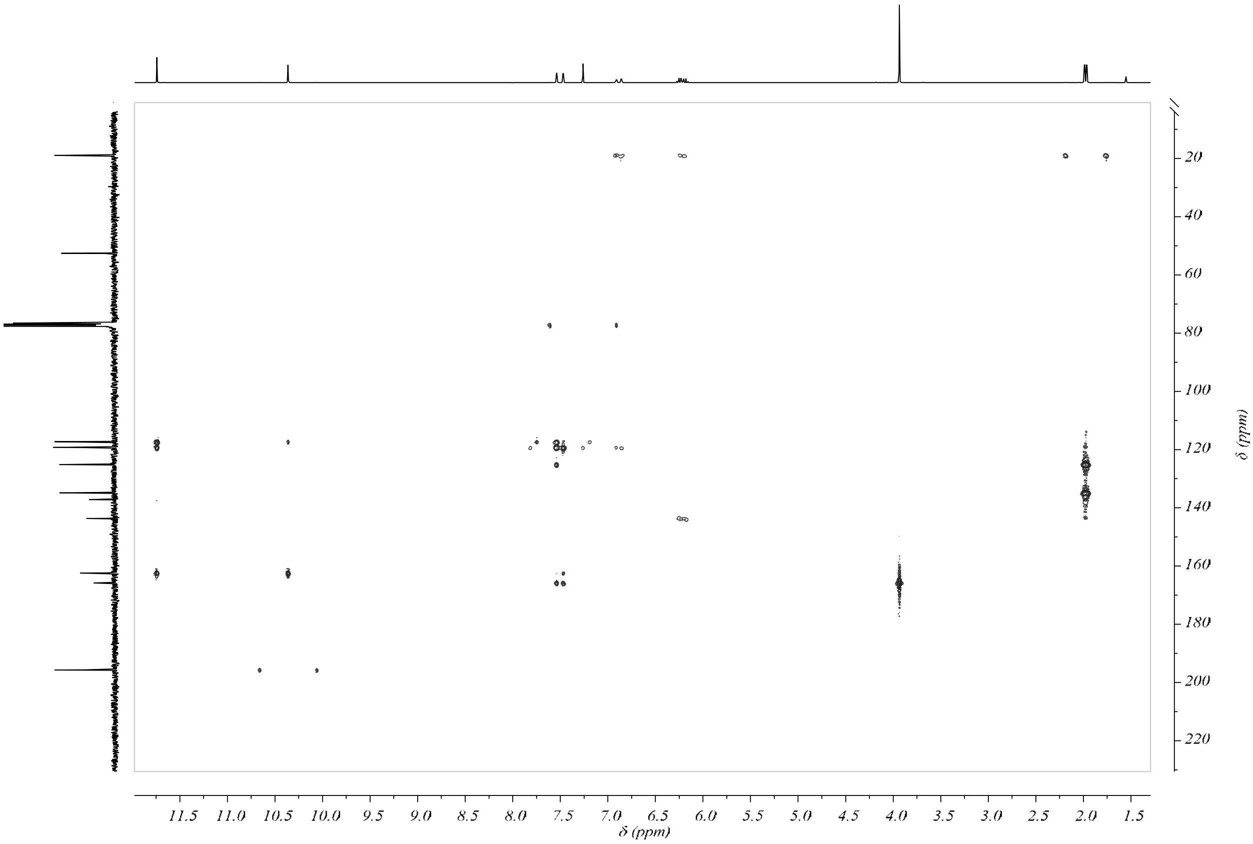

Figure S17. HSQC (top) and HMBC (bottom) spectra of compound **25** in $CDCl_3$.

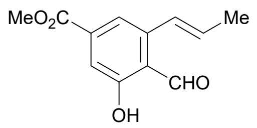

Acquisition Parameter

Source Type	ESI	Ion Polarity	Positive	Set Nebulizer	3.0 Bar
Focus	Not active	Set Capillary	4000 V	Set Dry Heater	200 °C
Scan Begin	100 m/z	Set End Plate Offset	-500 V	Set Dry Gas	6.0 l/min
Scan End	1300 m/z	Set Collision Cell RF	250.0 Vpp	Set Divert Valve	Source

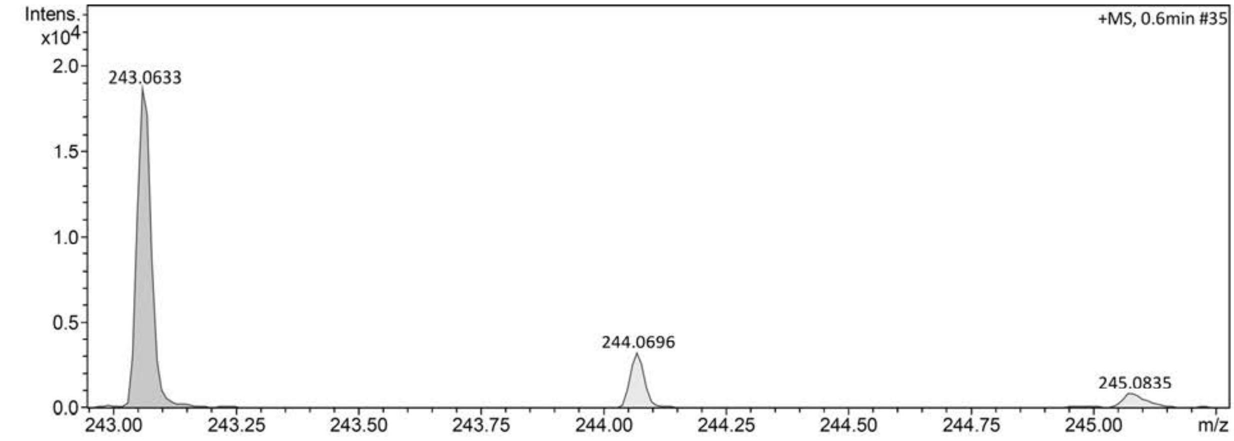

Meas. m/z	#	Ion Formula	m/z	err [ppm]	mSigma	# mSigma	Score	rdb	e¯ Conf	N-Rule
243.0633	1	C12H12NaO4	243.0628	-2.1	23.6	1	100.00	6.5	even	ok

Figure S18. High-resolution mass spectrum of compound **25**.

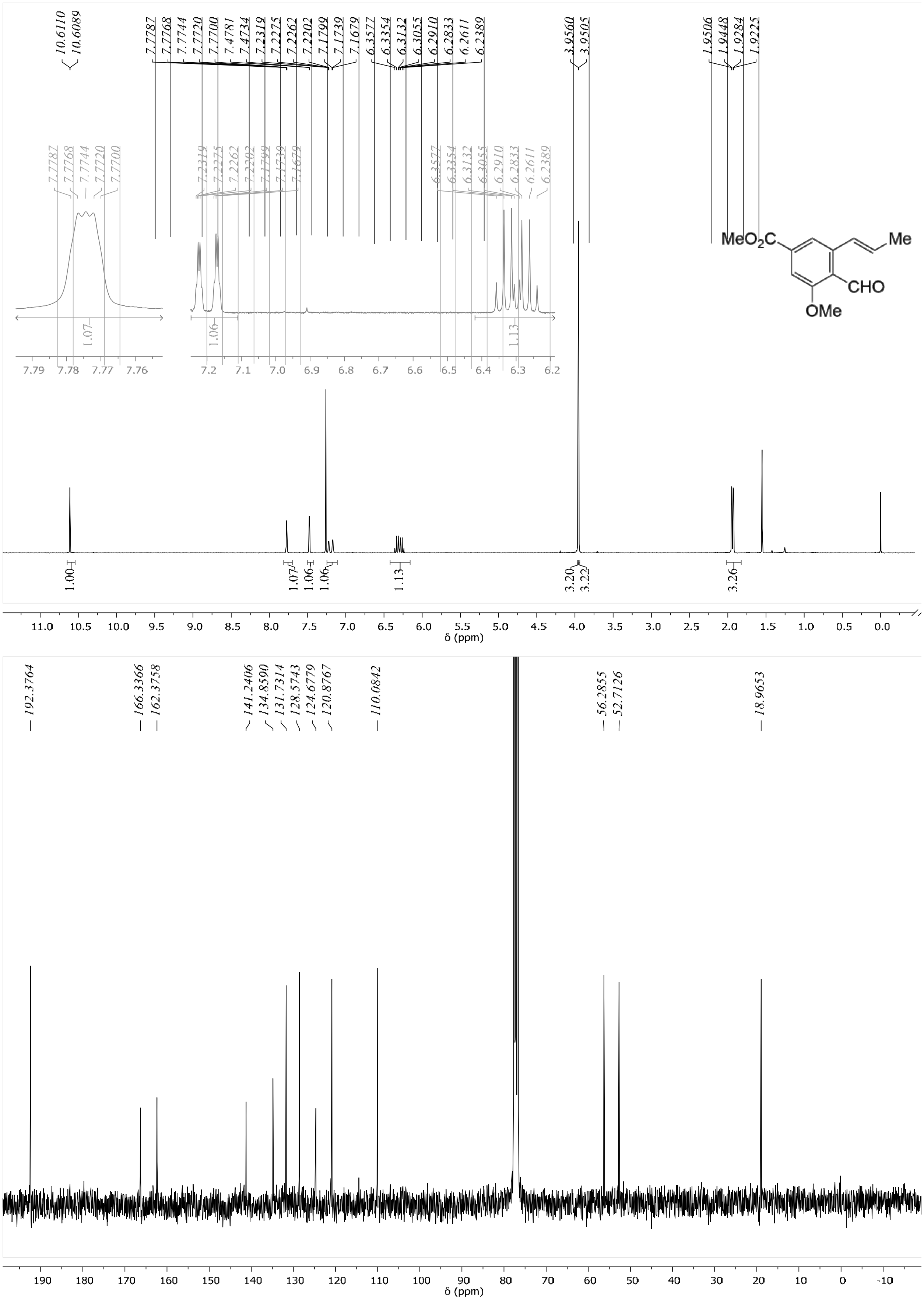

Figure S19. 300 MHz ^{1}H (top) and 75 MHz ^{13}C (bottom) NMR spectra of compound **31** in $CDCl_3$.

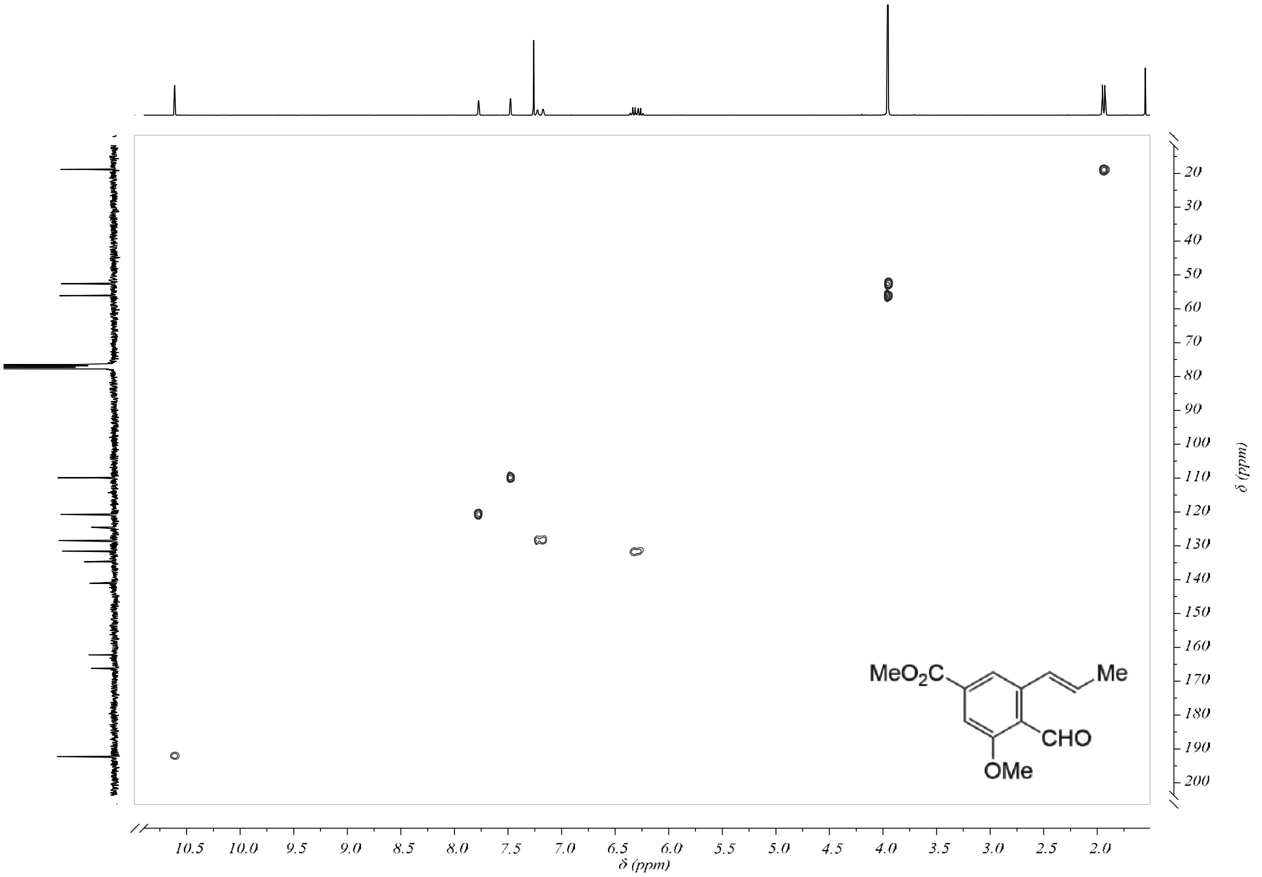

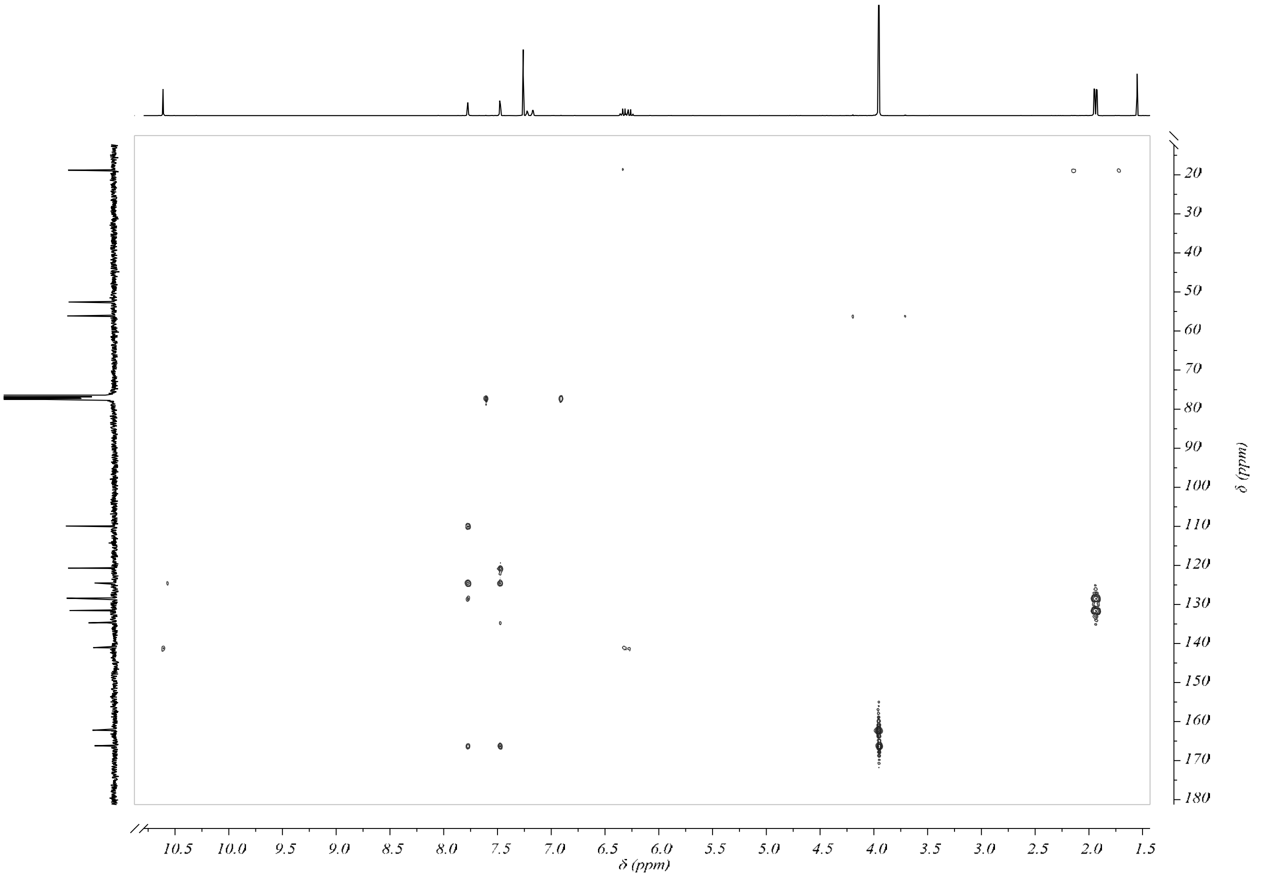

Figure S20. HSQC (top) and HMBC (bottom) spectra of compound **31** in $CDCl_3$.

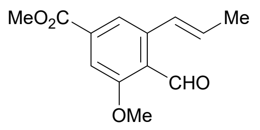

Acquisition Parameter

Source Type	ESI	Ion Polarity	Positive	Set Nebulizer	3.0 Bar		
Focus	Not active	Set Capillary	4000 V	Set Dry Heater	200 °C		
Scan Begin	100 m/z	Set End Plate Offset	-500 V	Set Dry Gas	6.0 l/min		
Scan End	1300 m/z	Set Collision Cell RF	250.0 Vpp	Set Divert Valve	Source		

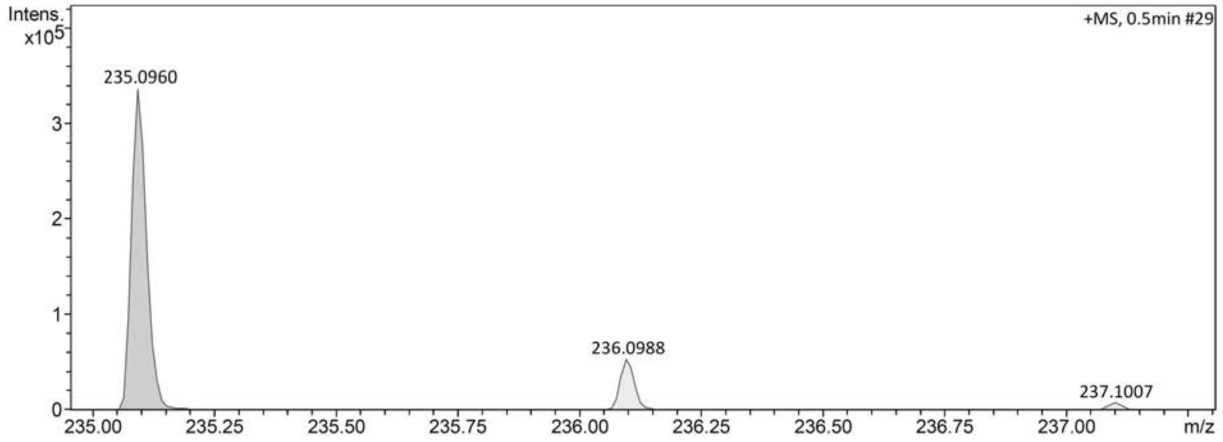

Meas. m/z	#	Ion Formula	m/z	err [ppm]	mSigma	# mSigma	Score	rdb	e⁻ Conf	N-Rule
235.0960	1	C13H15O4	235.0965	2.1	8.1	1	100.00	6.5	even	ok

Figure S21. High-resolution mass spectrum of compound **31**.

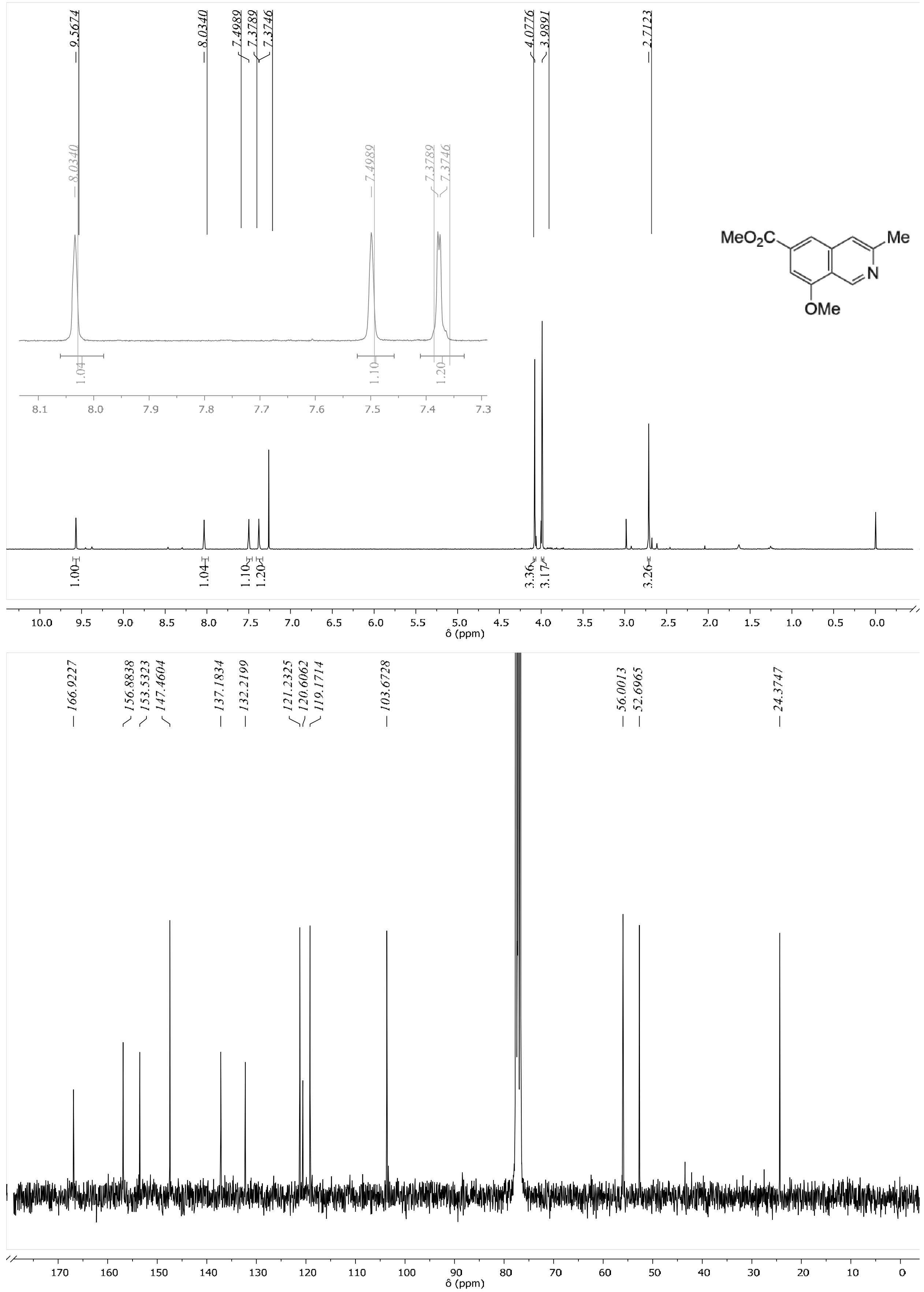

Figure S22. 300 MHz ^{1}H (top) and 75 MHz ^{13}C (bottom) NMR spectra of compound **5** in $CDCl_3$.

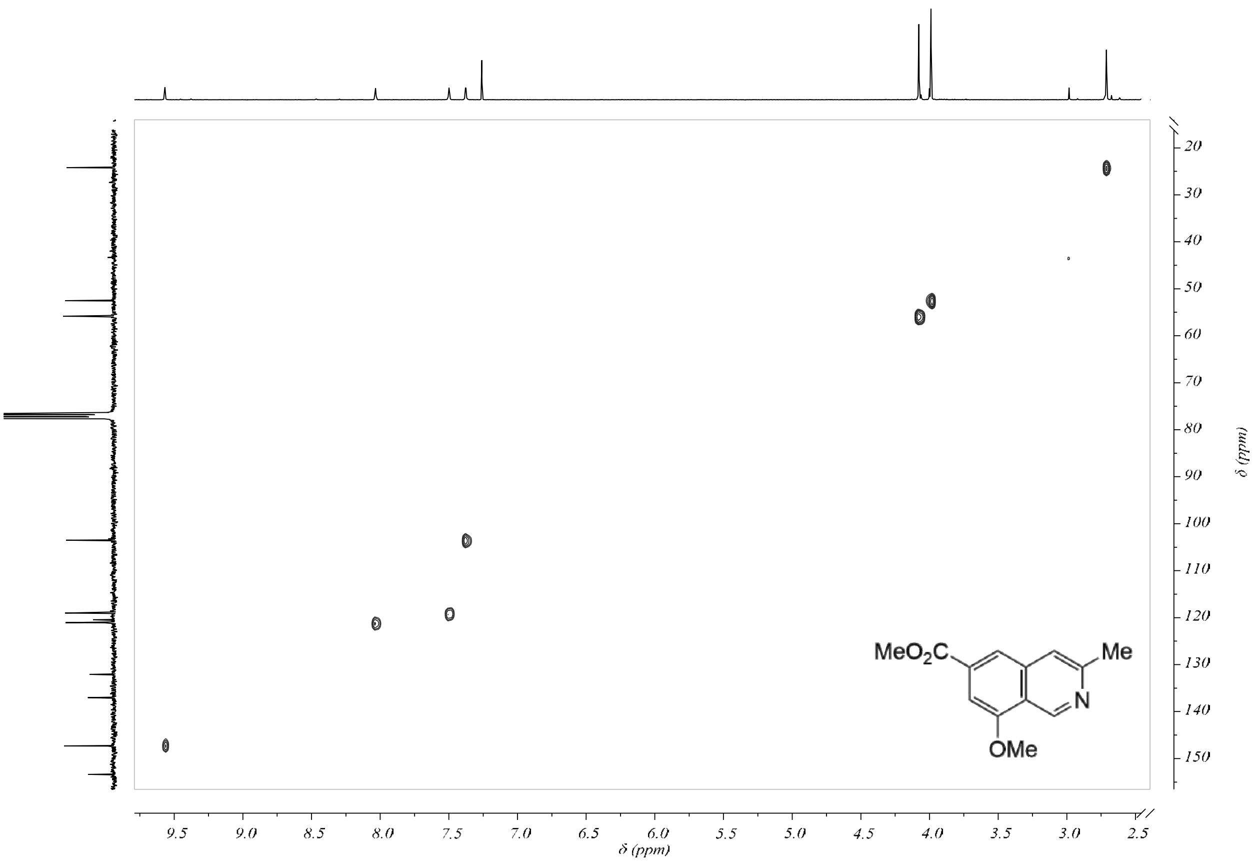

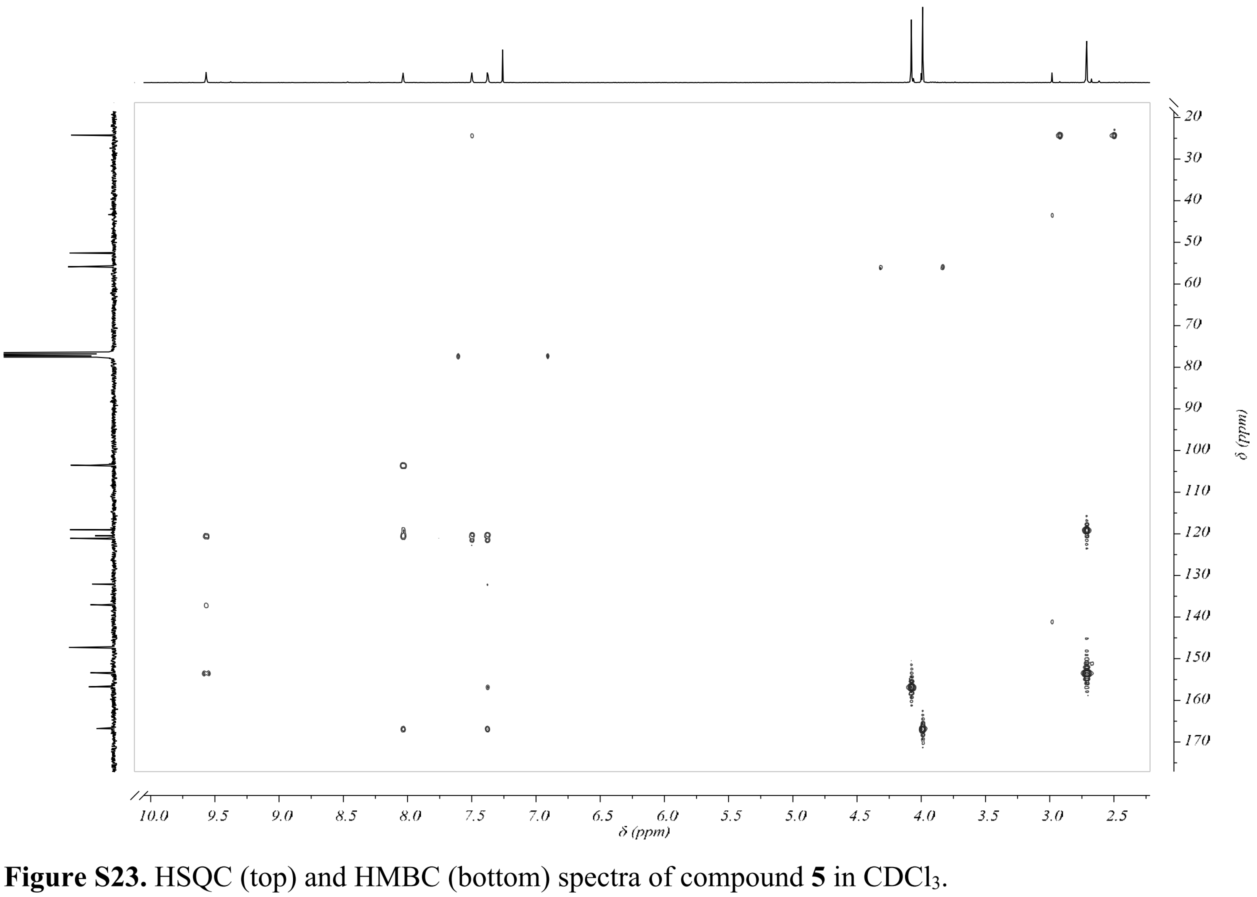

Figure S23. HSQC (top) and HMBC (bottom) spectra of compound **5** in $CDCl_3$.

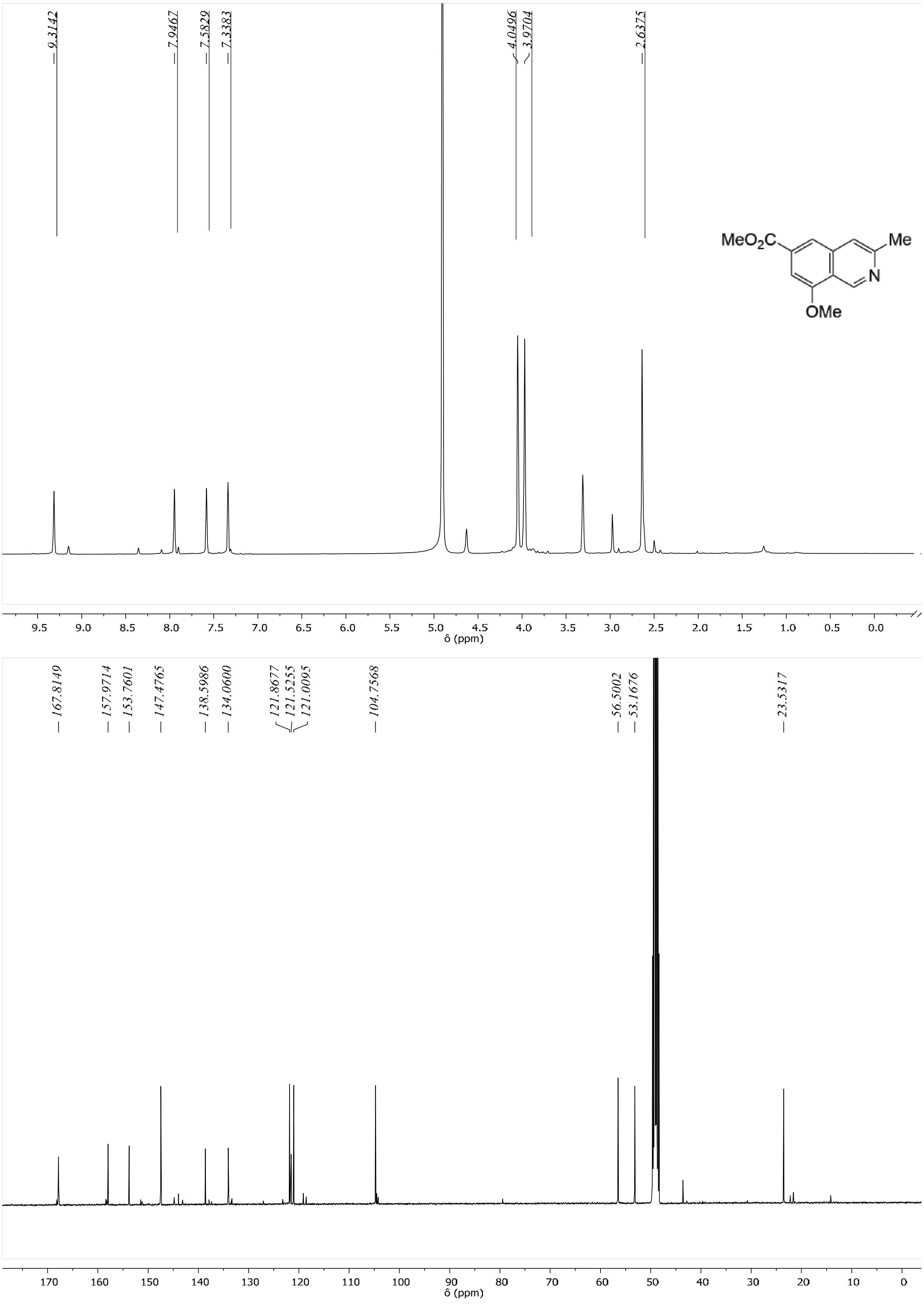

Figure S24. 400 MHz ^1H (top) and 100 MHz ^{13}C (bottom) NMR spectra of compound **5** in MeOD-d_4.

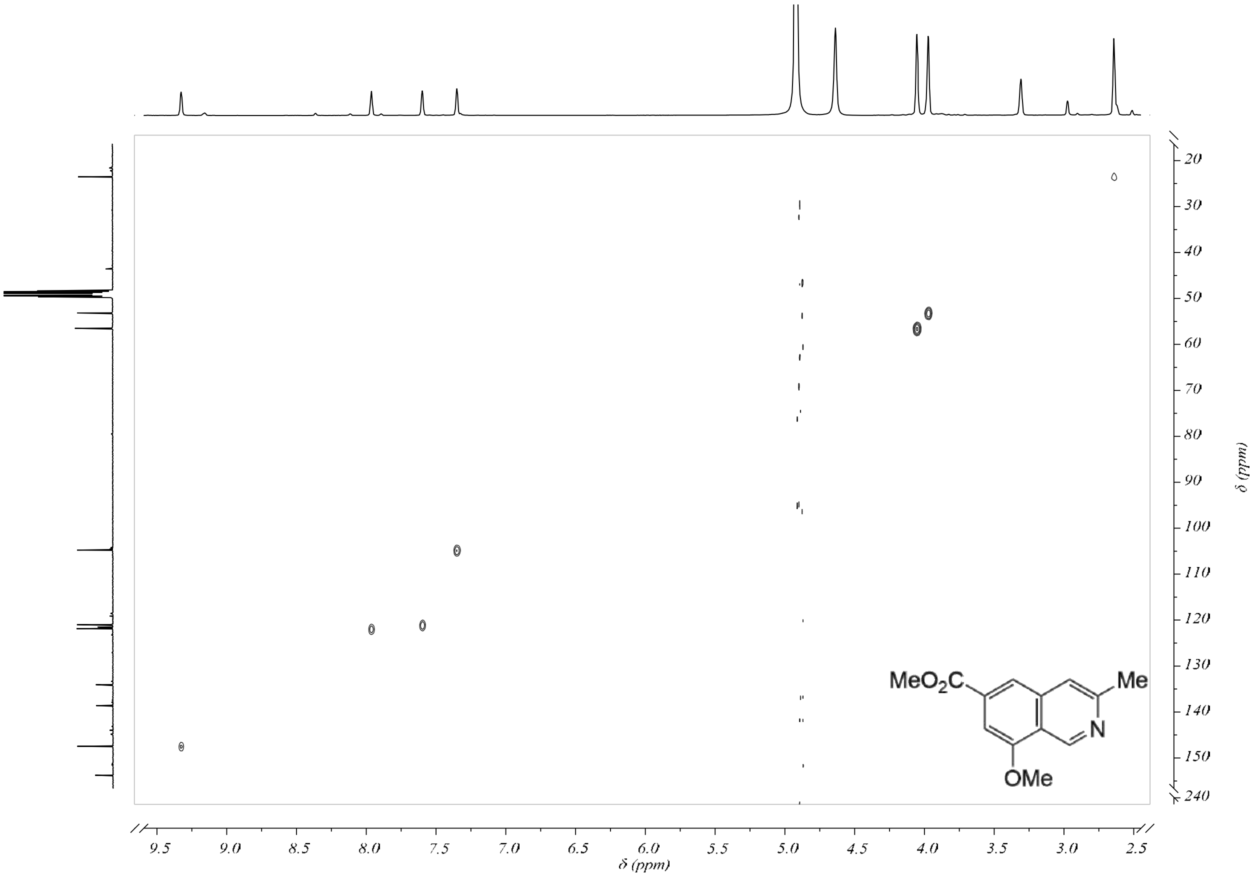

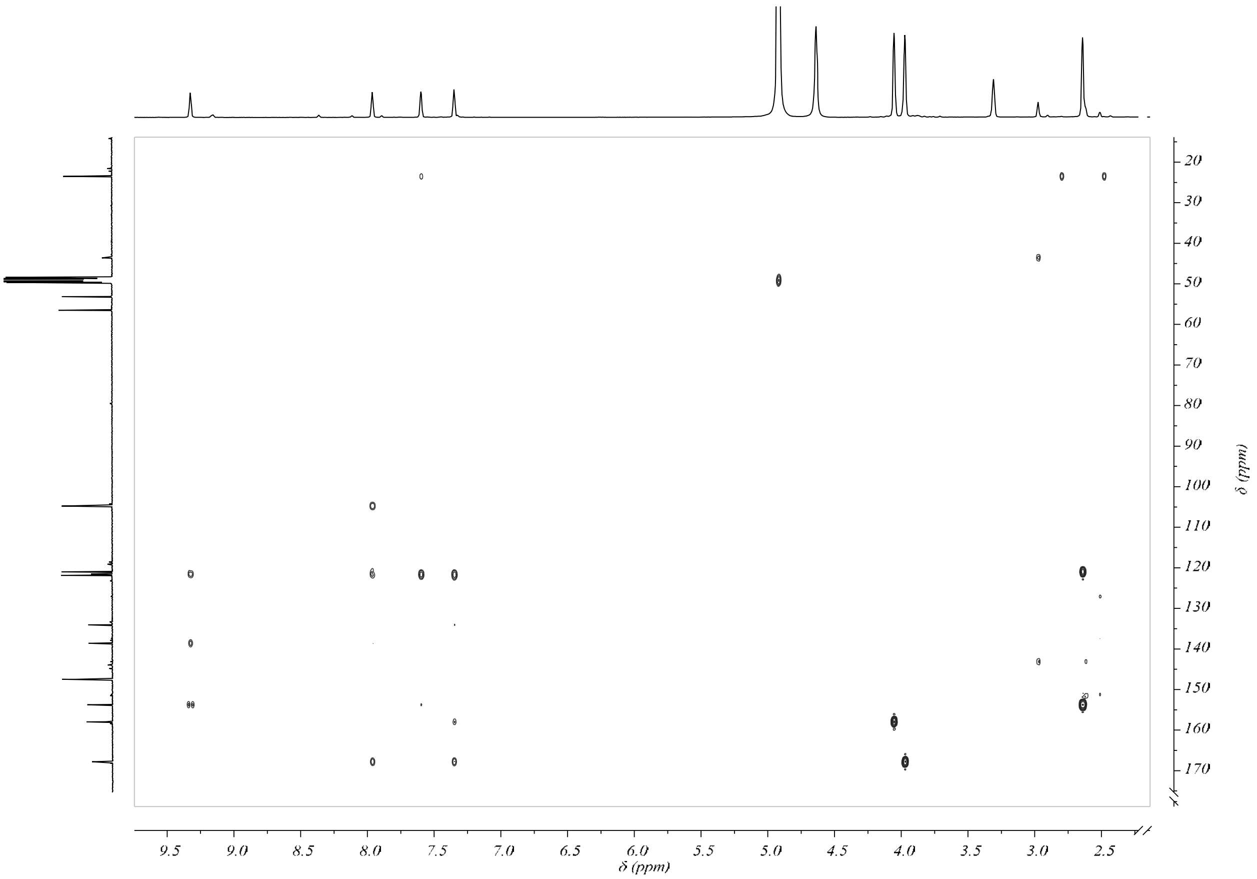

Figure S25. HSQC (top) and HMBC (bottom) spectra of compound **5** in MeOD-d_4.

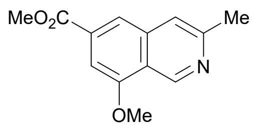

Acquisition Parameter

Source Type	ESI	Ion Polarity	Positive	Set Nebulizer	3.0 Bar
Focus	Not active	Set Capillary	4000 V	Set Dry Heater	200 °C
Scan Begin	100 m/z	Set End Plate Offset	-500 V	Set Dry Gas	6.0 l/min
Scan End	1300 m/z	Set Collision Cell RF	250.0 Vpp	Set Divert Valve	Source

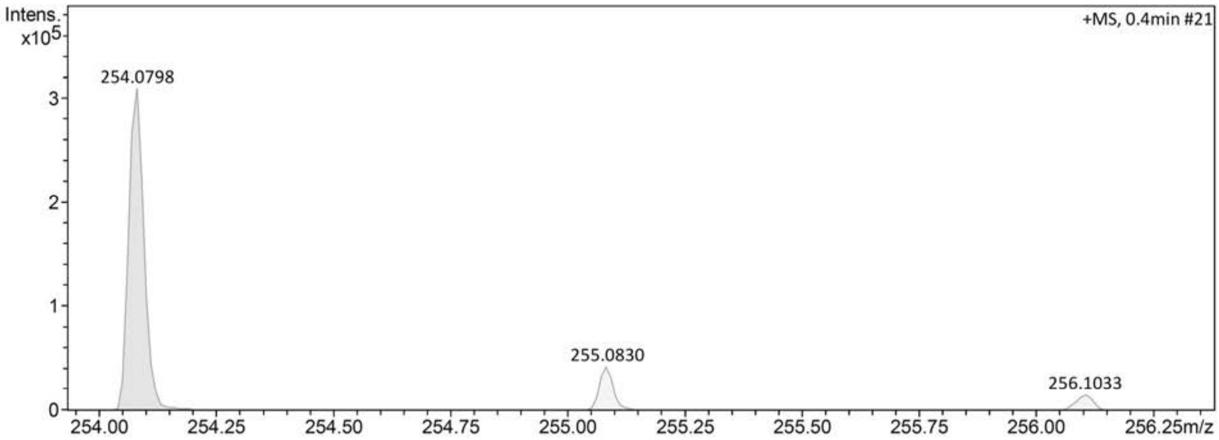

Meas. m/z	#	Ion Formula	m/z	err [ppm]	mSigma	# mSigma	Score	rdb	e⁻ Conf	N-Rule
254.0798	1	C13H13NNaO3	254.0788	-4.0	11.3	1	100.00	7.5	even	ok

Figure S26. High-resolution mass spectrum of compound **5**.

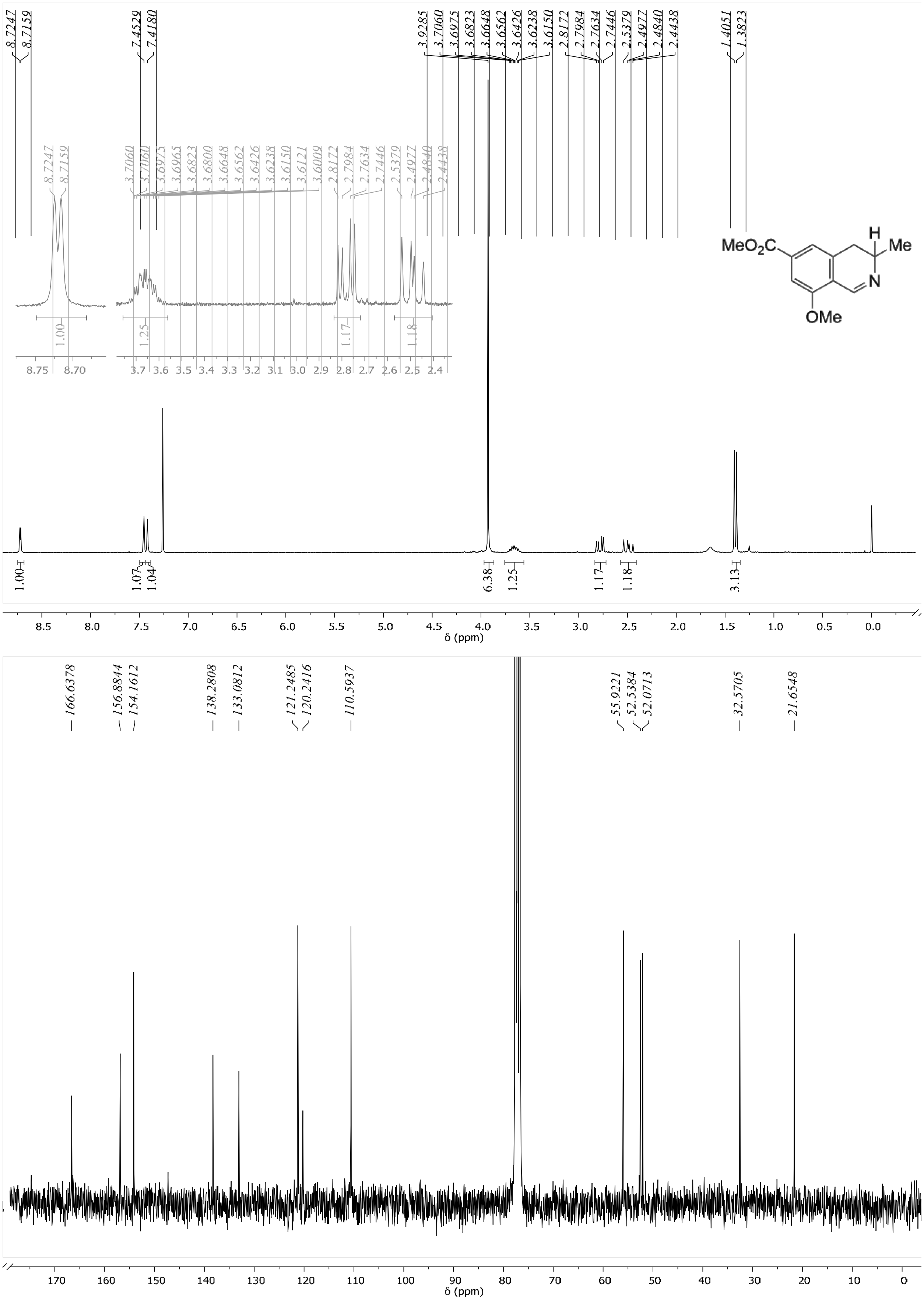

Figure S27. 300 MHz ^{1}H (top) and 75 MHz ^{13}C (bottom) NMR spectra of compound **33** in $CDCl_3$.

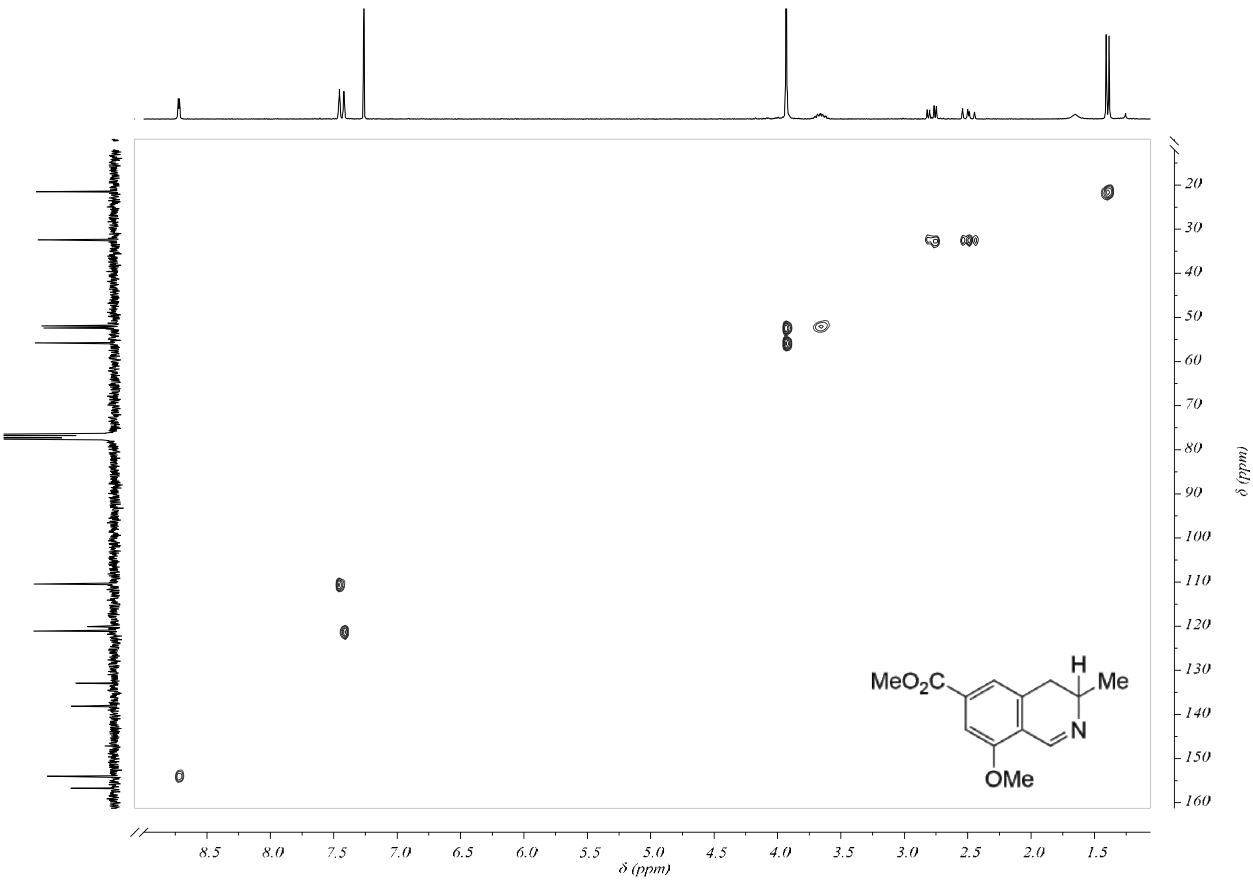

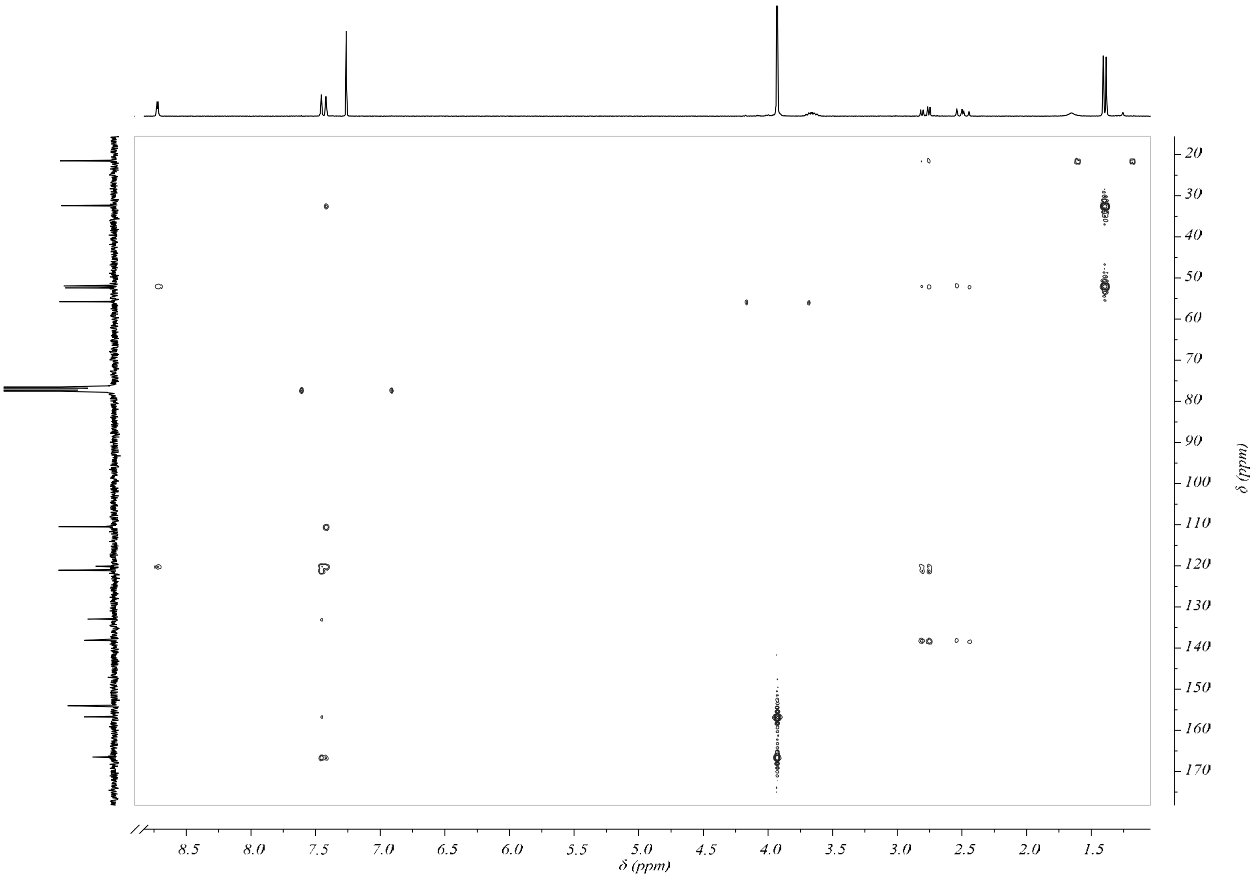

Figure S28. HSQC (top) and HMBC (bottom) spectra of compound **33** in $CDCl_3$.

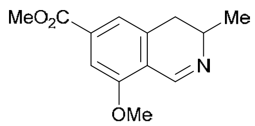

Acquisition Parameter

Source Type	ESI	Ion Polarity	Positive	Set Nebulizer	3.0 Bar
Focus	Not active	Set Capillary	4000 V	Set Dry Heater	200 °C
Scan Begin	100 m/z	Set End Plate Offset	-500 V	Set Dry Gas	6.0 l/min
Scan End	1300 m/z	Set Collision Cell RF	250.0 Vpp	Set Divert Valve	Source

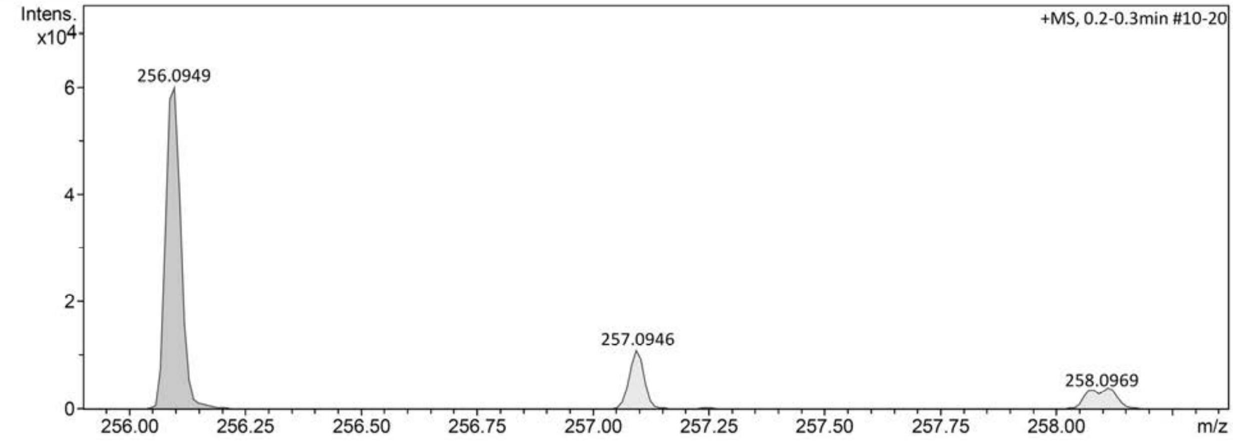

Meas. m/z	#	Ion Formula	m/z	err [ppm]	mSigma	# mSigma	Score	rdb	e⁻ Conf	N-Rule
256.0949	1	C13H15NNaO3	256.0944	-2.0	29.3	1	100.00	6.5	even	ok

Figure S29. High-resolution mass spectrum of compound **33**.

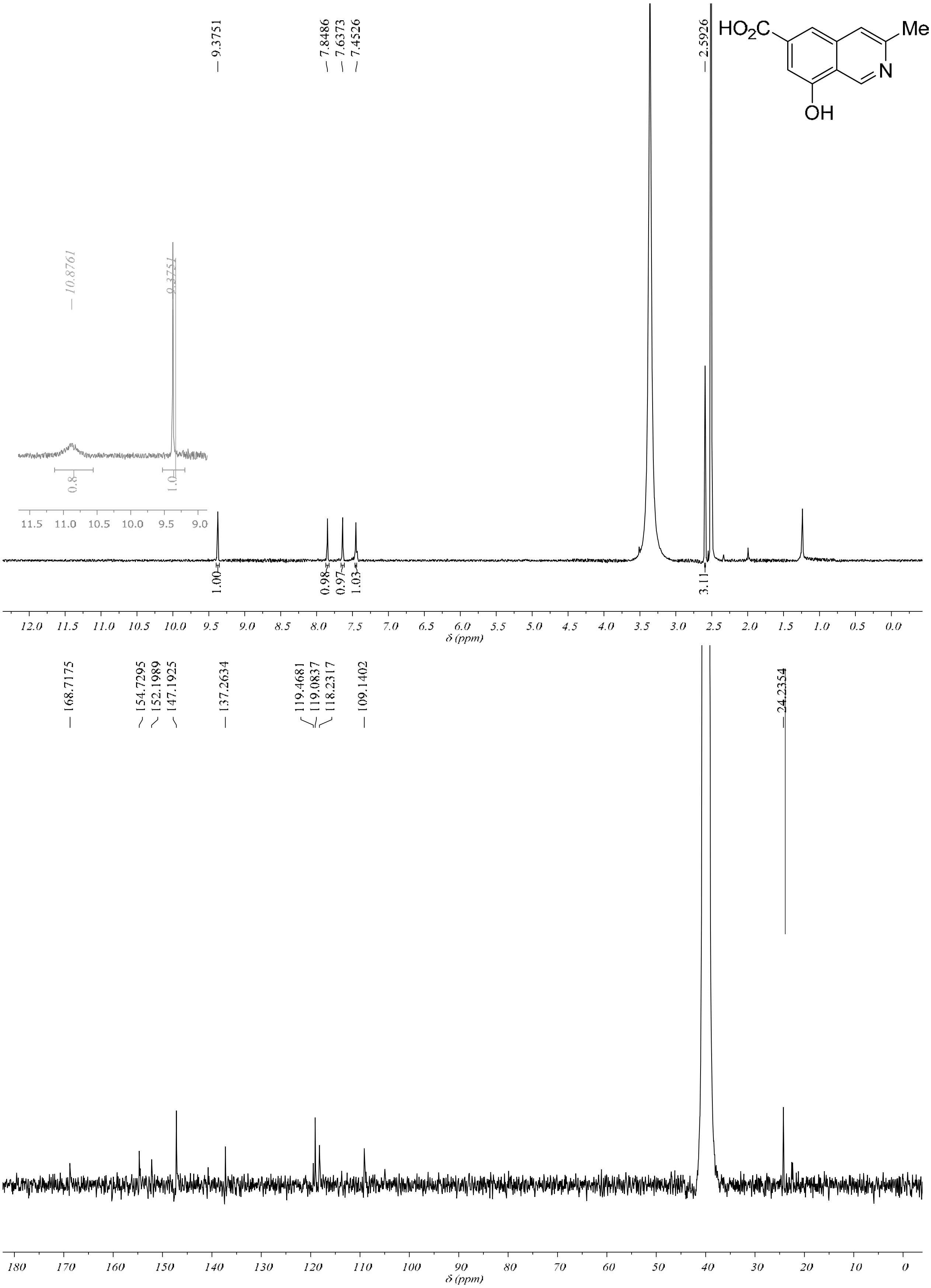

Figure S30. 400 MHz ^{1}H (top) and 100 MHz ^{13}C (bottom) NMR spectra of ampullosine (**4**) in DMSO-d_6.

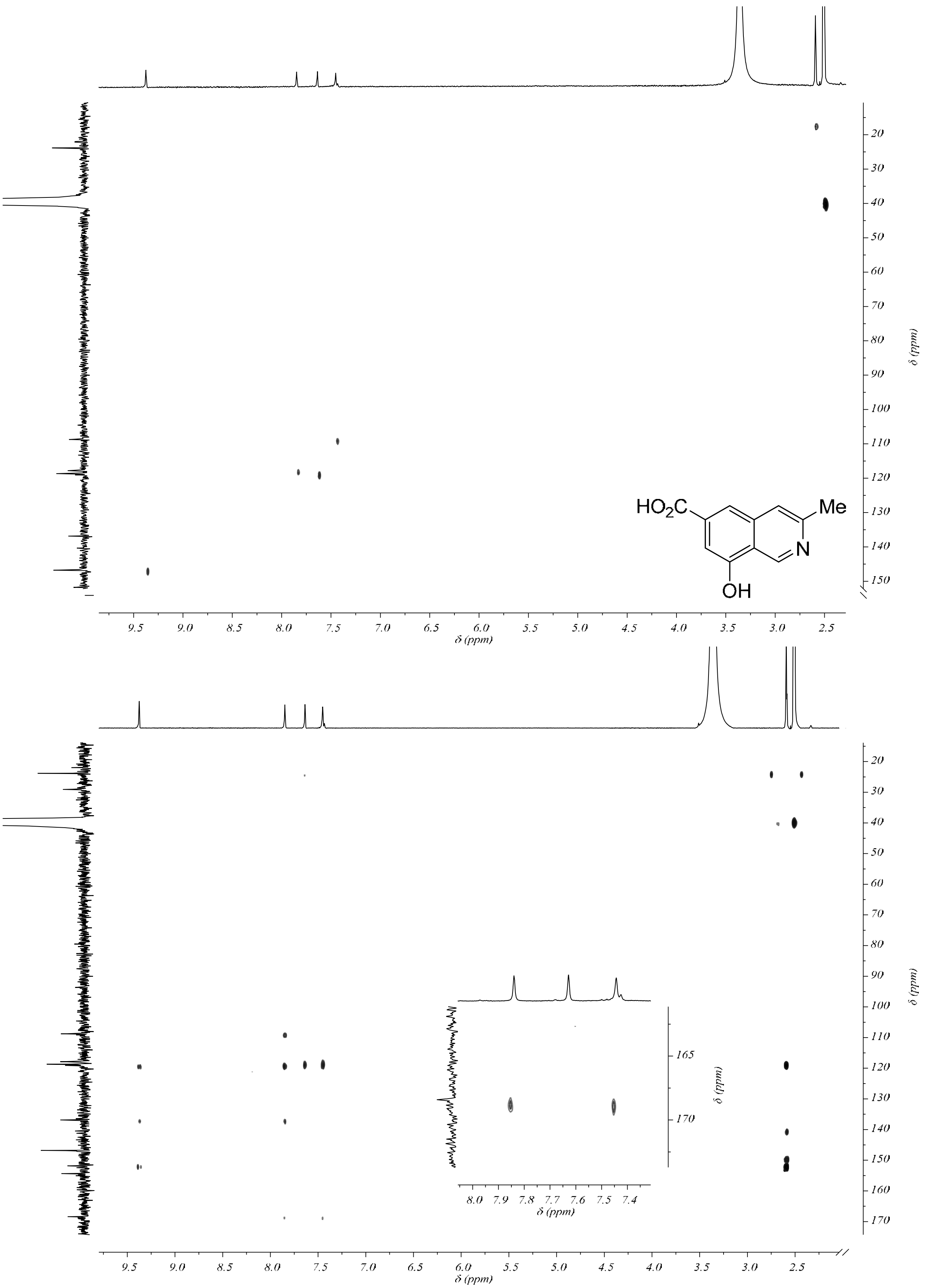

Figure S31. HSQC (top) and HMBC (bottom) spectra of ampullosine (**4**) in DMSO-d_6.

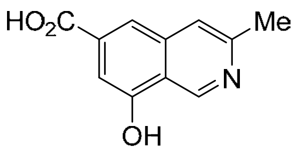

Acquisition Parameter

Source Type	ESI	Ion Polarity	Positive	Set Nebulizer	3.0 Bar
Focus	Not active	Set Capillary	4000 V	Set Dry Heater	200 °C
Scan Begin	100 m/z	Set End Plate Offset	-500 V	Set Dry Gas	6.0 l/min
Scan End	1400 m/z	Set Collision Cell RF	250.0 Vpp	Set Divert Valve	Source

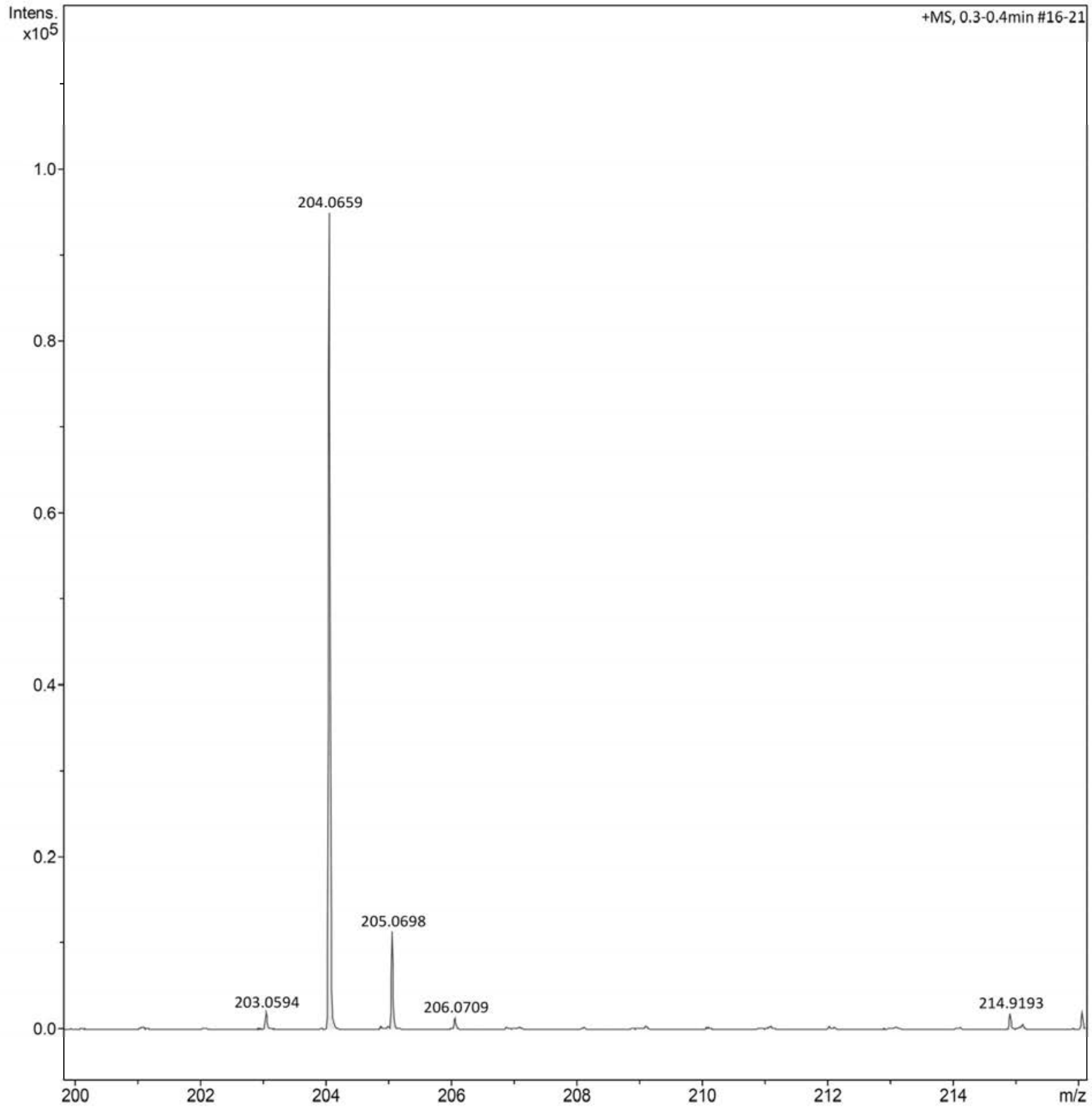

Meas. m/z	#	Ion Formula	m/z	err [ppm]	mSigma	# mSigma	Score	rdb	e⁻ Conf	N-Rule
204.0659	1	C11H10NO3	204.0655	-1.6	3.1	1	100.00	7.5	even	ok

Figure S32. High-resolution mass spectrum of compound **4**.

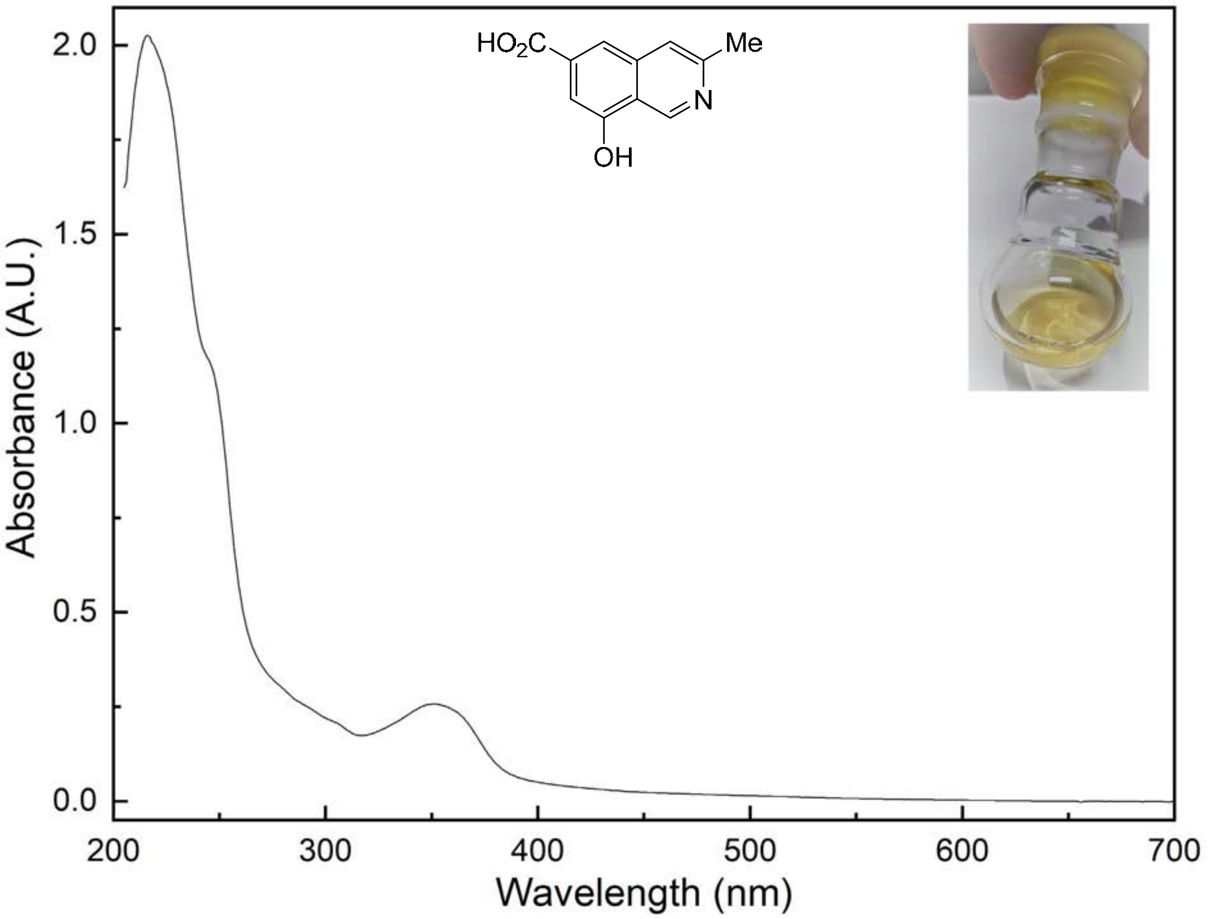

Figure S33. UV-Vis spectrum of ampullosine (**4**) in MeOH.

Table S1. Spectroscopic comparison of ^{1}H NMR data of permethylampullosine (**5**).

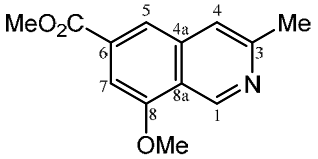

Per-methylampullosine (5)

Proton number	Quang *et al.*[1] (MeOH-d_4, 400 MHz)	This report (MeOH-d_4, 400 MHz)	This report ($CDCl_3$, 300 MHz)	Δδ (MeOH-d_4, 400 MHz)
1	9.45 (s)	9.32 (s)	9.56 (s)	+0.13
4	7.73 (s)	7.58 (s)	7.50 (s)	+0.15
5	8.11 (br s)	7.94 (s)	8.03 (br s)	+0.17
7	7.48 (br s)	7.34 (s)	7.37 (d, J = 1.0)	+0.14
3-Me	2.68 (s)	2.63 (s)	2.71 (s)	+0.05
6-CO_2Me	3.98 (s)	3.97 (s)	3.99 (s)	+0.01
8-OMe	4.11 (s)	4.05 (s)	4.07 (s)	+0.06

Table S2. Spectroscopic comparison of ^{13}C NMR data of permethylampullosine (**5**).

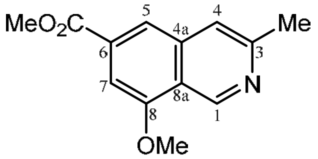

Per-methylampullosine (5)

Carbon number	Quang *et al.*[1] (MeOD-d_4, 400 MHz)	This report (MeOD-d_4, 400 MHz)	This report ($CDCl_3$, 300 MHz)	Δδ (MeOD-d_4, 400 MHz)
1	147.6 (d)	147.5	147.4	+0.1
3	153.9 (s)	153.7	153.5	+0.2
4	121.1 (d)	121.0	119.1	+0.1
4a	138.8 (s)	138.6	137.2	+0.2
5	122.0 (d)	121.8	121.2	+0.2
6	134.3 (s)	134.0	132.2	+0.3
7	104.9 (d)	104.7	103.6	+0.2
8	158.2 (s)	158.0	156.9	+0.2
8a	121.7 (s)	121.5	120.6	+0.3
3-Me	23.5 (q)	23.5	24.3	0.0
6-*C*O_2Me	167.9 (s)	167.8	166.9	+0.1
6-CO_2*Me*	53.1 (q)	53.1	52.7	0.0
8-OMe	56.6 (q)	56.5	56.0	+0.1

Table S3. Spectroscopic comparison of NMR data of ampullosine (**4**).

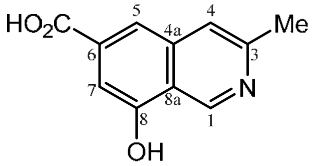

Ampullosine (4)

Position	Quang *et al.* (DMSO-d_6, 400 MHz) δ_H	Quang *et al.* δ_C	This work (DMSO-d_6, 400 MHz) δ_H	This work δ_C	$\Delta\delta_C$ (DMSO-d_6, 400 MHz)
1	9.36 (s)	146.7 d	9.37 (s)	147.2	-0.5
3	-	151.5 s	-	152.2	-0.7
4	7.61(s)	118.6 d	7.64 (br s)	119.1	-0.5
4a	-	136.8 s	-	137.3	-0.5
5	7.80 (br s)	117.4 d	7.85 (s)	118.3	-0.9
6	-	138.9 s	-	137.3	+1.6
7	7.45 (br s)	109.1 d	7.45(s)	109.2	-0.1
8	-	154.2 s	-	154.8	-0.6
8a	-	118.9 s	-	119.5	-0.6
3-Me	2.59 (s)	23.8 q	2.59 (s)	24.2	-0.4
6-CO_2H	-	168.3 s	-	168.3	0.0
OH			10.88 (s)<br>3.35 (s)	-	

References

1. Quang, D. N.; Schmidt, J.; Porzel, A.; Wessjohann, L.; Haid, M.; Arnold, N. *Nat. Prod. Commun.* **2010**, *5*, 869–872.
